# Functionalization of octaspherosilicate (HSiMe_2_O)_8_Si_8_O_12_ with buta-1,3-diynes by hydrosilylation

**DOI:** 10.1038/s41598-023-41461-2

**Published:** 2023-08-31

**Authors:** Kinga Stefanowska, Jakub Nagórny, Jakub Szyling, Adrian Franczyk

**Affiliations:** 1 Center for Advanced Technology, Adam Mickiewicz University, Uniwersytetu Poznanskiego 10, 61-614 Poznan, Poland; 2 Faculty of Chemistry, Adam Mickiewicz University, Uniwersytetu Poznanskiego 8, 61-614 Poznan, Poland

**Keywords:** Chemistry, Catalysis, Inorganic chemistry, Materials chemistry, Organic chemistry, Chemical synthesis

## Abstract

Hydrosilylation with octaspherosilicate (HSiMe_2_O)_8_Si_8_O_12_ (**1**) has provided hundreds of molecular and macromolecular systems so far, making this method the most popular in the synthesis of siloxane-based, nanometric, cubic, and reactive building blocks. However, there are no reports on its selective reaction with 1,3-diynes, which allows for the formation of new products with unique properties. Therefore, herein we present an efficient protocol for monohydrosilylation of symmetrically and non-symmetrically 1,4-disubstituted buta-1,3-diynes with **1**. The compounds obtained bear double and triple bonds and other functionalities (e.g., Br, F, OH, SiR_3_), making them highly desirable, giant building blocks in organic synthesis and material chemistry. These compounds were fully characterized by ^1^H, ^13^C, ^29^Si, 1D NOE, ^1^H–^13^C HSQC NMR, FT–IR, and MALDI TOF MS, EA, UV–Vis, and TGA analysis. The TGA proved their high thermal stability up to 427 ℃ (T_d_^10%^) for compound **3j**.

## Introduction

Polyhedral oligospherosilicate (HSiMe_2_O)_8_Si_8_O_12_ (**1**) is a commercially available compound which, thanks to its unique three-dimensional, cubic, and nanometric structure, its physicochemical, and biological properties, and the possibility for its functionalization by hydrosilylation reactions, is of great interest to researchers from academia and industry^1–11^. This is highlighted by the huge number of scientific and patent publications on it, which currently exceeds five hundred. The literature describes **1** as a reactive platform for the synthesis of multifunctional, hybrid (inorganic–organic) molecules or macromolecules which have been applied in many different fields^12–24^. The most recognized works focus on the preparation of liquid crystals^25–29^, coating materials^30–33^, electrolytes for lithium batteries^34–36^, gate dielectric for organic thin film transistors^37,38^, materials for imprint lithography^39–42^, anticancer drug carriers^43^, optoelectronic materials^44–59^, dental materials^60,61^, dyes^62,63^, detectors for explosives^64^, surface acoustic wave sensors^65^, surfactants^66,67^, catalysts^68,69^, Janus particles^70^, nanoreactors^71,72^, self-healing materials^73–75^, polymers^76–80^, membranes^81,82^, and functional porous materials for gas transport^83^ or proton exchange^84^. Such a wide application of **1**, especially in comparison to its structural analog polyhedral oligosilsesquioxane (POSS) H_8_Si_8_O_12_, results from a much higher activity of **1** in the hydrosilylation process. In the case of H_8_Si_8_O_12_, the close proximity of the silicon-organic cage to the Si–H bond, as well as its poor solubility in organic solvents, makes it much less reactive and thus less often used. Both compounds should be considered as representatives of the same POSS family, since they meet the general formula (RSiO_3/2_)_n_ (where for octaspherosilicate **1** R = OSiMe_2_H) typical for POSS, and as a consequence, they possess a cubic cage in their structure. Therefore, the differentiation of their properties will depend only on the type of eight R groups. The modification of **1** led to novel systems that were obtained mainly by industrially applied hydrosilylation reaction, which facilitates the functionalization of systems containing Si–H bonds with reagents possessing carbon–carbon double (C=C) or triple bonds (C≡C)^85–87^. One invaluable advantage of this process results from the fact that it is tolerant to a wide spectrum of functional groups. This makes it a powerful and versatile approach. By the application of one compound (in this case **1**), hundreds of products with distinctly different physicochemical and biological properties can be obtained. Although hydrosilylation of alkenes and alkynes with **1** has been well studied^88–95^, there are no reports focusing on the hydrosilylation of C≡C bonds in symmetrically and non-symmetrically 1,4-disubstituted buta-1,3-diynes. The development of an octaspherosilicate **1** functionalization method is justified primarily due to the fact that it produces new products with novel and unique properties, especially those applied in optoelectronics. Additionally, using 1,3-diynes in synthesis, it is possible to introduce both the unsaturated C=C bond (similar to the reaction with alkynes) and the C≡C triple bond into the product structure in a single reaction step. The C≡C triple bond can be subsequently modified in e.g., hydrosilylation or hydroboration reactions, providing new compounds with unique properties.

The lack of reports on the hydrosilylation of conjugated 1,3-diynes^96^ with **1** might be caused by the fact that their selective transformation is a challenging task. Due to the presence of two C≡C bonds in the 1,3-diyne structure and at the same time, eight Si–H bonds in octaspherosilicate **1**, the formation of many products is possible (silylated 1,3-enynes, 1,3-dienes, allenes, polymers, and cyclic compounds), and a complex mixture of various products is often obtained^97–105^. To carry out the hydrosilylation in a regio- and stereoselective manner, many factors, such as the type of catalyst, the structure of reagents, and the process conditions, need to be carefully selected. While the hydrosilylation of alkynes with silanes or silsesquioxanes, including **1**, has been described in several papers^88–95^, there are only a few examples of the hydrosilylation of buta-1,3-diynes^98,99,101,106–110^, among which two have described the reaction with silsesquioxanes (HSiMe_2_O)(*i*-Bu)_7_Si_8_O_12_ and (HSiMe_2_O)_3_R′_7_Si_7_O_9_. The first research was focused on the hydrosilylation of 1,4-symmetrically substituted 1,3-diynes with monofunctional silsesquioxane (HSiMe_2_O)(*i*-Bu)_7_Si_8_O_12_ in the presence of Pt catalysts (Karstedt’s catalyst (Pt_2_(dvs)_3_), Pt(PPh_3_)_4_, PtO_2_, or Pt/SDB (SDB- styrene-divinylbenzene copolymer))^99^. It was found that the process selectivity depended on the catalyst type and the reagent structure and its concentration. Hydrosilylation of sterically hindered buta-1,3-diynes (2,2,7,7-tetramethylocta-3,5-diyne, 2,7-di(trimethylsiloxy)-2,7-dimethylocta-3,5-diyne using equimolar quantities of reagents in the presence of Karstedt’s catalyst led to the formation of silsesquioxane-substituted 3-en-1-ynes with high selectivity (93–100%). Meanwhile, the monohydrosilylation of linear hexa-2,4-diyne and less bulky diynes e.g., 1,4-diphenylbuta-1,3-diyne, 1,4-dibromophenylbuta-1,3-diyne, 1,6-bis(morpholino)hexa-2,4-diyne, and 10,12-docosadiyndioic acid dimethyl ester using the same catalyst resulted in a mixture of mono- and bishydrosilylated products. Bisadducts were successfully synthesized through the hydrosilylation of less sterically hindered 1,3-diynes with silsesquioxane (HSiMe_2_O)(*i*-Bu)_7_Si_8_O_12_ applying a molar ratio of reagents of 2:1 in the presence of Karstedt’s catalyst. The second article discusses the hydrosilylation of both symmetrical and unsymmetrical buta-1,3-diynes with trifunctional incompletely condensed silsesquioxanes (IC-POSSs (HSiMe_2_O)_3_R′_7_Si_7_O_9_ with *i*-Bu (R′ = *i*-C_4_H_9_) or *i*-Oct (R′ = (H_3_C)_3_CH_2_C(H_3_C)HCH_2_C) substituents. The reactions were performed in the presence of Karstedt’s catalyst^106^. For symmetrically substituted 1,3-diynes (1,4-diphenylbuta-1,3-diyne, 1,4-di(4-fluorophenyl)buta-1,3-diyne, and 1,4-bis(thiophen-3-yl)buta-1,3-diyne), an excess of diyne (6–12 mol) to silsesquioxanes was required. On the other hand, using a stoichiometric amount of 1,3-diyne in the hydrosilylation of unsymmetrical diynes with Si(*i*-Pr)_3_ groups led to the formation of monohydrosilylated products with a very high selectivity of 99%.

The excellent results from the above-described research encouraged us to take one step further and investigate a much more challenging reagent **1** with eight Si–H bonds. The selective addition of eight Si–H bonds of octaspherosilicate **1** to only one of two C≡C bonds in 1,3-diynes is much more complex, and experience gained from simpler models seemed to be essential to accomplish this task. Moreover, the advantage of using octaspherosilicate over mono- and trifunctional silsesquioxanes is that we can introduce up to 16 identical or different functional groups into a hybrid, cubic structure in a single reaction step. Additionally, the physicochemical properties of the resulting compounds will be determined by the type of substituents attached to the C=C and C≡C bonds, as well as the inorganic core. In mono- and trifunctional systems, 7 alkyl groups also have a strong impact on how they are defined. Keeping in mind very rich applications of alkenyl-octaspherosilicates^59^, the octaspherosilicates with 3-en-1-yl groups obtained here or their derivatives are highly desirable and can become systems with similar advantages applicable in similar fields of science. Therefore, herein we present efficient synthetic methods for obtaining new octaspherosilicates with 3-en-1-yl groups.

## Results and discussion

**Figure 1:**
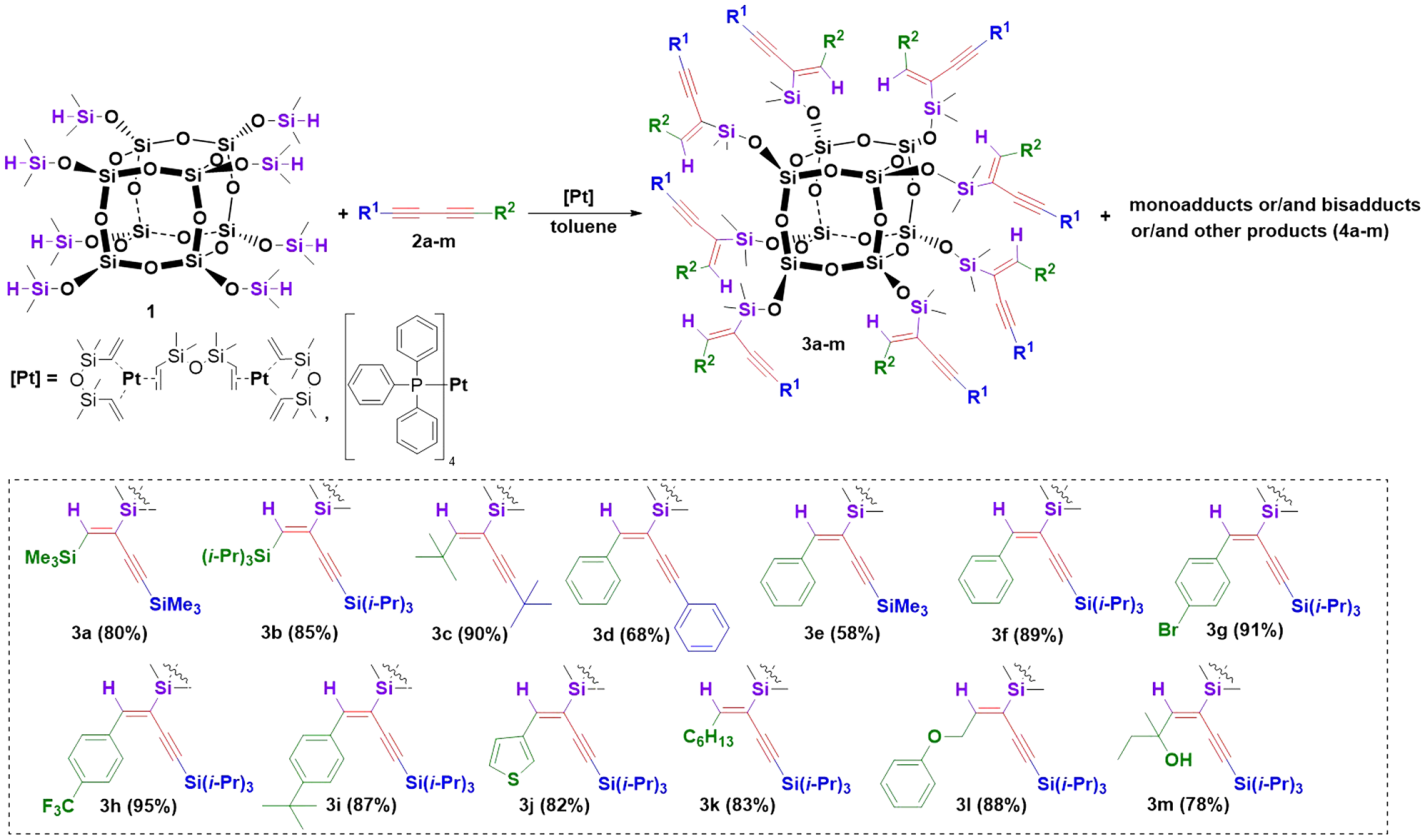
Hydrosilylation of buta-1,3-diynes **2a–m** with octaspherosilicate **1**. Isolated yields of obtained products are presented in brackets.

**Figure 2:**
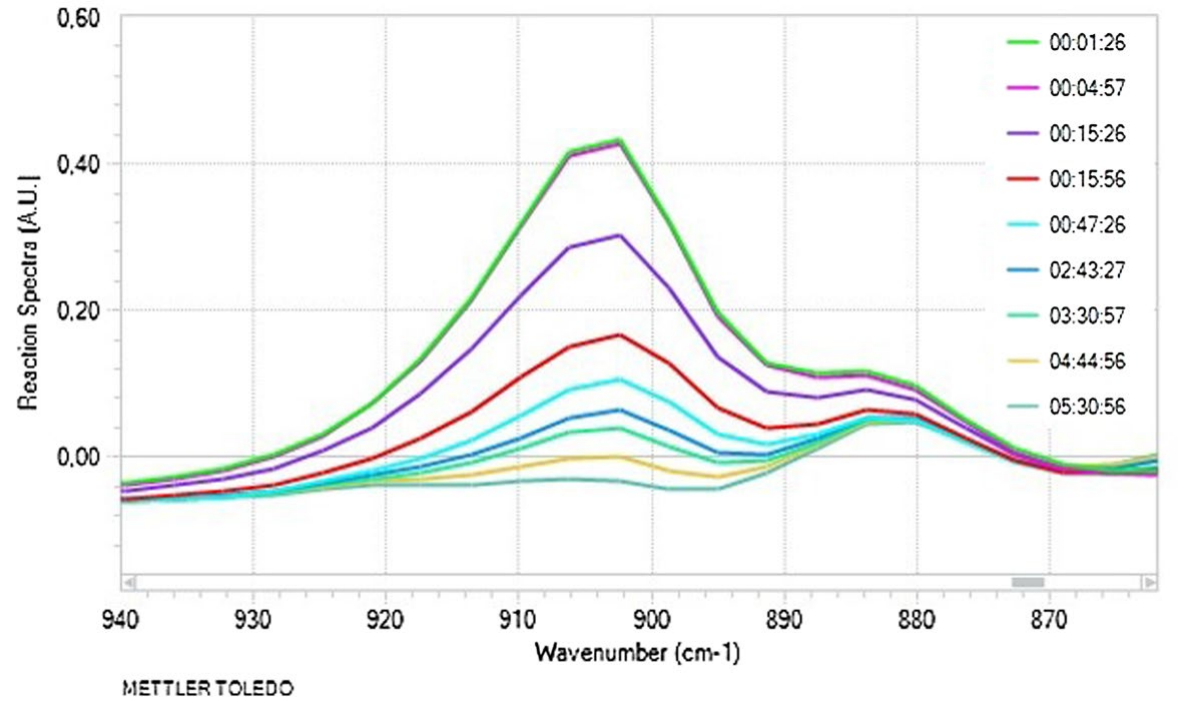
Hydrosilylation of deca-1,3-diyn-1-yl-tri(*iso*propyl)silane (**2k**) with **1** monitored by in situ FT-IR.

**Figure 3:**
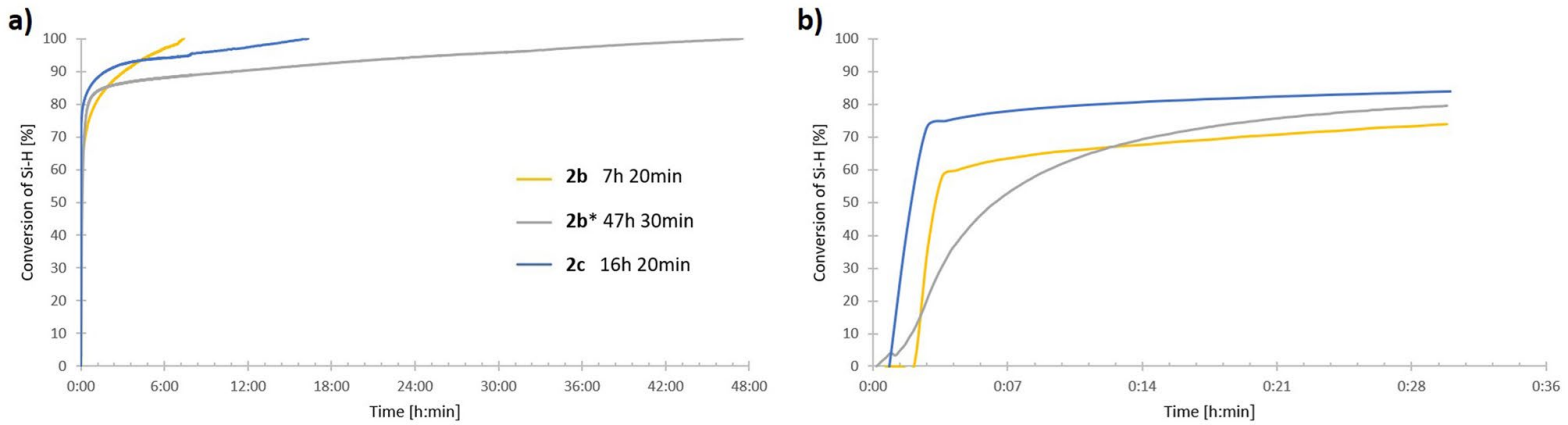
Kinetic plots for hydrosilylation of buta-1,3-diyne **2b-c** with **1** in the presence of Karstedt’s (0.8 mol% Pt) catalyst and Pt(PPh_3_)_4_ (0.8 mol% Pt, marked with an asterisk) determined by in situ FT-IR. (**a**) Full times of the processes are presented, (**b**) the first 30 min of the processes are presented.

**Figure 4:**
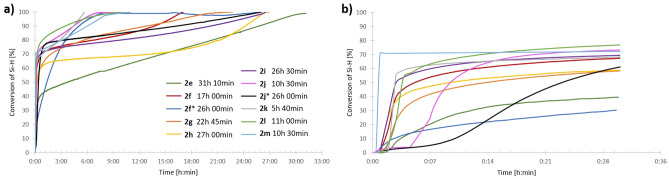
Kinetic plots for hydrosilylation of buta-1,3-diyne **2e-m** with **1** in the presence of Karstedt’s (0.8 mol% Pt) catalyst and Pt(PPh_3_)_4_ (0.8 mol% Pt, marked with an asterisk) determined by in situ FT-IR. (**a**) Full times of the processes are presented, (**b**) the first 30 min of the processes are presented.

For the study, we conducted reactions of thirteen, structurally different 1,3-diynes (**2a–m**) with octaspherosilicate (HSiMe_2_O)_8_Si_8_O_12_ (**1**) (Fig. 1, Table 1). The processes were carried out in the presence of commercially available Karstedt’s catalyst and Pt(PPh_3_)_4_ in an air atmosphere, without any purification of the acquired chemicals, at different temperatures (r.t-100 ℃). The progress of the hydrosilylation process was monitored in real-time by in situ FT-IR spectroscopy (by tracking changes in the area of the band at 880–930 cm^−1^, assigned to stretching vibrations of the Si–H bond). The representative illustration of the measurements provided by in situ FT-IR showing the lowering of the intensity of signals from the Si–H group during the hydrosilylation process is presented in Fig. 2. The decay in the band was observed with time and on this basis, the conversions of **1** in the appropriate reactions were determined. As a result, kinetic plots for hydrosilylation of 1,3-diynes **2b–c** (Fig. 3) and **2e–m** (Fig. 4) were obtained. On the other hand, the process selectivity was calculated using ^1^H and ^29^Si NMR analysis. All these analytical methods allowed us to examine the influence of the 1,3-diyne structure and various reaction conditions on the stereoselectivity and progress of the hydrosilylation process. The use of in situ FT–IR spectroscopy was crucial for determining the time required to obtain the total conversion of the reagents. The collected data is summarized in Table 1.

**Table 1:** Hydrosilylation of buta-1,3-diynes **2a–m** with octaspherosilicate **1**.

Entry	Diyne	R^1^	R^2^	[**1**]:[**2**]:[Pt]	[Pt]	Temp. [℃]	Reaction time	Conv. of SiH [%]	Selectivity of 3/4 [%]
1	**2a**	SiMe_3_	SiMe_3_	1:8: 8 × 10^–3^	Pt_2_(dvs)_3_	100	30 min	> 99	Complex mixture
2							24 h 00 min	77	95/5
3				1:16: 8 × 10^–3^		r.t.	24 h 00 min^a^	79	> 99/0
4							96 h 00 min^a^	82	> 99/0
5				1:32: 8 × 10^–3^		100	24 h 00 min^a^	> 99	Complex mixture
6				1:8:2 × 10^–1^	Pt(PPh_3_)_4_	40	24 h 00 min^a^	73	72/28
7							72 h 00 min^a^	> 99	72/28
8				1:16:2 × 10^–1^			24 h 00 min^a^	77	> 99/0
9							72 h 00 min^a^	94	> 99/0
10				1:8:8 × 10^–2^		100	24 h 00 min^a^	> 99	Complex mixture
11	**2b**	Si(*i*-Pr)_3_	Si(*i*-Pr)_3_	1:8:8 × 10^–3^	Pt_2_(dvs)_3_	100	7 h 20 min	> 99	96/4
12				1:10:8 × 10^–3^			24 h 00 min^a^	> 99	99/1
13				1:8:8 × 10^–3^	Pt(PPh_3_)_4_	100	47 h 30 min	> 99	93/7
14	**2c**	*t*-Bu	*t*-Bu	1:8:8 × 10^–3^	Pt_2_(dvs)_3_	100	48 h 00 min^a^	> 99	95/5
15				1:10:8 × 10^–3^			16 h 20 min	> 99	> 99/0
16	**2d**	Ph	Ph	1:16:2 × 10^–1^	Pt(PPh_3_)_4_	40	96 h 00 min^a^	> 99	> 99/0
17	**2e**	SiMe_3_	Ph	1:8:8 × 10^–3^	Pt_2_(dvs)_3_	100	31 h 10 min	> 99	98/2
18				1:8:8 × 10^–2^	Pt(PPh_3_)_4_	100	48 h 00 min^a^	> 99	97/3
19	**2f**	Si(*i*-Pr)_3_	Ph	1:8:8 × 10^–3^	Pt_2_(dvs)_3_	100	17 h 00 min	> 99	> 99/0
20				1:8:8 × 10^–3^	Pt(PPh_3_)_4_	100	26 h 00 min	> 99	> 99/0
21	**2g**	Si(*i*-Pr)_3_	PhBr-4	1:8:8 × 10^–3^	Pt_2_(dvs)_3_	100	22 h 45 min	> 99	> 99/0
22	**2h**	Si(*i*-Pr)_3_	PhCF_3_-4	1:8:8 × 10^–3^	Pt_2_(dvs)_3_	100	27 h 00 min	> 99	> 99/0
23	**2i**	Si(*i*-Pr)_3_	Ph-*t*-Bu-4	1:8:8 × 10^–3^	Pt_2_(dvs)_3_	100	26 h 30 min	> 99	> 99/0
24	**2j**	Si(*i*-Pr)_3_	Thienyl	1:8:8 × 10^–3^	Pt_2_(dvs)_3_	100	10 h 30 min	> 99	> 99/0
25				1:8:8 × 10^–3^	Pt(PPh_3_)_4_	100	26 h 00 min	> 99	> 99/0
26	**2k**	Si(*i*-Pr)_3_	C_6_H_13_	1:8:8 × 10^–3^	Pt_2_(dvs)_3_	100	5 h 40 min	> 99	> 99/0
27	**2l**	Si(*i*-Pr)_3_	CH_2_OPh	1:8:8 × 10^–3^	Pt_2_(dvs)_3_	100	11 h 00 min	> 99	> 99/0
28	**2m**	Si(*i*-Pr)_3_	C(CH_3_)(OH)C_2_H_5_	1:8:8 × 10^–3^	Pt_2_(dvs)_3_	100	10 h 30 min	> 99	> 99/0

Reaction conditions: m_s(**1)**_/V_tol._ = 50 mg mL^−1^ (where m_S(**1)**_ is mass of the substance **1**). Conversion of reagents was determined by in situ FT-IR and confirmed by ^1^H NMR and FT-IR. The selectivity for all experiments was determined by ^1^H, ^13^C, and ^29^Si NMR. For selected products, 1D NOE, ^1^H-^13^C HSQC NMR was made.

^a^The reaction time was not determined based on in-situ FT–IR experiments.

First, we investigated the hydrosilylation of symmetrical 1,4-bis(trimethylsilyl)buta-1,3-diyne (**2a**) with octaspherosilicate (**1**) in the presence of Karstedt’s catalyst and Pt(PPh_3_)_4_ (Table 1, entries 1–10). The reaction in the presence of Karstedt’s catalyst (8 × 10^–3^ mol of Pt per mol of SiH, the ratio [**1**]:[**2**]  = 1:8, at 100 ℃, in toluene) required only 30 min to reach complete conversion but resulted in a complex mixture of mono- and bisadducts, as well as other side-products (Table 1, entry 1). The same set of reagents tested in the presence of Pt(PPh_3_)_4_ (10^–2^ mol of Pt per mol of SiH, similar reaction conditions (Table 1, entry 10)) gave a complex mixture of products after 24 h. Thus, both catalysts turned out to be non-selective for the hydrosilylation reaction of **2a** under the tested conditions at 100 ℃ and reagents ratio of [**1**]:[**2**]  = 1:8. To optimize the hydrosilylation of **2a** with **1**, we started by lowering the reaction temperature to room temperature, using the Karstedt’s catalyst at a concentration of 8 × 10^–3^ mol of Pt per mol of SiH (Table 1, entry 2). This led to the formation of product **3a** with a selectivity of 95%, although the conversion of **1** after 24 h was only 77%. Product **3a** was selectively obtained in the presence of Karstedt’s catalyst using a reagent ratio of [**1**]:[**2**]  = 1:16, but the conversion was still incomplete (82%), even after 96 h (Table 1, entry 4). It was proved that room temperature was crucial to achieving high process selectivity to product **3a** when Karstedt’s catalyst was used. At 100 ℃, even with a four-fold excess of diyne **2a**, a mixture of compounds was observed (Table 1, entry 5). Similar findings were observed using less active Pt(PPh_3_)_4_. The lower the temperature, the higher the selectivity was observed (72% toward product **3a** at 40 °C), (Table 1, entries 6–7). Notably, the higher catalyst concentration of 2 × 10^–1^ mol of Pt per mol of Si–H was used in this reaction. Product **3a** was selectively obtained with a reaction yield of 94% by carrying out the process for 72 h at 40 ℃ and using a two-fold excess of 1,3-diyne **2a** per Si–H bond (Table 1, entry 9). Optimized conditions allowed the desired product **3a** to be obtained with a very good 80% of isolated yield. It transpired that the use of a high concentration of Pt(PPh_3_)_4_ (which is less active than Karstedt’s catalyst) at a low temperature and a small excess of 1,3-diyne was the solution for the highly selective formation of the targeted product. We would like to underline that selectivity was the overriding goal for our studies, as the formation of by-products makes the separation of targeted compounds very difficult, due to their structural similarity to products **3** and their high molecular weights. In the next step of our research, the hydrosilylation of more sterically crowded 1,4-bis(tri(*iso*propyl)silyl)buta-1,3-diyne (**2b**) and 2,2,7,7-tetramethylocta-3,5-diyne (**2c**) with **1** was performed. The processes were carried out in the presence of Karstedt’s catalyst (8 × 10^–3^ mol of Pt per mol of SiH) with the use of reagents ratio [**1**]:[**2**] = 1:8 at 100 ℃, leading to the products **3b** and **3c** with the 96% and 95% of selectivity, respectively (Table 1, entries 11 and 14). The progress of hydrosilylation of diyne **2b** with **1** was controlled by the in situ FT-IR spectroscopy and showed that the reaction took 7 h and 20 min, while for diyne **2c**, 48 h were needed for the total conversion of the reagents. To increase the selectivity of the processes with **2b** and **2c**, the reactions were carried out using a little excess of **2** ([**1**]:[**2**]  = 1:10). The processes provided products **3b** and **3c** exclusively. The reaction with **2b** was carried out for 24 h, while the process with **2c** was completed in 16 h and 20 min (Table 1, entries 12 and 15).

In addition, the kinetics of the hydrosilylation processes of **2b** with octaspherosilicate **1** were compared for both catalysts Pt_2_(dvs)_3_ and Pt(PPh_3_)_4_ under the same reaction conditions (Fig. 3a and b). It turned out that the process carried out in the presence of Pt(PPh_3_)_4_ also resulted in the formation of product **3b** with lower selectivity, and it took an additional 40 h compared to the application of a more active Karstedt’s catalyst. Another example of hydrosilylation involved a symmetrical 1,4-diphenylbuta-1,3-diyne (**2d**). Due to the fact that the use of Karstedt’s catalyst led to the formation of a complex mixture of products, based on the above-described optimized reaction conditions for 1,3-diyne **3a**, the hydrosilylation of **2d** with **1** was carried out in the presence of Pt(PPh_3_)_4_ at 40 °C, using an excess of diyne ([**1**]:[**2**]  = 1:16). The total conversion of reagents and excellent selectivity to **3d** was observed after 96 h (Table 1, entry 16). Subsequently, the hydrosilylation of a series of unsymmetrically substituted diynes **2e**-**2m** with one tri(*iso*propylsilyl) group in the structure with octaspherosilicate **1** was studied (Table 1, entries 17–28). The processes were carried out in the presence of Karstedt’s catalyst (8 × 10^–3^ mol of Pt per mol of SiH) with the ratio of reagents [**1**]:[**2**]  = 1:8 at 100 ℃, leading selectively to products **3**. The reactions were monitored using in situ FT-IR spectroscopy, which showed that the rate of hydrosilylation of the C≡C bond was strongly dependent on the structure of the 1,3-diyne. For reagent **2k**, the reaction finished in 5 h and 40 min, while for **2j**, **2l**, and **2m**, 10–11 h were necessary to observe full conversion. In the case of hydrosilylation of (phenylbuta-1,3-diyn-1-yl)tri(*iso*propyl)silane (**2f**), the process was completed in 17 h. Lower reaction rates (22–31 h) were found for hydrosilylation of diynes **2e**, **2g**–**i**. The kinetic plots obtained from the in situ FT–IR measurements (Fig. 4a and b) illustrated that after the addition of the catalyst to the reaction mixture and heating, the fast consumption of reagents took place (62–76%), and finally, the reaction rates decreased slightly due to the lower concentration of the reagents. Similar trends were observed for the hydrosilylation of diynes **2b** and **2c**. In contrast, the hydrosilylation of **2e** was characterized by a short initiation period, where 40% of Si–H conversion was observed in just 33 min, followed by moderate consumption of the reagents throughout the reaction. The hydrosilylation of diynes **2f** and **2j** with octaspherosilicate **1** was also tested in the presence of Pt(PPh_3_)_4_ (Table 1, entries 20 and 25). However, due to the steric hindrances in the structures of both the catalyst and reactants, the time required to achieve full conversion of the Si–H bond was increased by 9 h for **2f** and 15 h and 30 min for **2j** compared to the same reactions carried out using Pt_2_(dvs)_3_ as a catalyst. Nonetheless, the kinetic plots for both processes were consistent with those for reactions carried out in the presence of Karstedt’s catalyst. Products **3e**–**m** were isolated in 58–95% yields. The synthetic procedures described above were both efficient and straightforward, allowing for the preparation of octafunctional spherosilicates that possess eight alkenyl substituents, each with functional groups like 4-bromophenyl, thienyl, silyl, and hydroxyl. These systems are prone to further modification via hydrosilylation, hydroboration, or other chemical reactions occurring on both unsaturated bonds and functional groups (polymerization reactions, Suzuki–Miyaura, Sonogashira, Heck, and Hiyama couplings or for the preparation of molecular and macromolecular star-shaped hybrids or reactive or unreactive nanofillers).

**Figure 5:**
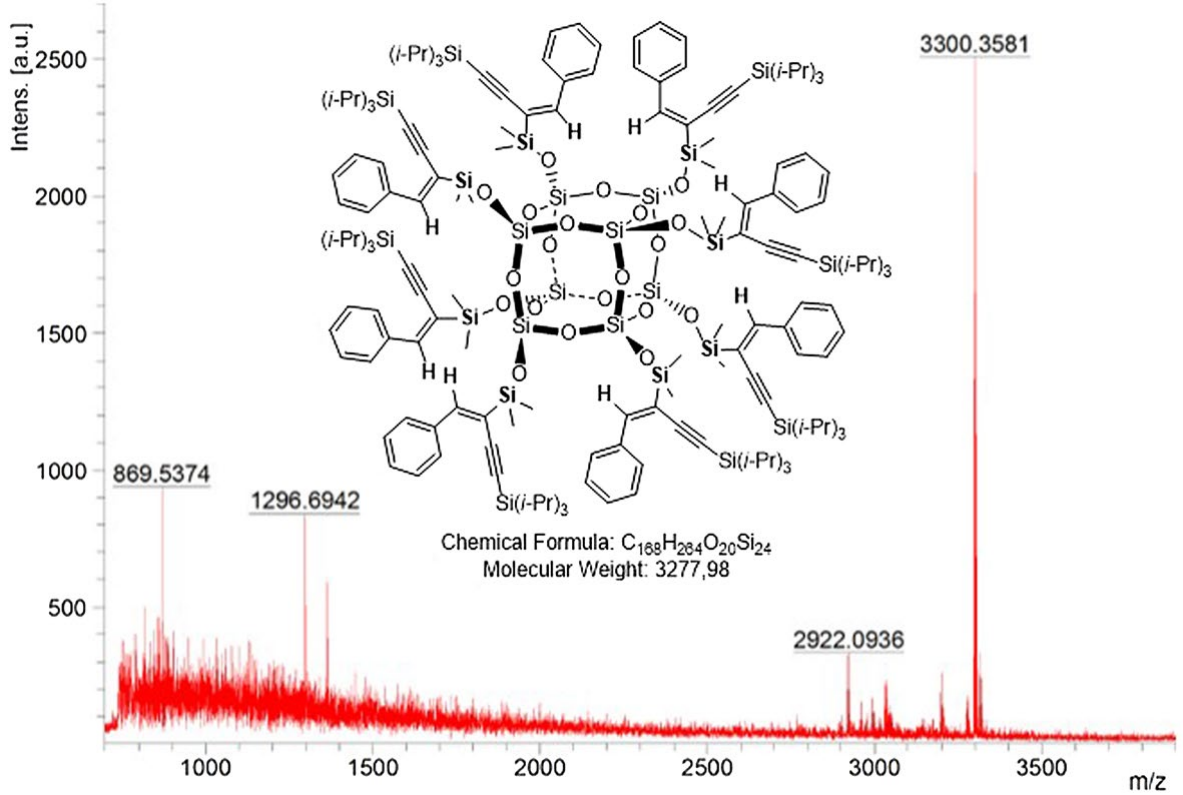
MALDI TOF MS spectrum of product **3f**.

All the products obtained were fully characterized by ^1^H, ^13^C, ^29^Si, 1D NOE, ^1^H–^13^C HSQC NMR, FT–IR, EA, UV–Vis, and MALDI TOF MS, which confirmed their structures. In the case of MALDI TOF MS, during ionization, both positive and negative ions can be formed (mainly H^+^, Na^+^, K^+^, a mixture of different adducts). For the octaspherosilicates with 3-en-1yl groups obtained here primarily ions stabilized by metal cations (usually sodium) were detected. As a result, the molecular weights observed in the spectrums were higher by the mass of sodium ([M + Na]^+^). The same results were found for the previously characterized octaspherosilicates with alkenyl substituents^92^. The representative MALDI TOF spectrum of compound **3f** is presented in Fig. 5. The MALDI TOF MS spectra for all products are included in ESI.

**Figure 6:**
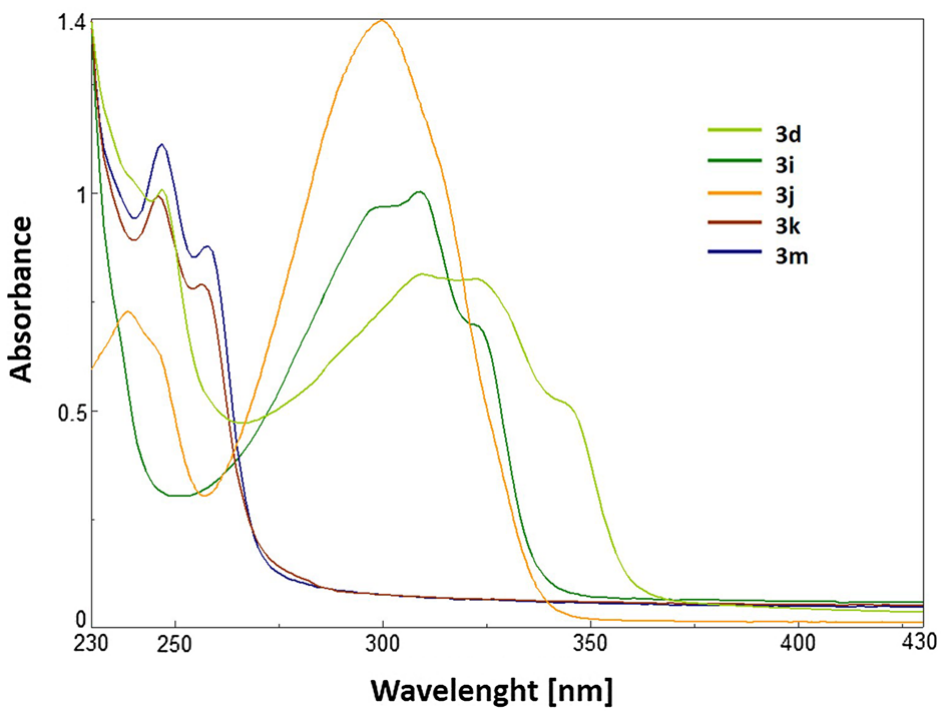
Normalized absorbance spectra of products **3d**, **3i**, **3j**, **3k,** and **3m** in the solution (DCM).

The results from UV–Vis measurements showed that the tested compounds (**3d**, **3i**, **3j**, **3k**, **3m**) absorb only in the UV range (200–400 nm), which is typical for molecules containing conjugated C–C bonds in their structures (Fig. 6). The spectra for products **3k** and **3m**, which possess only alkyl groups in their structures, were nearly the same. Product **3k** exhibited absorption peaks at 246 and 257 nm, while product **3m** showed absorption peaks at 246 and 258 nm. The presence of additional phenyl rings in the structures of **3d** and **3i** caused these compounds to absorb at slightly longer wavelengths. Specifically, compound **3d** exhibited absorption at 248 and 310 nm, while compound **3i** showed absorption at 309 nm. Product **3j**, with thienyl substituents, showed a similar absorption pattern to **3d**, with peaks observed at 238 and 299 nm.

**Figure 7:**
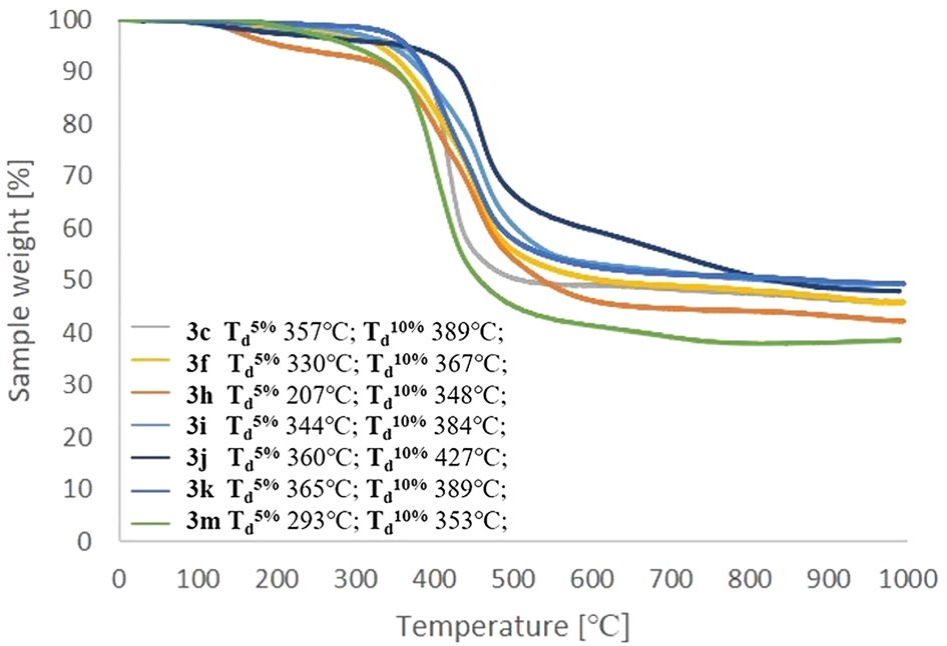
TGA curves for compounds **3c**, **3f.**, **3h-k**, and **3m**. The measurements were conducted under nitrogen (flow of 20 mL/min), from 29 to 995 °C at a heating rate of 10 °C/min.

The thermal properties of selected products were characterized using thermogravimetric analysis (TGA) performed in an inert atmosphere. The TGA results indicated that octaspherosilicates **3** are generally stable up to 300 ℃ (as shown in Fig. 7). The most thermally stable products were those obtained via the hydrosilylation of deca-1,3-diyn-1-yl-tri(*iso*propyl)silane (**2k**) (**3k**, T_d_^5%^ = 365 °C), tri(*iso*propyl)(thiophen-3-ylbuta-1,3-diyn-1-yl)silane (**2j**) (**3j**, T_d_^5%^ = 360 °C), and 2,2,7,7-tetramethylocta-3,5-diyne (**2c**) (**3c**, T_d_^5%^ = 357 °C). 10% weight loss for **3j** was observed at 427 °C and for **3c** and **3k** at 389 °C. On the other hand, compound **3h** was identified as the least thermally stable (T_d_^5%^ = 207 °C). The sample residue was in the range of 40 to 50%. The lowest residue was observed for compound **3m** and the highest for **3i** and **3k**.

## Conclusion

New synthetic protocols for the selective and efficient monohydrosilylation of symmetrically and non-symmetrically 1,4-disubstituted buta-1,3-diynes (**2a**–**m**) with octaspherosilicate (**1**) were successfully developed for the first time. The proposed approaches were based on the application of commercially available platinum catalysts and did not require the use of an inert atmosphere, or a special preparation or purification of reagents and catalysts. Moreover, the actual reaction times were measured, and in situ FT-IR and ^1^H NMR spectroscopies were used to determine the impact of both the buta-1,3-diyne structure and catalyst type on the hydrosilylation progress. It was found that hydrosilylation of less sterically crowded 1,3-diynes occurred faster. The 13 novel octaspherosilicates with 3-en-1-yl moieties (**3a**–**m**) were successfully synthesized with decent or high isolated yields (58–95%). The products were fully characterized by ^1^H, ^13^C, ^29^Si, 1D NOE, ^1^H-^13^C HSQC NMR, FT-IR, EA, UV–Vis, and MALDI TOF MS. The TGA proved the high thermal stability of the products, the most thermally stable product was **3j**, for which 5 and 10% weight loss were observed at as high a temperature as 360 and 427 °C, respectively.

Because of many applications of alkyl- and alkenyl-octaspherosilicates, the 3-en-1-yl derivatives obtained here become systems that can be tested in similar fields. Their greatest advantages are a new structure, as well as the possibility of further modification. The primary benefit of using 1,3-diynes in synthesis, compared to other unsaturated groups (like alkynes), is the ability to introduce both the unsaturated C=C bond (similar to the reaction with alkynes) and the C≡C bond into the product structure in a single reaction step. Furthermore, the C≡C bond can be subsequently modified in the next reaction step, for example, using hydrosilylation or hydroboration, which will lead again to the formation of new compounds with unique and as yet uncharacterized properties.

## Methods

Buta-1,3-diyne **2b** and **2c** were synthesized by Glaser homo-coupling of tri(*iso*propyl)silylacetylene and 3,3-dimethyl-1-butyne, respectively^99^. Buta-1,3-diynes **2e**, **2f–m** were synthesized by Cadiot-Chodkiewicz cross-coupling reaction^111^.

### General procedure for hydrosilylation of 1,3-diynes (2a-m) with octaspherosilicate 1 in the presence of Karstedt’s catalyst or Pt(PPh_3_)_4_

The reactions with 1,3-diynes **2c**–**e**, **2f**–**m** were monitored by in situ FT-IR spectroscopy. A solution of spherosilicate **1** (0.1 g, 0.098 mmol) and an appropriate buta-1,3-diyne (**2c**–**e**, **2f**–**m**) (0.784–3.136 mmol) in toluene was heated to 100 ℃ and stirred. Then, Karstedt’s catalyst or Pt(PPh_3_)_4_ was added in an amount that varied from 8 × 10^−3^ to 2 × 10^−1^ mol of Pt, depending on the experiment. The reaction was carried out until the full conversion of Si–H was detected by in situ FT-IR spectroscopy. For reactions with 1,3-diynes **2a** and **2d** that were not monitored by in situ FT-IR spectroscopy, Karstedt’s catalyst or Pt(PPh_3_)_4_ was added to the mixture of reagents in toluene, and then the system was heated to 100 ℃. The conversion of the reagents was determined by ^1^H NMR spectroscopy after 24, 48, 72, and 96 h (for NMR spectra for reactions with **2a** and **2d** see ESI, pages S37 and S44). After the reaction, the solvent was evaporated in a vacuum. The crude product was dissolved in hexane and purified on silica using flash column chromatography in hexane/ethyl acetate. Isolated products were characterized by ^1^H, ^13^C, ^29^Si, 1D NOE, ^1^H-^13^C HSQC NMR, FT-IR, and MALDI TOF analyses. The thermal properties of the selected products were characterized by TGA analysis.

For detailed data, please see the Electronic Supporting Information.

### Supplementary Information

Supplementary Information.

## Data Availability

All data generated or analyzed during this study are included in this published article and its supplementary information file.
