# Supplementary material for: Functionalization of octaspherosilicate (HSiMe2O)8Si8O12 with buta-1,3-diynes by hydrosilylation
Source: Sci Rep. 2023 Aug 31;13:14314. doi: 10.1038/s41598-023-41461-2 (PMC10471723; doi:10.1038/s41598-023-41461-2)
Supplement: Supplementary file 1 — Supplementary Information. [file 41598_2023_41461_MOESM1_ESM.pdf]

Functionalization of octaspherosilicate $(HSiMe_2O)_8Si_8O_{12}$ with buta-1,3-diynes by hydrosilylation

Kinga Stefanowska[a], Jakub Nagórny[a,b], Jakub Szyling[a], Adrian Franczyk[a,*]

[a]Center for Advanced Technology, Adam Mickiewicz University, Uniwersytetu Poznańskiego 10, 61-614 Poznań, Poland

[b]Faculty of Chemistry, Adam Mickiewicz University, Uniwersytetu Poznańskiego 8, 61-614 Poznań, Poland

*Corresponding author.

Email address: adrian.franczyk@amu.edu.pl

Table of Contents

1. Materials 3
2. Characterization of analytical methods 3
    2.1. NMR analysis 3
    2.2. Matrix-assisted ultraviolet laser desorption/ionization time-of-flight mass spectroscopy (MALDI-TOF-MS) 3
    2.3. FT-IR analysis 3
    2.4. *In situ* FT-IR analysis 3
    2.5. Thermogravimetric analysis (TGA) 4
    2.6. Elemental analyses 4
    2.7. UV-vis analysis 4
3. General information 4
    3.1. Synthesis of (bromoethynyl)tri(*iso*propyl)silane 4
    3.2. Synthesis of symmetrical 1,3-diynes (**2b**-**c**) 4
    3.3. Synthesis of unsymmetrical 1,3-diynes (**2e**-**m**) 5
4. Products purification 5
    4.1. 1,3-Diynes (**2c**-**m**) 5
    4.2. Compounds **3a**-**m** 5
5. Products characterization 5
6. NMR spectra 15
7. Literature 66

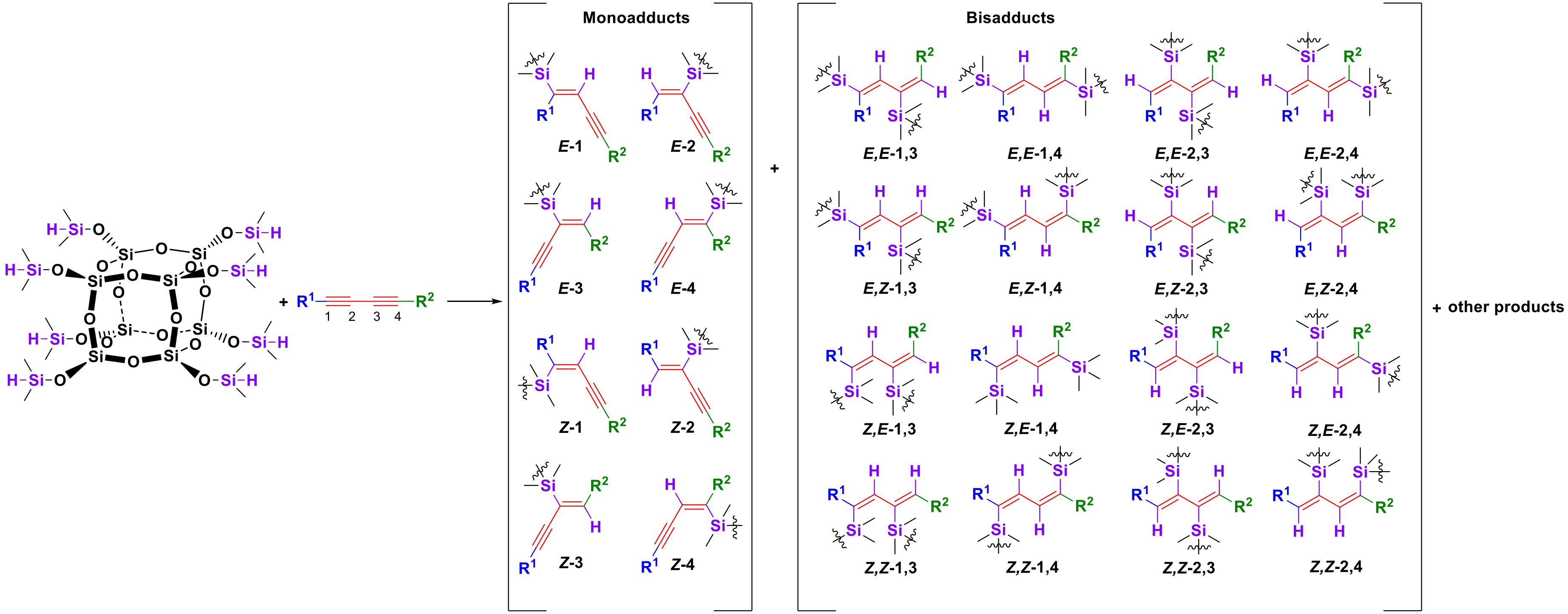

Figure S1. Scheme of possible mono- and bisadducts obtained *via* hydrosilylation of buta-1,3-diynes with octaspherosilicate **1.**

1. Materials

Octasilane POSS (Hybrid Plastics), 3,3-Dimethyl-1-butyne (98%, Sigma-Aldrich), tri(*iso*propyl)silylacetylene (97%, Sigma-Aldrich), 1-octyne (97%, Sigma-Aldrich), 1-bromo-4-ethynylbenzene (97%, Sigma-Aldrich), 4-ethynyl-α, α, α-trifluorotoluene (97%, Sigma-Aldrich), phenyl propargyl ether (≥90%, Sigma-Aldrich), 1-(*tert*-butyl)-4-ethynylbenzene (96%, Sigma-Aldrich), phenylacetylene (99%, Sigma-Aldrich), 1,4-diphenylbutadiyne (99%, Sigma-Aldrich), 1,4-bis(trimethylsilyl)butadiyne (98%, Sigma-Aldrich), N-bromosuccinimide (98%, Sigma-Aldrich), hydroxylamine hydrochloride (98%, abcr), *n*-butylamine (99%, Sigma-Aldrich), piperidine (99%, TCI), ammonium chloride (99%, Avantor Performance Materials Poland), silver nitrate (99%, Sigma-Aldrich), magnesium sulfate (anhydrous, 99%, Sigma-Aldrich), platinum(0)-1,3divinyl-1,1,3,3-tetramethyldisiloxane (Karstedt's catalyst, solution in xylene, Pt 2%, Sigma-Aldrich), chloroform-d (99.96 atom% D, Sigma-Aldrich). Petroleum ether (pure, Avantor Performance Materials Poland), methanol (pure, Avantor Performance Materials Poland), acetone (pure, Avantor Performance Materials Poland), dichloromethane (pure, Avantor Performance Materials Poland). Toluene and tetrahydrofuran were dried using standard procedures, deoxygenated, and stored over molecular sieves 4 Å under argon atmosphere. Argon (99,999%) was purchased from Linde. Silica gel (MN-Kieselgel 60, 0.04-0.063 mm (230-400 mesh ASTM; Sigma-Aldrich)) was used as received.

2. Characterization of analytical methods

2.1. NMR analysis

^{1}H, ^{13}C, ^{29}Si and ^{11}B NMR spectra were recorded at 25 °C on a Bruker Ultra Shield 300 MHz, Bruker Ascend 400 MHz, and Bruker Ascend 600 MHz NANOBAY spectrometers. $CDCl_3$ was used as a solvent and for internal deuterium lock. Chemical shifts are reported in ppm with reference to the residual portion solvent peak for ^{1}H and ^{13}C NMR, to TMS for ^{29}Si NMR. The multiplicities were reported as follows: singlet (s), doublet (d), triplet (t), and multiplet (m). To prove the regioselectivity of the process 2D heteronuclear single quantum correlation (HSQC) and selective gradient NOE experiments for selected products were performed.

2.2. Matrix-assisted ultraviolet laser desorption/ionization time-of-flight mass spectroscopy (MALDI-TOF-MS)

MALDI-TOF mass spectra were recorded on a UltrafleXtreme mass spectrometer (Bruker Daltonics), equipped with a SmartBeam II laser (355 nm) in 500-4000 m/z range. 2,5-Dihydroxybenzoic acid (DHB, Bruker Daltonics, Bremen, Germany) served as a matrix and was prepared in TA30 solvent (30:70 v/v acetonitrile: 0.1% TFA in water) at a concentration of 20 mg/mL. Studied samples were dissolved in dichloromethane (2 mg/mL) and then mixed in a ratio 1:1 v/v with matrix solution. Matrix/sample mixtures (1 μL) were spotted onto the MALDI target and dried in air. Mass spectra were measured in reflection mode. The data were analyzed using the software provided with the Ultraflex instrument - FlexAnalysis (version 3.4). Mass calibration (cubic calibration based on five to seven points) was performed using external standards (Peptide Calibration Standard).

2.3. FT-IR analysis

Fourier Transform-Infrared (FT-IR) spectra were recorded on a Bruker Tensor 27 Fourier transform spectrophotometer equipped with a SPECAC Golden Gate, diamond ATR unit, with ensuring a resolution of 2cm^{-1}.

2.4. *In situ* FT-IR analysis

In situ FT-IR measurements were performed on a Mettler-Toledo ReactIR 15 spectrometer equipped with 9.5 mm AgX DiComp (diamond) probe and a liquid nitrogen-cooled MCT detector. The spectra were taken with the resolution of 4 cm^{-1} collecting scans for each spectrum at 15 s intervals for 8 h and 2 minutes for the rest of reaction time. The reaction progress in the studied systems of parent compounds and catalysts was quantified by observing the rate of changes occurring with time in the

area of the band at 904 cm^{-1} originating from stretching vibrations of Si-H bond. A detailed description of the equipment used is available on the manufacturer's website[1].

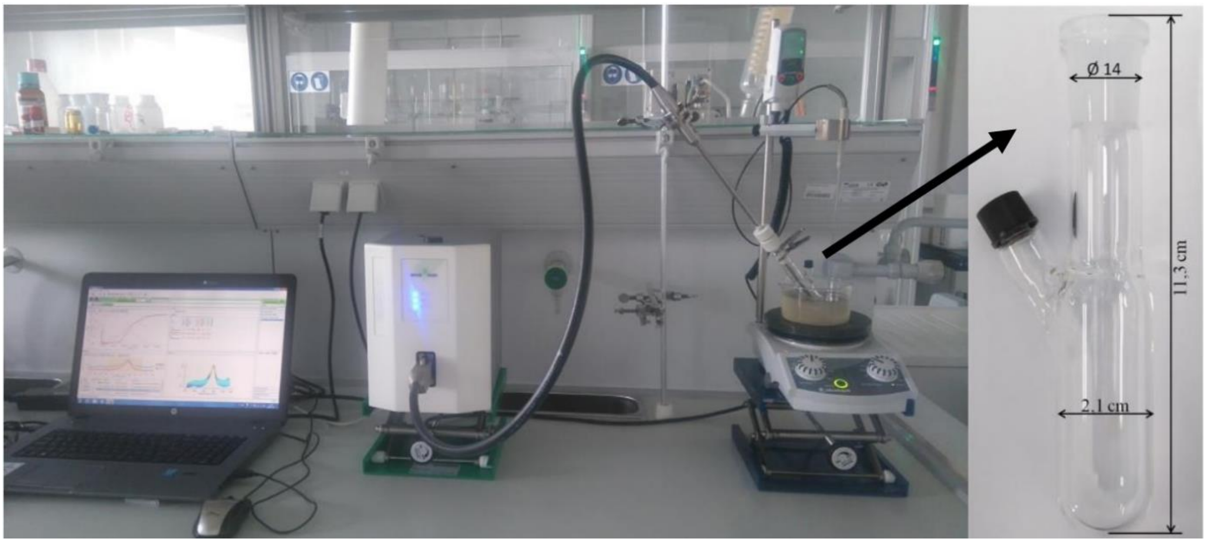

Figure S2. Hydrosilylation of buta-1,3-diynes with octaspherosilicate **1** monitored by *in situ* FT-IR spectroscopy.

2.5. Thermogravimetric analysis (TGA)

Thermogravimetric Analyses (TGA) were performed using a Netzsch TG 209 Libra thermal gravimetric analyzer. The measurements were conducted under nitrogen (flow of 20 mL/min), from 29 °C to 995 °C at the heating rate of 10 °C/min. The temperature of initial degradation ($T_{5\%}$) was taken as the onset temperature at which 5 wt% of mass loss occurs.

2.6. Elemental analyses

Elemental analyses were performed using a Vario EL III instrument.

2.7. UV-vis analysis

UV-vis spectra were recorded on a Jasco V-750 UV-visible spectrophotometer.

3. General information

3.1. Synthesis of (bromoethynyl)tri(*iso*propyl)silane

The title compound was prepared according to the literature with some modification[2]:

To a solution of tri(*iso*propyl)silylacetylene (10 mmol) in acetone (100 mL), *N*-bromosuccinimide (12 mmol) and silver nitrate (1 mmol) were successively added. The reaction mixture was stirred without light access at room temperature over 18 h before adding water (100 mL). The resulting mixture was extracted with hexanes (3 x 100 mL) and the combined organic layers were washed with brine (100 mL), dried over $MgSO_4$, filtered through a pad of silica and concentrated to give a colorless liquid.

Caution: (bromoethynyl)tri(*iso*propyl)silane is strong lachrymator. The isolation should be performed under the hood.

3.2. Synthesis of symmetrical 1,3-diynes (2b-c)

Symmetrical 1,3-diynes were prepared according to the following procedure:

The CuCl (0.1 mmol) was placed in a round bottom bulb equipped with a condenser and magnetic stirring bar. Subsequently, toluene (10 mL), piperidine (0.15 mmol), and alkyne (5 mmol) were placed in the reaction vessel. The reaction was performed at 80 °C for 18 hours with a constant gentle flow of compressed air delivered from the top of the condenser. Afterwards, the reaction mixture was cooled and all volatiles were removed under vacuum. The crude residue was dissolved in hexanes (with a

small amount of dichloromethane if necessary) and purified. The synthesis of **2a** was performed in a Rotaflo®-type Schlenk vessel due to the low boiling point of the initial alkyne.

3.3. Synthesis of unsymmetrical 1,3-diynes (2e-m)

The unsymmetrical 1,3-diynes were prepared according to the literature with some modifications[3]: CuCl was dissolved in a 2:3 mixture by volume of *n*-BuNH$_2$:H$_2$O (5 mL/mmol alkyne) and the solution was cooled to 0 °C in an ice bath. Hydroxylamine hydrochloride was slowly added until trace amounts of copper(II) were reduced and the color of the solution changed from blue to colorless. The alkyne bromide and alkyne were dissolved in dichloromethane (5 mL/mmol alkyne), cooled down to 0 °C, and this solution was added to the reaction flask at once. The biphasic mixture was vigorously stirred overnight under an argon atmosphere. Subsequently, the organic layer was removed and washed with portions of saturated aq. NH_4Cl until these portions no longer took on a blue color. The organic layer was dried ($MgSO_4$) and concentrated by rotary evaporation. The crude residue was dissolved in hexanes and purified.

4. Products purification

4.1. 1,3-Diynes (2c-m)

The UV-absorbing products (1,3-diynes) were purified on silica by flash chromatography (Biotage IsoleraOne chromatograph) with UV detector (λ_1 = 255 nm, λ_2= 280 nm). Purification details: cartridge 10 g, flow rate: 12 mL/min, length: 10 CV (CV = column volume), phase: *n*-hexane/dichloromethane (step 1: *n*-hexane 100% by 4 CV, step 2: gradient 10%/CV by 4 CV, step 3: *n*-hexane 50% by 2 CV). The non-aromatic products (1,3-diynes) were purified on silica using standard column chromatography using *n*-hexane/dichloromethane (95/5–7/3) as eluents. Products were characterized by GC-MS, ^{1}H, ^{13}C, ^{29}Si NMR, FT-IR analyses.

4.2. Compounds 3a-m

The products were purified on silica by flash chromatography (Biotage IsoleraOne chromatograph) with UV detector (λ_1 = 255 nm, λ_2= 280 nm). Purification details: cartridge 10 g, flow rate: 8 mL/min, length: 10 CV (CV = column volume), phase: hexane/ethyl acetate (step 1: hexane 100% by 4 CV, step 2: gradient 10%/CV by 10 CV, step 3: hexane 50% by 2 CV). Product **3d** was isolated by precipitation in *n*-hexane. Products were characterized by ^{1}H, ^{13}C, ^{29}Si NMR, FT-IR, MALDI TOF analyses. The thermal properties selected products were characterized by the TGA (performed under an inert atmosphere).

5. Products characterization

1,4-Bis(trimethylsilyl)buta-1,3-diyne (2a)

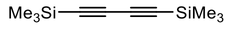

Chemical Formula: $C_{10}H_{18}Si_2$
Molecular Weight: 194,42

^{1}H NMR (400 MHz, $CDCl_3$, δ, ppm): 0.16 (s, 18H, C<u>H</u>$_3$). **^{13}C NMR** (101 MHz, $CDCl_3$, δ, ppm): 0.12 (<u>C</u>H$_3$), 113.95 (C≡C). **^{29}Si NMR** (79 MHz, $CDCl_3$ δ, ppm): -19.23. Pale yellow solid.

1,4-Bis(tri(*iso*propyl)silyl)buta-1,3-diyne (2b)

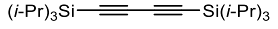

Chemical Formula: $C_{22}H_{42}Si_2$
Molecular Weight: 362,75

^{1}H NMR (300 MHz, $CDCl_3$, δ, ppm): 1.09 (s, 42H, Si(C<u>H</u>(C<u>H</u>$_3$)$_2$). **^{13}C NMR** (101 MHz, $CDCl_3$, δ, ppm): 11.47 (<u>C</u>(CH$_3$)$_3$), 18.72 (C(<u>C</u>H$_3$)$_3$), 81.73 (C≡C), 90.34 (C≡C). **^{29}Si NMR** (79 MHz, $CDCl_3$ δ, ppm): -0.86. **MS** (EI, m/z): 362(M$^+$, 10), 319(100), 291(23), 277(26), 263(15), 249(29), 235(15), 207(11). 193(13), 179(11),

165(14), 151(12), 137(16), 82(31), 59(17). **FT-IR** (cm^{-1}): 3055, 3031, 2112, 1587, 1477, 1436, 825, 755, 719, 691. Isolated yield = 81%. White solid. Analytical data are in agreement with the literature.[4]

2,2,7,7-Tetramethylocta-3,5-diyne (2c)

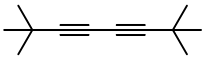

Chemical Formula: $C_{12}H_{18}$
Molecular Weight: 162,28

^{1}H NMR (300 MHz, $CDCl_3$, δ, ppm): 1.23 (s, 18H, $(C\underline{H}_3)_3$). **^{13}C NMR** (101 MHz, $CDCl_3$, δ, ppm): 28.12 ($\underline{C}(CH_3)_3$), 30.76 ($C(\underline{C}H_3)_3$), 63.81 (C≡C), 86.44 (C≡C). **MS** (EI, m/z): 162(M^+, 74), 147(50), 132(10), 119(100), 105(92), 91(89), 77(36), 55(24). **FT-IR** (cm^{-1}): 2968, 2864, 2141, 1465, 1361, 1239, 1198, 677. Isolated yield = 87%. White solid. Analytical data are in agreement with the literature.[5]

1,4-Diphenylbuta-1,3-diyne (2d)

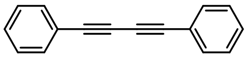

Chemical Formula: $C_{16}H_{10}$
Molecular Weight: 202,26

^{1}H NMR (300 MHz, $CDCl_3$, δ, ppm): 7.35-7.56 (m, 10H, $C_6\underline{H}_5$). **^{13}C NMR** (101 MHz, $CDCl_3$, δ, ppm): 74.07 (C≡C), 81.70 (C≡C), 121.91, 128.57, 129.34, 132.62 ($\underline{C}_6H_5$). White solid.

Trimethyl(phenylbuta-1,3-diyn-1-yl)silane (2e)

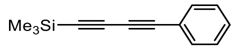

Chemical Formula: $C_{13}H_{14}Si$
Molecular Weight: 198,34

^{1}H NMR (400 MHz, $CDCl_3$, δ, ppm): 0.24 (s, 9H, $C\underline{H}_3$), 7.26-7.51 (m, 5H, $C_6\underline{H}_5$). **^{13}C NMR** (101 MHz, $CDCl_3$, δ, ppm): -0.24 ($\underline{C}H_3$), 74.28 (C≡C), 76.84 (C≡C), 76.88 (C≡C), 77.48 (C≡C), 87.96, 90.78, 121.53, 128.56, 129.47, 132.82 (C_6H_5). **^{29}Si NMR** (79 MHz, $CDCl_3$ δ, ppm): -16.10. Isolated yield = 66%. Pale yellow oil. Analytical data are in agreement with the literature.[6]

(Phenylbuta-1,3-diyn-1-yl)tri*(iso*propyl)silane (2f)

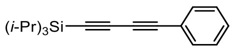

Chemical Formula: $C_{19}H_{26}Si$
Molecular Weight: 282,50

^{1}H NMR (300 MHz, $CDCl_3$, δ, ppm): 1.12 (s, 21H, $Si(C\underline{H}(C\underline{H}_3)_2)$, 7.55 – 7.27 (m, 5H, Ph). **^{13}C NMR** (75 MHz, $CDCl_3$, δ, ppm): 11.45 ($Si(\underline{C}H(CH_3)_2)_3$), 18.72 ($Si(CH(\underline{C}H_3)_2)_3$), 74.80 (C≡C), 75.69 (C≡C), 88.03 (C≡C), 89.63 (C≡C), 121.68, 128.54, 129.36, 132.84 ($\underline{C}_6H_5$). **^{29}Si NMR** (79 MHz, $CDCl_3$ δ, ppm): -0.65. **MS** (EI, m/z): 282(M^+, 8), 239(98), 211(44), 197(40), 183(54), 169(100), 159(21), 153(27), 91(20) 59(10). **FT-IR** (cm^{-1}): 2942, 2890, 2865, 2204, 2101, 1488, 1461, 1070, 1018, 995, 881, 752, 729, 675, 602. Isolated yield = 75%. Colorless oil. Analytical data are in agreement with the literature.[7]

((4-Bromophenyl)buta-1,3-diyn-1-yl)tri*(iso*propyl)silane (2g)

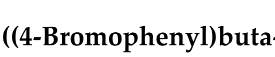

Chemical Formula: $C_{19}H_{25}BrSi$
Molecular Weight: 361,40

^{1}**H NMR** (300 MHz, $CDCl_3$, δ, ppm): 1.11 (s, 21H, $Si(C\underline{H}(C\underline{H}_3)_2)_3$), 7.39 – 7.33 (m, 2H, Ph), 7.49 – 7.43 (m, 2H, Ph). ^{13}**C NMR** (75 MHz, $CDCl_3$, δ, ppm): 11.43 ($Si(\underline{C}H(CH_3)_2)_3$), 18.71 ($Si(CH(\underline{C}H_3)_2)_3$), 74.52 (C≡C), 75.90 (C≡C), 88.97 (C≡C), 89.38 (C≡C), 120.67, 123.86, 131.89, 134.16 ($\underline{C}_6H_4$). ^{29}**Si NMR** (79 MHz, $CDCl_3$ δ, ppm): -0.46. **MS** (EI, m/z): 362(M^+, 10), 360(M^+-2, 12), 319(100), 317(98), 291(33), 289(33), 277(28), 275(26), 249(61), 247(61), 153(16), 151(17), 109(10), 59(8). **FT-IR** (cm^{-1}): 2942, 2890, 2864, 2102, 1485, 1462, 1070, 1009, 881, 820, 745, 676, 613. Isolated yield = 74%. White solid. Analytical data are in agreement with the literature.[8]

Tri(*iso*propyl)((4-(trifluoromethyl)phenyl)buta-1,3-diyn-1-yl)silane (2h)

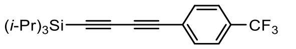

Chemical Formula: $C_{20}H_{25}F_3Si$
Molecular Weight: 350,50

^{1}**H NMR** (300 MHz, $CDCl_3$, δ, ppm): 1.12 (s, 21H, $Si(C\underline{H}(C\underline{H}_3)_2)$, 7.68 – 7.50 (m, 4H, Ph). ^{13}**C NMR** (101 MHz, $CDCl_3$, δ, ppm): 11.43 ($Si(\underline{C}H(CH_3)_2)_3$), 18.71 ($Si(CH(\underline{C}H_3)_2)_3$), 73.98 (C≡C), 89.07 (C≡C), 89.94 (C≡C), 122.54 ($\underline{C}_6H_4$), 125.50 (q, $\underline{C}F_3$, $J^3{}_{C\text{-}F}$ = 3.8 Hz), 130.77, 131.09, 133.05 ($\underline{C}_6H_4$). ^{29}**Si NMR** (79 MHz, $CDCl_3$ δ, ppm): -0.28. **MS** (EI, m/z): 350(M^+, 5), 307(100), 279(38), 265(26), 251(55), 237(82), 197(12), 175(17), 151(7), 137(8), 125(6). **FT-IR** (cm^{-1}): 2944, 2892, 2867, 2104, 1614, 1462, 1318, 1168, 1129, 1105, 1067, 1015, 881, 839, 676, 659, 613, 595. Isolated yield = 81%. Colorless oil. Analytical data are in agreement with the literature.[8]

((4-(*Tert*-butyl)phenyl)buta-1,3-diyn-1-yl)tri(*iso*propyl)silane (2i)

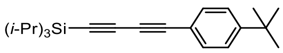

Chemical Formula: $C_{23}H_{34}Si$
Molecular Weight: 338,61

^{1}**H NMR** (600 MHz, $CDCl_3$, δ, ppm): 1.12 (s, 21H, $CH(C\underline{H}_3)_2$, $C\underline{H}$), 1.31 (s, 9H, $C(C\underline{H}_3)_3$), 7.33, 7.34 (d, 2H, $C_6\underline{H}_4C(CH_3)_3$, $J_{(H,H)}$= 8.46 Hz), 7.44, 7.45 (d, 2H, $C_6\underline{H}_4C(CH_3)_3$, $J_{(H,H)}$= 8.45 Hz). ^{13}**C NMR** (151 MHz, $CDCl_3$, δ, ppm): 11.48 ($\underline{C}H(CH_3)_2$), ,18.74 ($CH(\underline{C}H_3)_2$), 31.24 ($C(\underline{C}H_3)_3$), 35.05 ($\underline{C}(CH_3)_3$), 74.21 (C≡C), 76.01 (C≡C), 87.44 (C≡C), 89.88 (C≡C), 118.56, 125.59, 132.63, 152.81 ($\underline{C}_6H_4$). ^{29}**Si NMR** (79 MHz, $CDCl_3$ δ, ppm): -0.77. **MS** (EI, m/z): 338(M^+, 22), 296(26), 295(100), 267(38), 253(40), 239(33), 226(15), 225(78), 112(18), 98(18), 57(16). **FT-IR** (cm^{-1}): 2943, 2865, 2202, 2099,1460, 1364,1109, 1013, 995, 882, 836, 755, 662, 561. Isolated yield = 71%. Pale yellow solid.

Tri(*iso*propyl)(thiophen-3-ylbuta-1,3-diyn-1-yl)silane (2j)

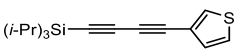

Chemical Formula: $C_{17}H_{24}SSi$
Molecular Weight: 288,52

^{1}**H NMR** (300 MHz, $CDCl_3$, δ, ppm): 1.10 (s, 21H, $CH(C\underline{H}_3)_2$, $C\underline{H}$), 7.13, 7.15 (d-d, 1H, $C_4\underline{H}_3S$, $J_{(H,H)}$= 5.02, 1.19 Hz), 7.23, 7.24, 7.25, 7.26 (d-d, 1H, $C_4\underline{H}_3S$, $J_{(H,H)}$= 5.03, 2.97 Hz), 7.56, 7.57 (d-d, 1H, $C_4\underline{H}_3S$, $J_{(H,H)}$= 3.00, 1.18 Hz). ^{13}**C NMR** (151 MHz, $CDCl_3$, δ, ppm): 11.45 ($\underline{C}H(CH_3)_2$), 18.72 ($CH(\underline{C}H_3)_2$), 70.92 (C≡C), 74.47 (C≡C), 87.90 (C≡C), 89.60 (C≡C), 120.78, 125.68, 130.39, 131.74 ($\underline{C}_4H_3S$). ^{29}**Si NMR** (79 MHz, $CDCl_3$ δ, ppm): -0.84. **MS** (EI, m/z): 288(M^+, 18), 246(20), 245(26), 217(44), 203(41), 189(49), 175(94), 159(19), 95(24). **FT-IR** (cm^{-1}): 3110, 2942, 2890, 2864, 2202, 2101, 1461, 1414, 1383, 1356, 1255, 1190, 1074, 1018, 996, 895, 882, 863, 816, 778, 676, 661, 621, 603, 589. Isolated yield = 84%. Pale yellow oil. Analytical data are in agreement with the literature.[9]

Deca-1,3-diyn-1-yltri(*iso*propyl)silane (2k)

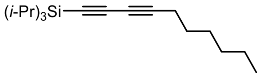

Chemical Formula: $C_{19}H_{34}Si$
Molecular Weight: 290,57

[1]**H NMR** (300 MHz, $CDCl_3$, δ, ppm): 0.89 (t, $J_{H\text{-}H}$ = 6.8 Hz, 3H, $CH_2C\underline{H}_3$), 1.08 (s, 21H, $Si(C\underline{H}(C\underline{H}_3)_2)_3$), 1.59 – 1.18 (m, 8H), 2.28 (d, $J_{H\text{-}H}$ = 7.0 Hz, 2H, $\equiv CC\underline{H}_2$). [13]**C NMR** (75 MHz, $CDCl_3$, δ, ppm): 11.44 ($Si(\underline{C}H(CH_3)_2)_3$), 14.18 ($\underline{C}H_3$), 18.70 ($Si(CH(\underline{C}H_3)_2)_3$), 19.44 ($\underline{C}H_2(CH_2)_4CH_3$), 22.64 ($\underline{C}H_2CH_3$), 28.28 ($\underline{C}H_2(CH_2)_3CH_3$), 28.75 ($\underline{C}H_2(CH_2)_2CH_3$), 31.43 ($\underline{C}H_2CH_2CH_3$), 65.94 (C≡C), 79.12 (C≡C), 80.09 (C≡C), 90.25 (C≡C). [29]**Si NMR** (79 MHz, $CDCl_3$ δ, ppm): -1.14. **MS** (EI, m/z): 290(M^+, 3), 247(100), 219(38), 205(33), 191(28), 177(52), 163(5), 149(9), 137(11), 109(13), 95(10), 83(15), 59(20). **FT-IR** (cm^{-1}): 2941, 2865, 2223, 2104, 1462, 1181, 995, 881, 675. Isolated yield = 82%. Pale yellow oil. Analytical data are in agreement with the literature.[10]

(5-Phenoxypenta-1,3-diyn-1-yl)tri(*iso*propyl)silane (2l)

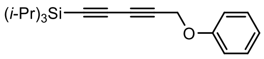

Chemical Formula: $C_{20}H_{28}OSi$
Molecular Weight: 312,53

[1]**H NMR** (300 MHz, $CDCl_3$, δ, ppm): 1.11 (s, 21H, $Si(C\underline{H}(C\underline{H}_3)_2)$, 4.80 (s, 2H, $\equiv CC\underline{H}_2OPh$), 7.08 – 6.96 (m, 3H, Ph), 7.42 – 7.31 (m, 2H, Ph). [13]**C NMR** (75 MHz, $CDCl_3$, δ, ppm): 11.35 ($Si(\underline{C}H(CH_3)_2)_3$), 18.64 ($Si(CH(\underline{C}H_3)_2)_3$),56.42 ($\underline{C}H_2O$), 71.48 (C≡C),72.49 (C≡C), 85.52 (C≡C), 88.84 (C≡C), 114.97, 121.82, 125.27, 129.70, 157.69 ($\underline{C}_6H_5$). [29]**Si NMR** (79 MHz, $CDCl_3$ δ, ppm): -0.45. **MS** (EI, m/z): 312(M^+, 26), 269(100), 241(50), 225(29), 213(24), 199(38), 185(20), 173(30), 151(81), 137(32), 121(22), 106(34), 92(19), 59(25). **FT-IR** (cm^{-1}): 2943, 2891, 2865, 2225, 2106, 1598, 1588, 1494, 1461, 1211, 1172, 1032, 1015, 994, 881, 801, 750, 676. Isolated yield = 69%. Yellow oil. Analytical data are in agreement with the literature.[11]

3-Methyl-7-(tri(*iso*propyl)silyl)hepta-4,6-diyn-3-ol (2m)

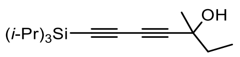

Chemical Formula: $C_{17}H_{30}OSi$
Molecular Weight: 278,51

[1]**H NMR** (400 MHz, $CDCl_3$, δ, ppm): 1.03, 1.05, 1.07 (t, 3H, $CH_2C\underline{H}_3$, $J_{(H,H)}$= 7.46 Hz), 1.08 (s, 21H, $C\underline{H}_3$), 1.69-1.75 (qd, 2H, $C\underline{H}_2CH_3$, $J_{(H,H)}$= 7.46, 2.66 Hz), 1.94 (s, 1H, $O\underline{H}$). [13]**C NMR** (101 MHz, $CDCl_3$, δ, ppm): 9.05 ($CH_2\underline{C}H_3$), 11.39 ($\underline{C}H(CH_3)_2$), 18.67 ($CH(\underline{C}H_3)_2$), 29.07 ($\underline{C}H_3$), 36.50 ($\underline{C}H_2CH_3$), 68.91 (C≡C), 69.32 ($\underline{C}(CH_3)(OH)(C_2H_5)$, 79.99 (C≡C), 84.66 (C≡C), 89.05 (C≡C). [29]**Si NMR** (79 MHz, $CDCl_3$ δ, ppm): -0.73. **MS** (EI, m/z): 235(M^+-43, 52), 207(20), 165(16), 109(21), 107(17), 93(19), 85(20), 75(100), 77(20), 61(33). **FT-IR** (cm^{-1}): 3287, 2942, 2865, 2102, 1461, 1382, 1367, 1326, 1132, 1103, 1074, 1052, 1012, 994, 915, 883, 861, 673, 580, 520, 483, 453. Isolated yield = 69%. Pale yellow solid.

Compound 1

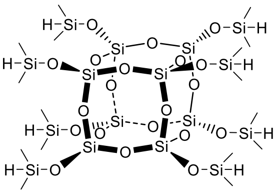

Chemical Formula: $C_{16}H_{56}O_{20}Si_{16}$
Molecular Weight: 1017,96

^{1}**H NMR** ($CDCl_3$, 300 MHz, δ, ppm): 0.26 (s, 48H, $OSi(C\underline{H}_3)_2$), 4.74 (m, 8H, $Si\underline{H}$). ^{13}**C NMR** ($CDCl_3$, 75 MHz, δ, ppm): 0.21 ($OSi(\underline{C}H_3)_2$). ^{29}**Si NMR** ($CDCl_3$, 79 MHz, δ, ppm): - 108.66 (SiO_4, Q^4), -1.44 ($OSi(CH_3)_2$, Q^1). **FT IR (cm^{-1}):** 2965, 2140, 1254, 1050, 888, 833, 540. Isolated yield = 91%. White solid.

Compound 3a

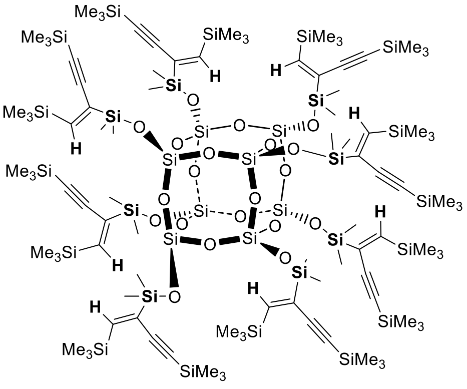

Chemical Formula: $C_{96}H_{200}O_{20}Si_{32}$
Molecular Weight: 2573,36

^{1}**H NMR** ($CDCl_3$, 300 MHz, δ, ppm): 0.16 (s, 72H, $Si(C\underline{H}_3)_3$), 0.17 (s, 72H, $Si(C\underline{H}_3)_3$), 0.27 (s, 48H, $Si(C\underline{H}_3)_2$), 6.77 (s, 8H, $=C\underline{H}$). ^{13}**C NMR** ($CDCl_3$, 75 MHz, δ, ppm): -0.79 ($OSi\underline{C}H_3$), 0.00 ($Si\underline{C}H_3$), 103.98 ($\underline{C}{\equiv}CSiMe_3$), 106.59 ($C{\equiv}\underline{C}SiMe_3$), 142.60 ($=\underline{C}C{\equiv}CSiMe_3$), 156.92 ($=\underline{C}(H)SiMe_3$). ^{29}**Si NMR** ($CDCl_3$, 80 MHz, δ, ppm): -108.89 (SiO_4, Q^4), -18.85 ($Si(CH_3)_3$), -8.24 ($Si(CH_3)_3$), -2.00 ($OSi(CH_3)_2$, Q^1). **FT-IR** (cm^{-1}): 2958, 2899, 2133, 1890, 1405, 1248, 1077, 827, 785, 758, 695, 629, 555, 500. Elemental Anal. for $C_{96}H_{200}O_{20}Si_{32}$ (%): calcd.: C, 44.81; H, 7.83; found: C, 44.75; H, 7.79. Isolated yield = 80%, pale yellow oil.

Compound 3b

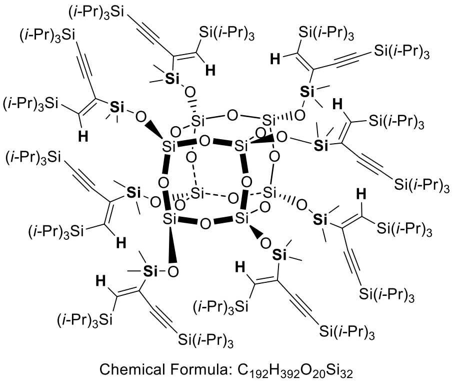

Chemical Formula: $C_{192}H_{392}O_{20}Si_{32}$
Molecular Weight: 3919,95

^{1}**H NMR** ($CDCl_3$, 300 MHz, δ, ppm): 0.27 (s, 48H, $Si(C\underline{H}_3)_2$), 1.06 (m, 312H, $C\underline{H}(CH_3)_2$, $CH(C\underline{H}_3)_2$), 1.34-1.44 (m, 24H, $C\underline{H}(CH_3)_2$), 6.71 (s, 8H, $=C\underline{H}$). ^{13}**C NMR** ($CDCl_3$, 151 MHz, δ, ppm): -0.47 ($OSi\underline{C}H_3$), 11.66, 11.86 ($Si(\underline{C}H(CH_3)_2)_3$), 18.70, 19.11 ($Si(CH(\underline{C}H_3)_2)_3$), 101.06 ($\underline{C}{\equiv}CSi(i\text{-}Pr)_3$)), 108.66 ($C{\equiv}\underline{C}Si(i\text{-}Pr)_3$)), 144.52

(=$\underline{C}$C≡CSi(i-Pr)$_3$)), 151.54 (=$\underline{C}$(H)Si(i-Pr)$_3$). **^{29}Si NMR** ($CDCl_3$, 119 MHz, δ, ppm): -108.33 (SiO_4, Q^4), -3.45, -2.01 (Si(i-Pr)$_3$), 0.23 (OSi(CH_3)$_2$, Q^1). **FT IR (cm^{-1}):** 2940, 2889, 2864, 2066, 1864, 1461, 1382, 1366, 1086, 994, 881, 830, 720, 663, 640, 527, 496, 457. **MALDI TOF MS** - (m/z) ([M+Na], (%)): 3942.2. Elemental Anal. for $C_{192}H_{392}O_{20}Si_{32}$ (%): calcd.: C, 58.83; H, 10.08; found: C, 58.74; H, 10.01. Isolated yield = 85%. Colorless oil.

Compound 3c

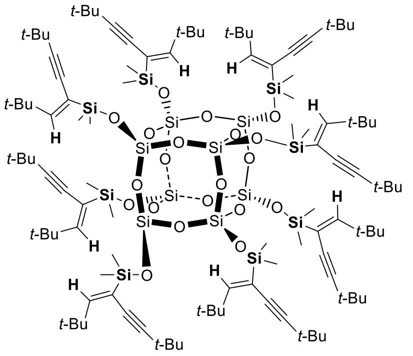

Chemical Formula: $C_{112}H_{200}O_{20}Si_{16}$
Molecular Weight: 2316,17

^{1}H NMR ($CDCl_3$, 300 MHz, δ, ppm): 0.24 (s, 48H, Si(C$\underline{H}_3$)$_2$), 1.18 (s, 72H, C(C$\underline{H}_3$)$_3$), 1.23 (s, 72H, C(C$\underline{H}_3$)$_3$), 6.10 (s, 8H, =C$\underline{H}$). **^{13}C NMR** ($CDCl_3$, 101 MHz, δ, ppm): -0.47 (OSi$\underline{C}H_3$), 28.58 (≡C$\underline{C}$(CH_3)$_3$), 29.86 (=C(H)C($\underline{C}H_3$)$_3$), 31.04 (≡CC($\underline{C}H_3$)$_3$), 35.65 (=C(H)$\underline{C}$(CH_3)$_3$), 77.96 (≡$\underline{C}$C(CH_3)$_3$), 109.02 ($\underline{C}$≡CC(CH_3)$_3$), 120.16 (=$\underline{C}$C≡CC(CH_3)$_3$), 158.54 (=$\underline{C}$(H)C(CH_3)$_3$). **^{29}Si NMR** ($CDCl_3$, 79 MHz, δ, ppm): -109.00 (SiO_4, Q^4), -0.38 (OSi(CH_3)$_2$, Q^1). **FT IR (cm^{-1}):** 2964, 2902, 2866, 1460, 1361, 1252, 1069, 900, 819, 785, 557, 482, 435. **MALDI TOF MS** - (m/z) ([M+Na], (%)): 2339.1. Elemental Anal. for $C_{112}H_{200}O_{20}Si_{16}$ (%): calcd.: C, 58.08; H, 8.70; found: C, 58.01; H, 8.65. Isolated yield = 90%. White solid.

Compound 3d

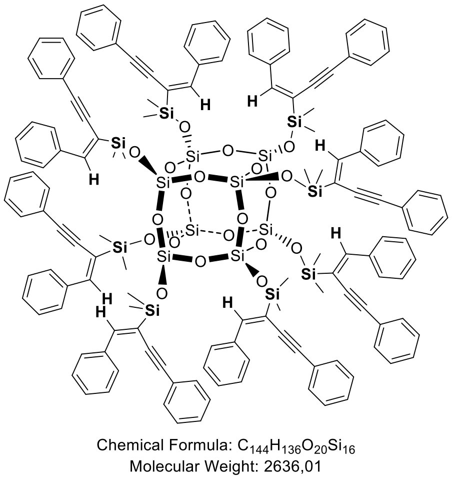

Chemical Formula: $C_{144}H_{136}O_{20}Si_{16}$
Molecular Weight: 2636,01

^{1}H NMR ($CDCl_3$, 300 MHz, δ, ppm): 0.00, 0.27 (s, 48H, (C$\underline{H}_3$)$_2$Si), 6.94 – 7.84 (m, 88H, =C$\underline{H}$, $C_6\underline{H}_5$). **^{13}C NMR** ($CDCl_3$, 75 MHz, δ, ppm): -0.48 (OSi$\underline{C}H_3$), 89.73 ($\underline{C}$≡CC_6H_5), 101.02 (C≡$\underline{C}C_6H_5$), 124.33 (=$\underline{C}$C≡CC_6H_5), 121.14, 128.30, 128.48, 129.75, 128.18, 131.43, 132.19, 132.32, 137.57 ($\underline{C}_6H_5$), 145.20 (=$\underline{C}$(H)C_6H_5). **^{29}Si NMR** ($CDCl_3$, 79 MHz, δ, ppm): -108.80 (SiO_4, Q^4), 1.30 (OSi(CH_3)$_2$, Q^1). **FT IR (cm^{-1}):** 2957, 2923, 2853, 1597, 1489, 1443, 1253, 1069, 1019, 821, 788, 752, 687, 549, 525, 464. **MALDI TOF MS** - (m/z) ([M+Na], (%)): 2658.57. Elemental Anal. for $C_{144}H_{136}O_{20}Si_{16}$ (%): calcd.: C, 65.61; H, 5.20; found: C, 65.67; H, 5.24. Isolated yield = 68%. White solid.

Compound 3e

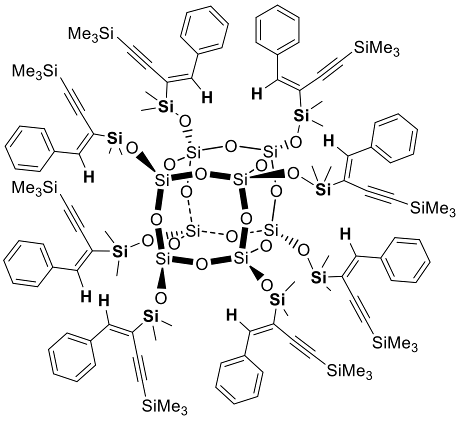

Chemical Formula: $C_{120}H_{168}O_{20}Si_{24}$
Molecular Weight: 2604,68

^{1}H NMR ($CDCl_3$, 300 MHz, δ, ppm): 0.20 (s, 72H, $Si(C\underline{H}_3)_3$), 0.32 (s, 48H, $Si(C\underline{H}_3)_2$), 7.00 (s, 8H, $=C\underline{H}$), 7.21-7.93 (m, 40H, $C_6\underline{H}_5$). **^{13}C NMR** ($CDCl_3$, 101 MHz, δ, ppm): -0.64 ($OSi\underline{C}H_3$), 0.04 ($Si\underline{C}H_3$), 105.41 ($\underline{C}{\equiv}CSiMe_3$), 107.23 ($C{\equiv}\underline{C}SiMe_3$), 121.35, 128.15, 128.44, 128.85, 129.22, 131.29 ($\underline{C}_6H_5$), 137.42 ($=\underline{C}C{\equiv}CSiMe_3$), 146.32 ($=\underline{C}(H)C_6H_5$). **^{29}Si NMR** ($CDCl_3$, 79 MHz, δ, ppm): -108.92 (SiO_4, Q^4), -18.41 ($Si(CH_3)_3$), 1.09 ($OSi(CH_3)_2$, Q^1). **FT-IR** (cm^{-1}): 2959, 2110, 1560, 1494, 1447, 1250, 1075, 837, 861, 788, 754, 689, 635, 601, 576, 552, 513, 484. Elemental Anal. for $C_{120}H_{168}O_{20}Si_{24}$ (%): calcd.: C, 55.34; H, 6.50; found: C, 55.39; H, 6.55. Isolated yield = 58%. Pale yellow solid.

Compound 3f

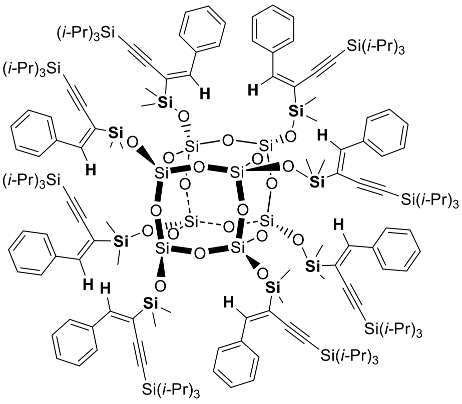

Chemical Formula: $C_{168}H_{264}O_{20}Si_{24}$
Molecular Weight: 3277,98

^{1}H NMR ($CDCl_3$, 300 MHz, δ, ppm): 0.36 (s, 48H, $Si(C\underline{H}_3)_2$), 1.11 (s, 168H, $C\underline{H}(CH_3)_2$, $CH(C\underline{H}_3)_2$), 7.06 (s, 8H, $=C\underline{H}$), 7.18-8.01 (m, 40H, $C_6\underline{H}_5$). **^{13}C NMR** ($CDCl_3$, 101 MHz, δ, ppm): -0.50 ($OSi\underline{C}H_3$), 11.63 ($Si(\underline{C}H(CH_3)_2)_3$), 18.84 ($Si(CH(\underline{C}H_3)_2)_3$), 104.03 ($\underline{C}{\equiv}CSi(i\text{-}Pr)_3$), 106.92 ($C{\equiv}\underline{C}Si(i\text{-}Pr)_3$), 121.46, 128.14, 128.77, 129.29 ($\underline{C}_6H_5$), 137.44 ($=\underline{C}C{\equiv}CSi(i\text{-}Pr)_3$), 146.03 ($=\underline{C}(H)C_6H_5$). **^{29}Si NMR** ($CDCl_3$, 79 MHz, δ, ppm): -108.84 (SiO_4, Q^4), -2.78 ($Si(i\text{-}Pr)_3$), 1.26 ($OSi(CH_3)_2$, Q^1). **FT IR (cm^{-1}):** 2942, 2891, 2864, 2105, 1560, 1494, 1462, 1382, 1252, 1078, 995, 882, 844, 788, 751, 734, 674, 656, 623, 601, 552, 509, 484. **MALDI TOF MS** - (m/z) ([M+Na], (%)): 3300.36. Elemental Anal. for $C_{168}H_{264}O_{20}Si_{24}$ (%): calcd.: C, 61.56; H, 8.12; found: C, 61.62; H, 8.15. Isolated yield = 89%. Colorless oil.

Compound 3g

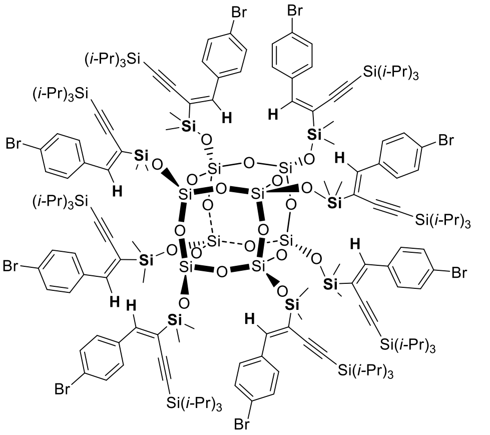

Chemical Formula: $C_{168}H_{256}Br_8O_{20}Si_{24}$
Molecular Weight: 3909,15

^{1}H NMR ($CDCl_3$, 300 MHz, δ, ppm): 0.33 (s, 48H, Si(C$\underline{H}_3$)$_2$), 1.09 (s, 168H, C$\underline{H}$(CH$_3$)$_2$, CH(C$\underline{H}_3$)$_2$), 6.88 (s, 8H, =C$\underline{H}$), 7.26, 7.29 (d, 16H, $J_{(H,H)}$= 8.99 Hz, C$_6\underline{H}_4$Br), 7.79, 7.82 (d, 16H, $J_{(H,H)}$= 8.58 Hz, C$_6\underline{H}_4$Br). **^{13}C NMR** ($CDCl_3$, 101 MHz, δ, ppm): -0.53 (OSi$\underline{C}$H$_3$), 11.56 (Si($\underline{C}$H(CH$_3$)$_2$)$_3$), 18.83 (Si(CH($\underline{C}$H$_3$)$_2$)$_3$), 105.82 ($\underline{C}$≡CSi(*i*-Pr)$_3$), 106.22 (C≡$\underline{C}$Si(*i*-Pr)$_3$), 122.44 ($\underline{C}_6$H$_4$Br), 122.87, 130.55, 131.36 ($\underline{C}_6$H$_4$Br), 136.36 (=$\underline{C}$C≡CSi(*i*-Pr)$_3$), 144.28 (=$\underline{C}$(H)C$_6$H$_4$Br). **^{29}Si NMR** ($CDCl_3$, 79 MHz, δ, ppm): -108.92 (SiO_4, Q^4), -2.41 (Si(*i*-Pr)$_3$), 1.37 (OSi(CH$_3$)$_2$, Q^1). **FT IR (cm^{-1}):** 2942, 2890, 2864, 2106, 1585, 1486, 1462, 1402, 1253, 1182, 1070, 1010, 882, 843, 813, 788, 659, 633, 598, 553, 511, 491. **MALDI TOF MS** - (m/z) ([M+Na], (%)): 3931.66. Elemental Anal. for $C_{168}H_{256}O_{20}Si_{24}$ (%): calcd.: C, 51.62; H, 6.60; found: C, 51.72; H, 6.65. Isolated yield = 91%. White solid.

Compound 3h

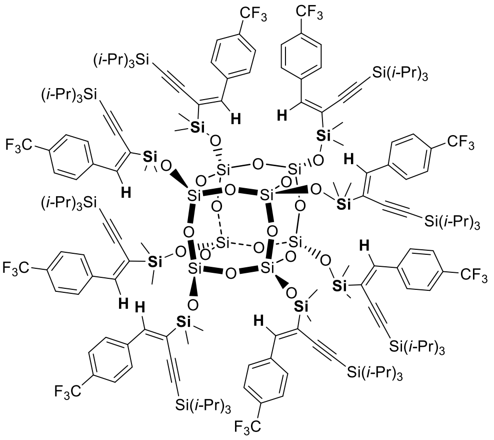

Chemical Formula: $C_{176}H_{256}F_{24}O_{20}Si_{24}$
Molecular Weight: 3821,97

^{1}H NMR ($CDCl_3$, 300 MHz, δ, ppm): 0.36 (s, 48H, Si(C$\underline{H}_3$)$_2$), 1.07 (s, 168H, C$\underline{H}$(CH$_3$)$_2$, CH(C$\underline{H}_3$)$_2$), 6.98 (s, 8H, =C$\underline{H}$), 7.34, 7.37 (d, 16H, $J_{(H,H)}$= 8.25 Hz, C$_6\underline{H}_4$CF$_3$), 7.97, 8.00 (d, 16H, $J_{(H,H)}$= 8.10 Hz, C$_6\underline{H}_4$CF$_3$). **^{13}C NMR** ($CDCl_3$, 101 MHz, δ, ppm): -0.58 (OSi$\underline{C}$H$_3$), 11.49 (Si($\underline{C}$H(CH$_3$)$_2$)$_3$), 18.72 (Si(CH($\underline{C}$H$_3$)$_2$)$_3$), 105.76 ($\underline{C}$≡CSi(*i*-Pr)$_3$), 106.82 (C≡$\underline{C}$Si(*i*-Pr)$_3$), 125.08, 129.05 ($\underline{C}_6$H$_4$CF$_3$, C$_6$H$_4\underline{C}$F$_3$), 140.14 (=$\underline{C}$C≡CSi(*i*-Pr)$_3$), 143.74 (=$\underline{C}$(H)C$_6$H$_4$CF$_3$). **^{29}Si NMR** ($CDCl_3$, 79 MHz, δ, ppm): -108.87 (SiO_4, Q^4), -2.16 (Si(*i*-Pr)$_3$), 1.43 (OSi(CH$_3$)$_2$, Q^1). **FT IR (cm^{-1}):** 2947, 2867, 2323, 2162, 1615, 1463, 1415, 1322, 1254, 1166, 1066, 883, 827, 790, 658, 608, 553. **MALDI TOF MS** - (m/z) ([M+Na], (%)): 3844.9. Elemental Anal. for $C_{176}H_{256}O_{20}Si_{24}$ (%): calcd.: C, 55.31; H, 6.75; found: C, 55.23; H, 6.69. Isolated yield = 95%. White solid.

Compound 3i

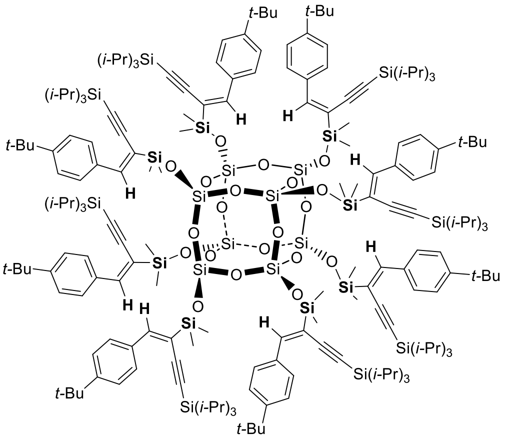

Chemical Formula: $C_{200}H_{328}O_{20}Si_{24}$
Molecular Weight: 3726,84

^{1}H NMR ($CDCl_3$, 400 MHz, δ, ppm): 0.40 (s, 48H, Si(C$\underline{H}_3$)$_2$), 1.17 (s, 168H, C$\underline{H}$(CH$_3$)$_2$, CH(C$\underline{H}_3$)$_2$), 1.25 (s, 72H, C(C$\underline{H}_3$)$_3$), 7.15 (s, 8H, =C$\underline{H}$), 6.88-7.23 (m, 32H, C$_6\underline{H}_4$). **^{13}C NMR** ($CDCl_3$, 101 MHz, δ, ppm): -0.43 (OSi$\underline{C}$H$_3$), 11.69 (Si($\underline{C}$H(CH$_3$)$_2$)$_3$), 18.89 (Si(CH($\underline{C}$H$_3$)$_2$)$_3$), 31.30 (C($\underline{C}$H$_3$)$_3$), 34.72 ($\underline{C}$(CH$_3$)$_3$), 103.43 ($\underline{C}$≡CSi(*i*-Pr)$_3$), 107.33 (C≡$\underline{C}$Si(*i*-Pr)$_3$), 120.21, 125.07, 129.22, 134.94 ($\underline{C}_6$H$_4$), 146.00 (=$\underline{C}$C≡CSi(*i*-Pr)$_3$), 151.90 (=$\underline{C}$(H)C$_6$H$_4$(*t*-Bu)). **^{29}Si NMR** ($CDCl_3$, 79 MHz, δ, ppm): -108.71 (SiO_4, Q^4), -2.87 (Si(*i*-Pr)$_3$), 1.38 (OSi(CH$_3$)$_2$, Q^1). **FT IR (cm^{-1}):** 2958, 2864, 1512, 1463, 1252, 1070, 882, 841, 825, 788, 659, 603, 557. **MALDI TOF MS** - (m/z) ([M+Na], (%)): 3748.5. Elemental Anal. for $C_{200}H_{328}O_{20}Si_{24}$ (%): calcd.: C, 64.46; H, 8.87; found: C, 64.35; H, 8.79. Isolated yield = 87%. Pale yellow solid.

Compound 3j

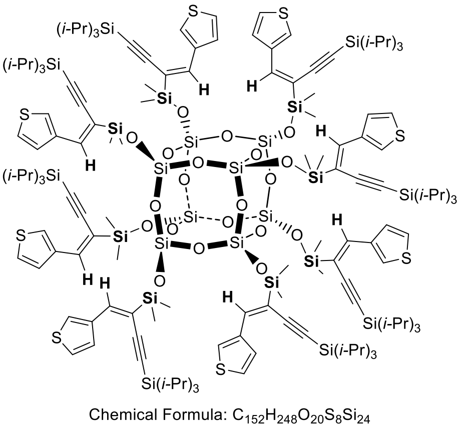

Chemical Formula: $C_{152}H_{248}O_{20}S_8Si_{24}$
Molecular Weight: 3326,16

^{1}H NMR ($CDCl_3$, 600 MHz, δ, ppm): 0.33 (s, 48H, Si(C$\underline{H}_3$)$_2$), 1.10 (s, 168H, C$\underline{H}$(CH$_3$)$_2$, CH(C$\underline{H}_3$)$_2$), 7.04 – 7.87 (m, 32H, =C$\underline{H}$, C$_4\underline{H}_3$S). **^{13}C NMR** ($CDCl_3$, 151 MHz, δ, ppm): -0.39 (OSi$\underline{C}$H$_3$), 11.64 (Si($\underline{C}$H(CH$_3$)$_2$)$_3$), 18.86 (Si(CH($\underline{C}$H$_3$)$_2$)$_3$), 103.46 ($\underline{C}$≡CSi(*i*-Pr)$_3$), 107.46 (C≡$\underline{C}$Si(*i*-Pr)$_3$), 119.61, 124.73, 126.72, 128.39 (C$_4$H$_3$S), 139.61 (=$\underline{C}$C≡CSi(*i*-Pr)$_3$), 140.18 (=$\underline{C}$(H)C$_4$H$_3$S). **^{29}Si NMR** ($CDCl_3$, 119 MHz, δ, ppm): -108.88 (SiO_4, Q^4), -2.78 (Si(*i*-Pr)$_3$), 1.53 (OSi(CH$_3$)$_2$, Q^1). **FT IR (cm^{-1}):** 2942, 2891, 2864, 1574, 1462, 1382, 1252, 1160, 1069, 995, 881, 840, 789, 734, 658, 640, 553, 507. **MALDI TOF MS** - (m/z) ([M+Na], (%)): 3348.1. Elemental Anal. for $C_{152}H_{248}O_{20}Si_{24}$ (%): calcd.: C, 54.89; H, 7.52; found: C, 54.76; H, 7.47. Isolated yield = 82%. Colorless oil.

Compound 3k

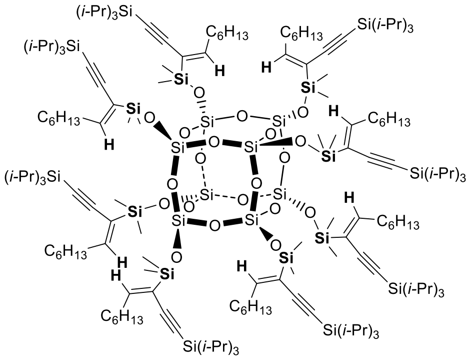

Chemical Formula: $C_{168}H_{328}O_{20}Si_{24}$
Molecular Weight: 3342,49

^{1}H NMR ($CDCl_3$, 600 MHz, δ, ppm): 0.28 (s, 48H, Si($C\underline{H}_3$)$_2$), 0.86-090 (m, 24H, (CH_2)$_5$C$\underline{H}_3$), 1.08-1.09 (m, 168H, C$\underline{H}$(CH_3)$_2$, CH(C$\underline{H}_3$)$_2$), 1.26-1.28 (m, 48H, CH_2CH_2(C$\underline{H}_2$)$_3CH_3$), 1.39-1.41 (m, 16H, CH_2C$\underline{H}_2$(CH_2)$_3CH_3$), 2.37-2.39 (m, 16H, C$\underline{H}_2$(CH_2)$_4CH_3$), 6.37 (t, 8H, =C$\underline{H}$). **^{13}C NMR** ($CDCl_3$, 101 MHz, δ, ppm): -0.38 (OSi$\underline{C}H_3$), 11.53 (Si($\underline{C}$H(CH_3)$_2$)$_3$), 14.19 ((CH_2)$_5\underline{C}H_3$), 18.83 (Si(CH($\underline{C}H_3$)$_2$)$_3$), 22.77 ((CH_2)$_4\underline{C}H_2CH_3$), 28.77, 29.32 ((CH_2)$_2$($\underline{C}H_2$)$_2CH_2CH_3$), 31.75, 32.85 (($\underline{C}H_2$)$_2$(CH_2)$_2CH_2CH_3$), 98.24 ($\underline{C}$≡CSi(*i*-Pr)$_3$), 105.73 (C≡$\underline{C}$Si(*i*-Pr)$_3$), 123.05 (=$\underline{C}$C≡CSi(*i*-Pr)$_3$), 153.83 (=$\underline{C}$(H)C_6H_{13}). **^{29}Si NMR** ($CDCl_3$, 79 MHz, δ, ppm): -109.12 (SiO_4, Q^4), -2.66 (Si(*i*-Pr)$_3$), -0.38 (OSi(CH_3)$_2$, Q^1). **FT IR (cm^{-1}):** 2957, 2926, 2864, 2124, 1463, 1253, 1081, 995, 882, 833, 789, 674, 557. **MALDI TOF MS** - (m/z) ([M+Na], (%)): 3365.43. Elemental Anal. for $C_{168}H_{328}O_{20}Si_{24}$ (%): calcd.: C, 60.37; H, 9.89; found: C, 60.28; H, 9.81. Isolated yield = 83%. Colorless oil.

Compound 3l

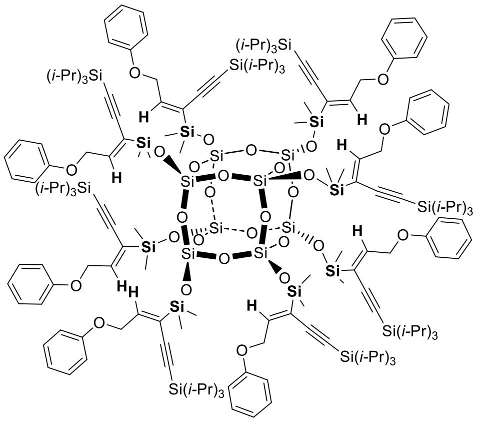

Chemical Formula: $C_{176}H_{280}O_{28}Si_{24}$
Molecular Weight: 3518,19

^{1}H NMR ($CDCl_3$, 600 MHz, δ, ppm): 0.32 (s, 48H, Si(C$\underline{H}_3$)$_2$), 1.08 (s, 168H, C$\underline{H}$(CH_3)$_2$, CH(C$\underline{H}_3$)$_2$), 4.82, 4.83 (d, 16H, $J_{(H,H)}$= 6.31 Hz, OC$\underline{H}_2$), 6.55 (t, 8H, =C$\underline{H}$), 6.54-7.23 (m, 40H, $C_6\underline{H}_5$). **^{13}C NMR** ($CDCl_3$, 101 MHz, δ, ppm): -0.82 (OSi$\underline{C}H_3$), 11.42 (Si($\underline{C}$H(CH_3)$_2$)$_3$), 18.82 (Si(CH($\underline{C}H_3$)$_2$)$_3$), 87.66 (O$\underline{C}H_2$), 102.12 ($\underline{C}$≡CSi(*i*-Pr)$_3$), 104.19 (C≡$\underline{C}$Si(*i*-Pr)$_3$), 114.64, 120.72, 126.15, 129.48 ($\underline{C}_6H_5$), 147.35 (=$\underline{C}$C≡CSi(*i*-Pr)$_3$), 158.72 (=$\underline{C}$(H)$CH_2OC_6H_5$). **^{29}Si NMR** ($CDCl_3$, 79 MHz, δ, ppm): -109.35 (SiO_4, Q^4), -2.01 (Si(*i*-Pr)$_3$), -0.10 (OSi(CH_3)$_2$, Q^1). **FT IR (cm^{-1}):** 2942, 2865, 1600, 1495, 1462, 1253, 1077, 881, 828, 791, 750, 675, 557, 507. Elemental Anal. for $C_{176}H_{280}O_{28}Si_{24}$ (%): calcd.: C, 60.09; H, 8.02; found: C, 59.92; H, 7.97. Isolated yield = 88%. Pale yellow oil.

Compound 3m

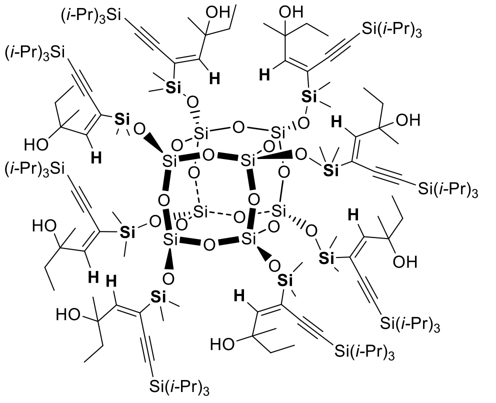

Chemical Formula: $C_{152}H_{296}O_{28}Si_{24}$
Molecular Weight: 3246,05

^{1}H NMR ($CDCl_3$, 300 MHz, δ, ppm): 0.31 (s, 48H, Si($C\underline{H}_3$)$_2$), 0.91 (t, 24H, $J_{(H,H)}$= 7.39 Hz, $CH_2C\underline{H}_3$), 1.07 (s, 168H, $C\underline{H}(CH_3)_2$, $CH(C\underline{H}_3)_2$), 1.34 (s, 24H, $C(C\underline{H}_3)(OH)CH_2CH_3$), 1.62-1.71 (m, 16H, $C\underline{H}_2CH_3$), 3.79 (s, 8H, $C(CH_3)(O\underline{H})CH_2CH_3$), 6.36 (s, 1H, =$C\underline{H}$). **^{13}C NMR** ($CDCl_3$, 151 MHz, δ, ppm): -0.48, -0.40 ($OSi\underline{C}H_3$), 8.40 ($C(CH_3)(OH)CH_2\underline{C}H_3$), 11.47 ($Si(\underline{C}H(CH_3)_2)_3$), 18.75 ($Si(CH(\underline{C}H_3)_2)_3$), 27.56 ($C(\underline{C}H_3)(OH)CH_2CH_3$), 35.11 ($C(CH_3)(OH)\underline{C}H_2CH_3$), 76.16 ($\underline{C}(CH_3)(OH)C_2H_5$), 104.23 ($\underline{C}$≡CSi(*i*-Pr)$_3$), 104.55 (C≡$\underline{C}$Si(*i*-Pr)$_3$), 120.27 61 (=$\underline{C}$C≡CSi(*i*-Pr)$_3$), 158.69 (=$\underline{C}(H)C(CH_3)(OH)C_2H_5$). **^{29}Si NMR** ($CDCl_3$, 119 MHz, δ, ppm): -108.95 (SiO_4, Q^4), -2.10 (Si(*i*-Pr)$_3$), 0.97 ($OSi(CH_3)_2$, Q^1). **FT IR (cm^{-1}):** 2960, 2962, 2865, 1462, 1366, 1253, 1158, 1081, 995, 918, 882, 840, 790, 674, 657, 612, 557. **MALDI TOF MS** - (m/z) ([M+Na], (%)): 3268.6. Elemental Anal. for $C_{152}H_{296}O_{28}Si_{24}$ (%): calcd.: C, 56.24; H, 9.19; found: C, 56.32; H, 9.25. Isolated yield = 78%. Colorless oil.

6. NMR spectra

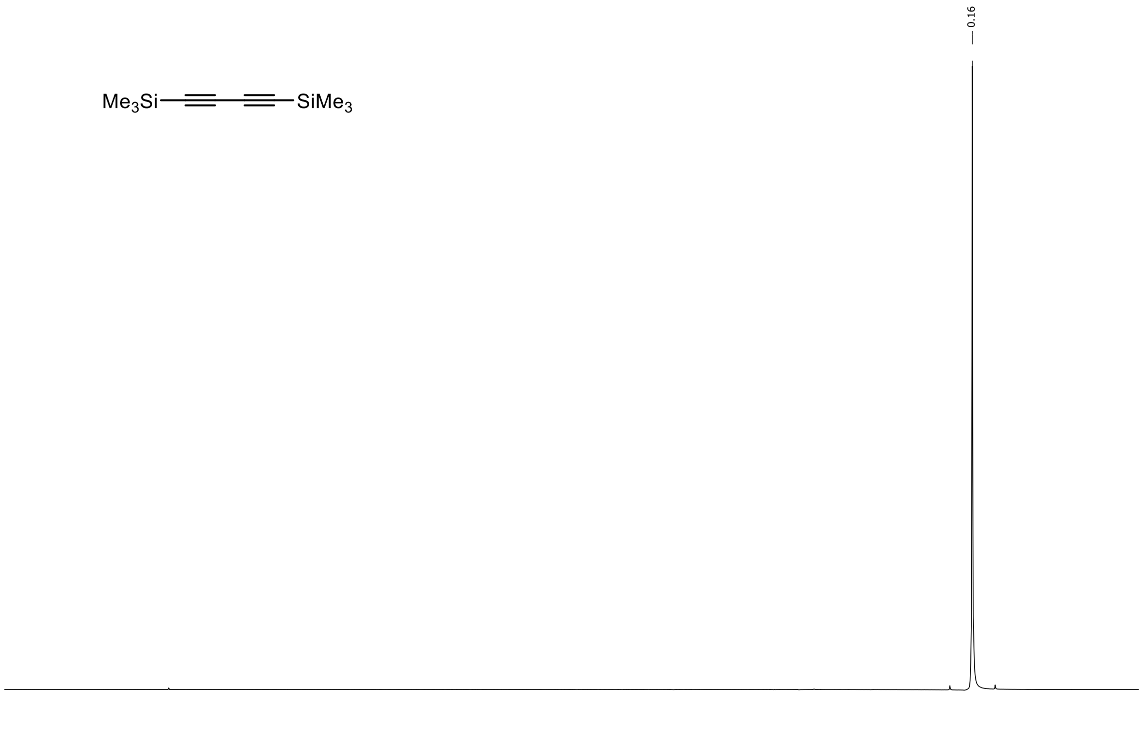

Figure S3. ^{1}H NMR spectrum of **2a**.

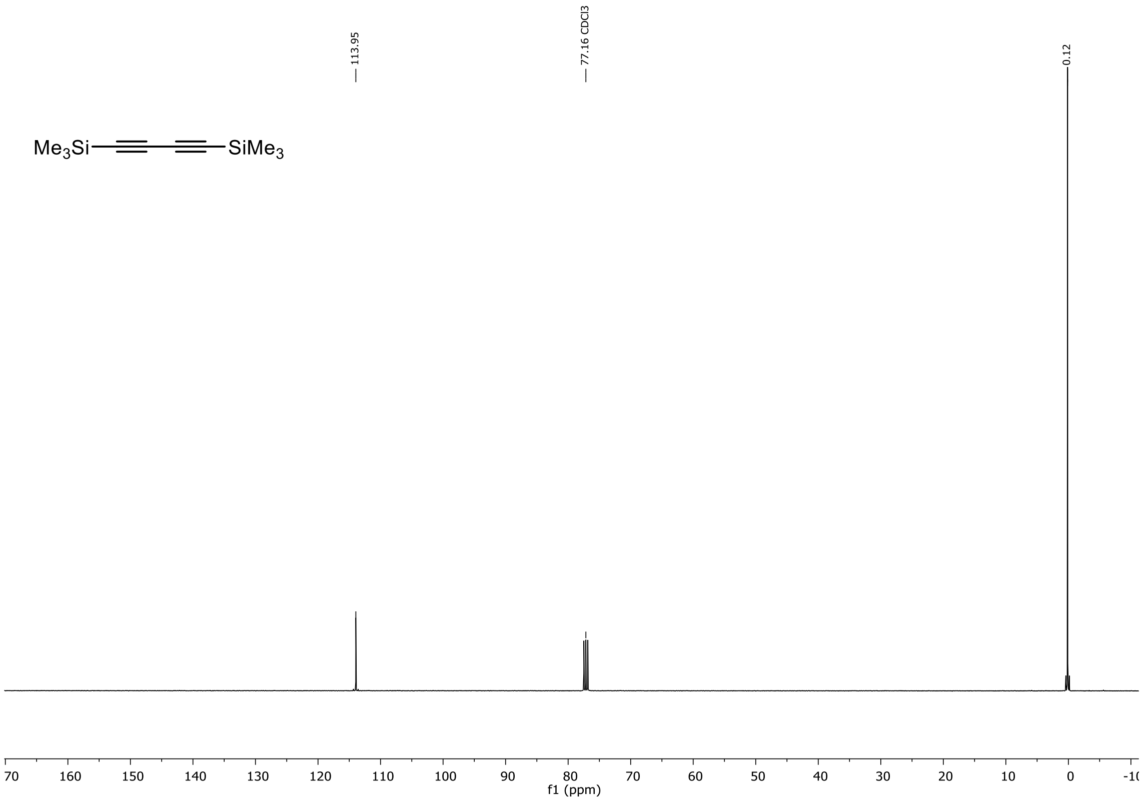

Figure S4. ^{13}C NMR spectrum of **2a**.

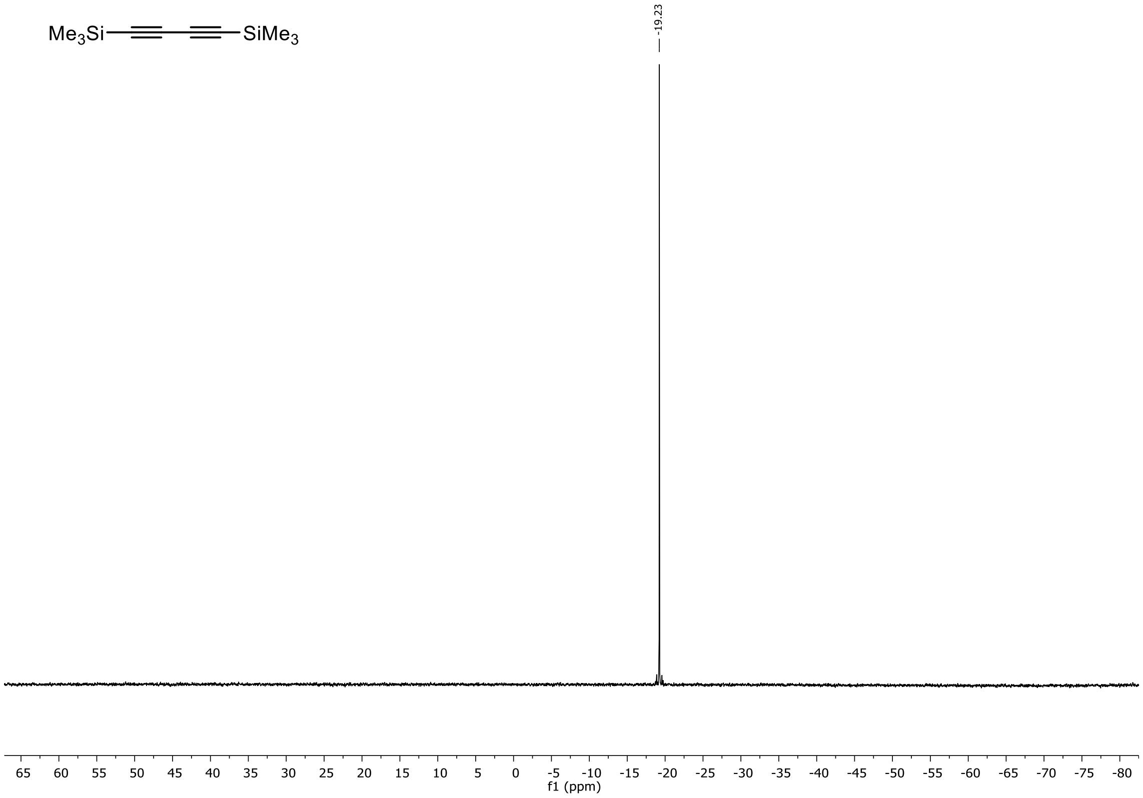

Figure S5. ^{29}Si NMR spectrum of **2a**.

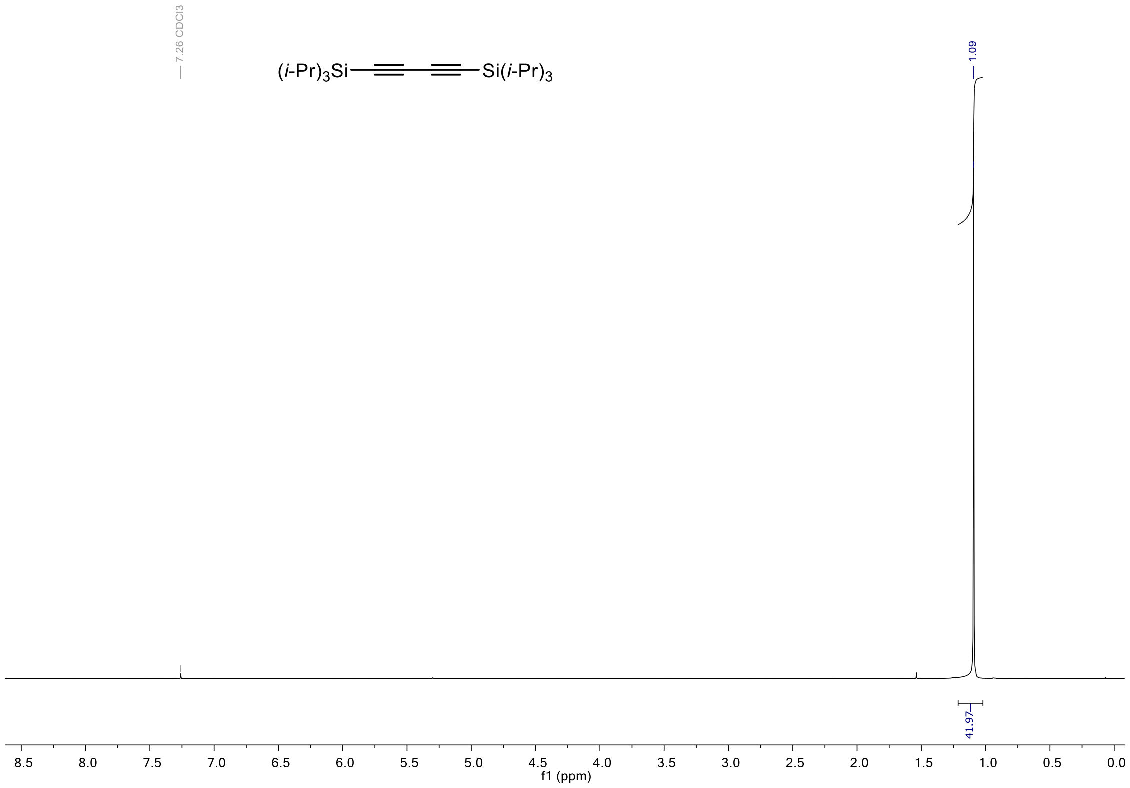

Figure S6. ^{1}H NMR spectrum of **2b**.

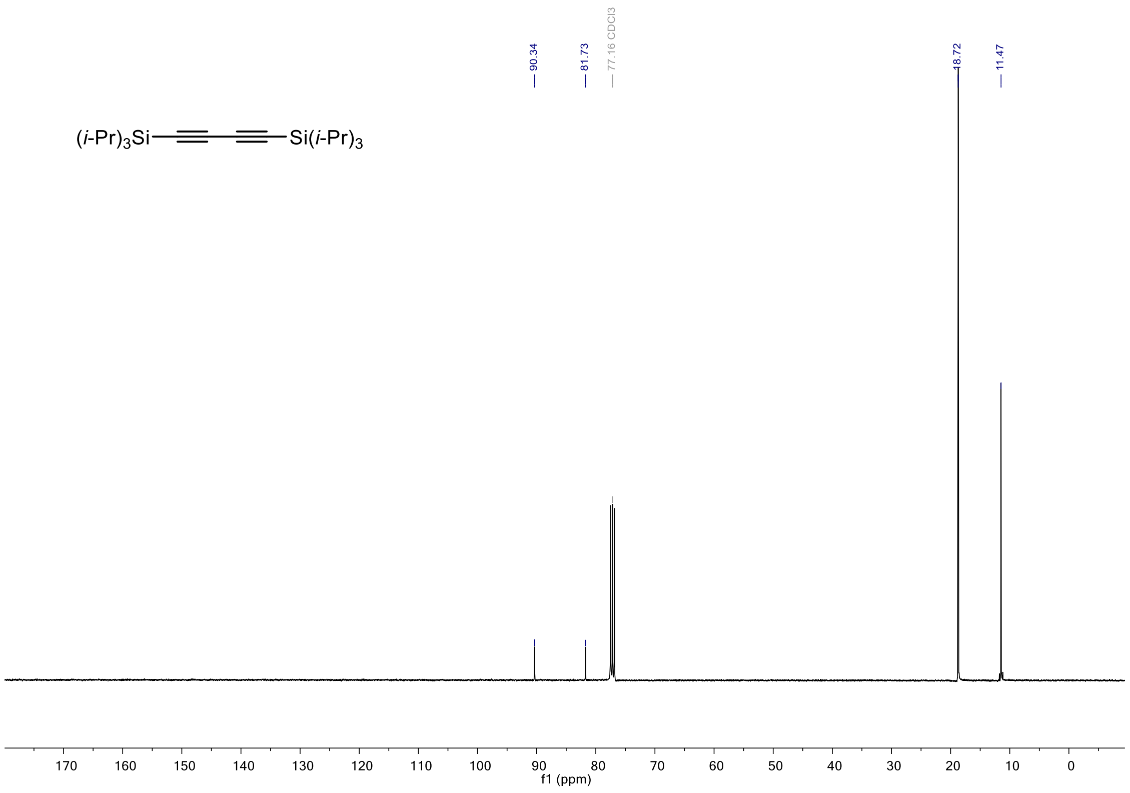

Figure S7. ^{13}C NMR spectrum of **2b**.

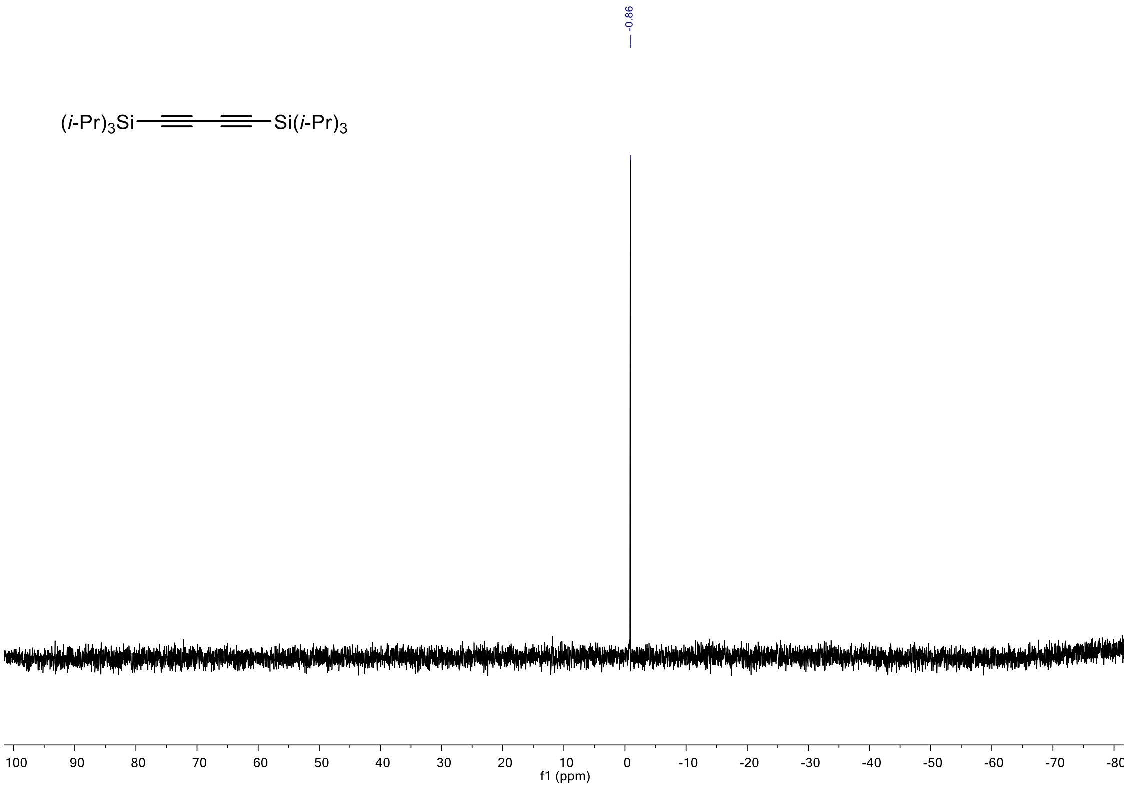

Figure S8. ^{29}Si NMR spectrum of **2b**.

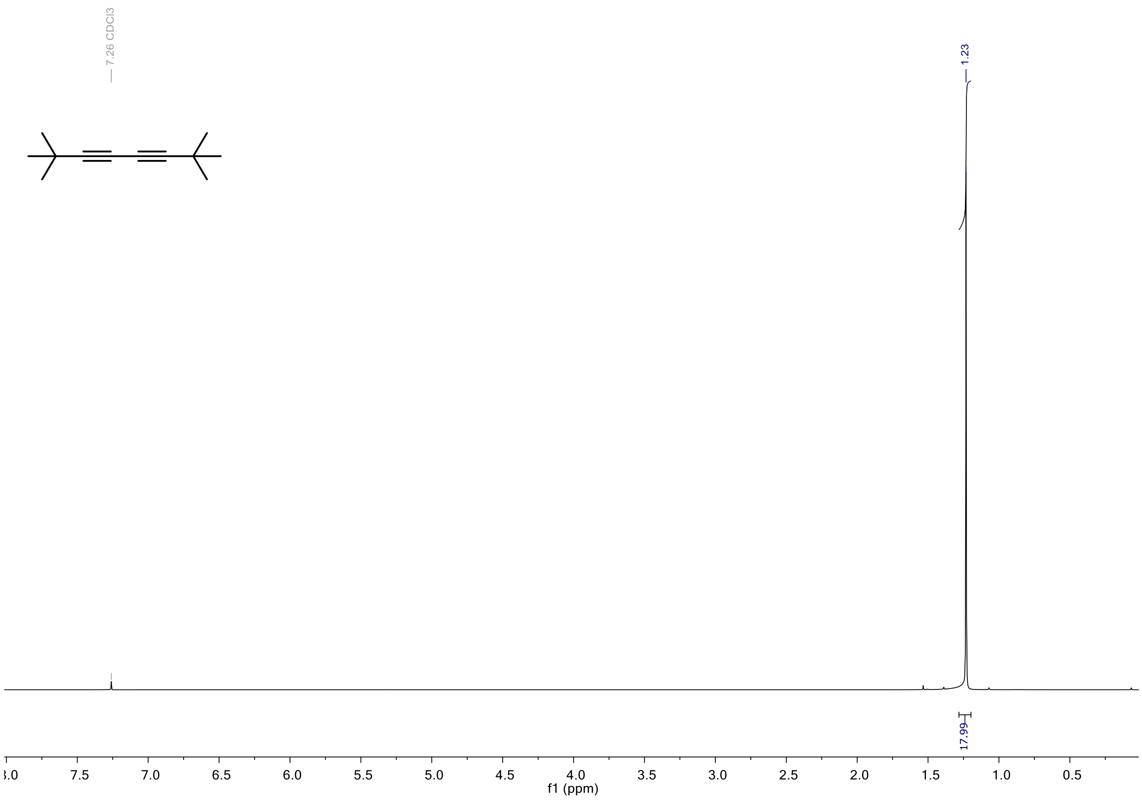

Figure S9. ^{1}H NMR spectrum of **2c**.

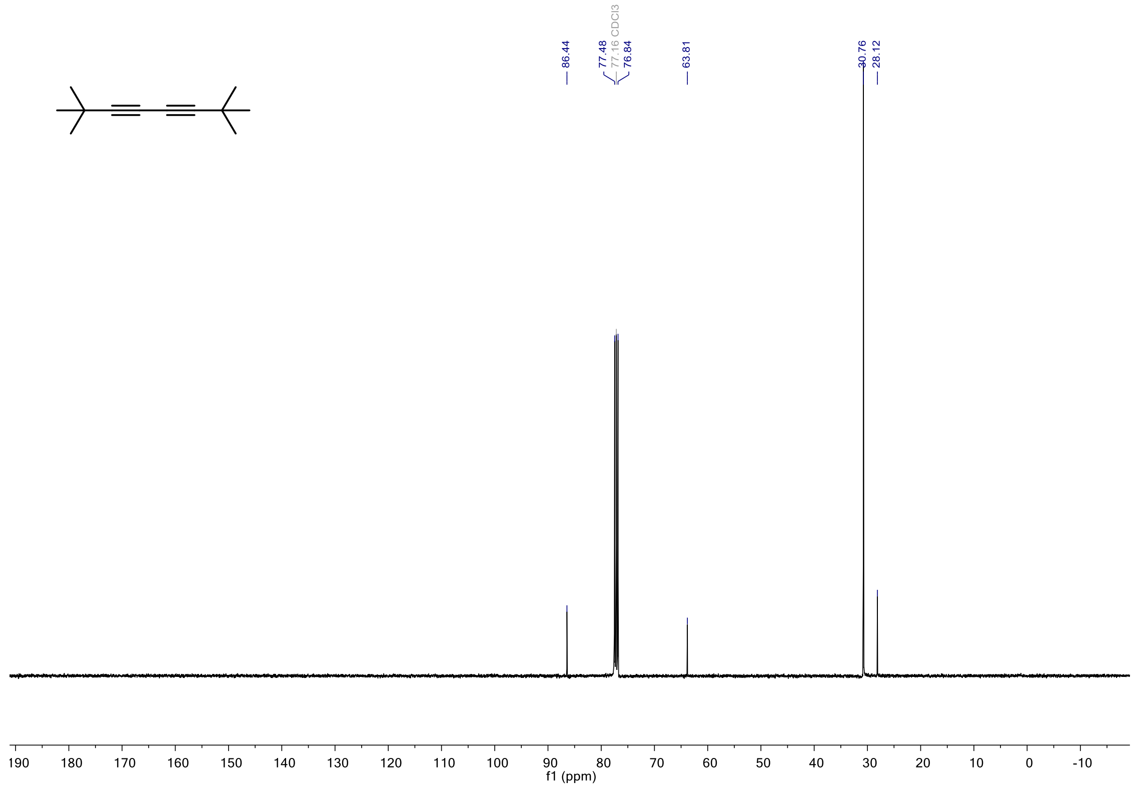

Figure S10. ^{13}C NMR spectrum of **2c**.

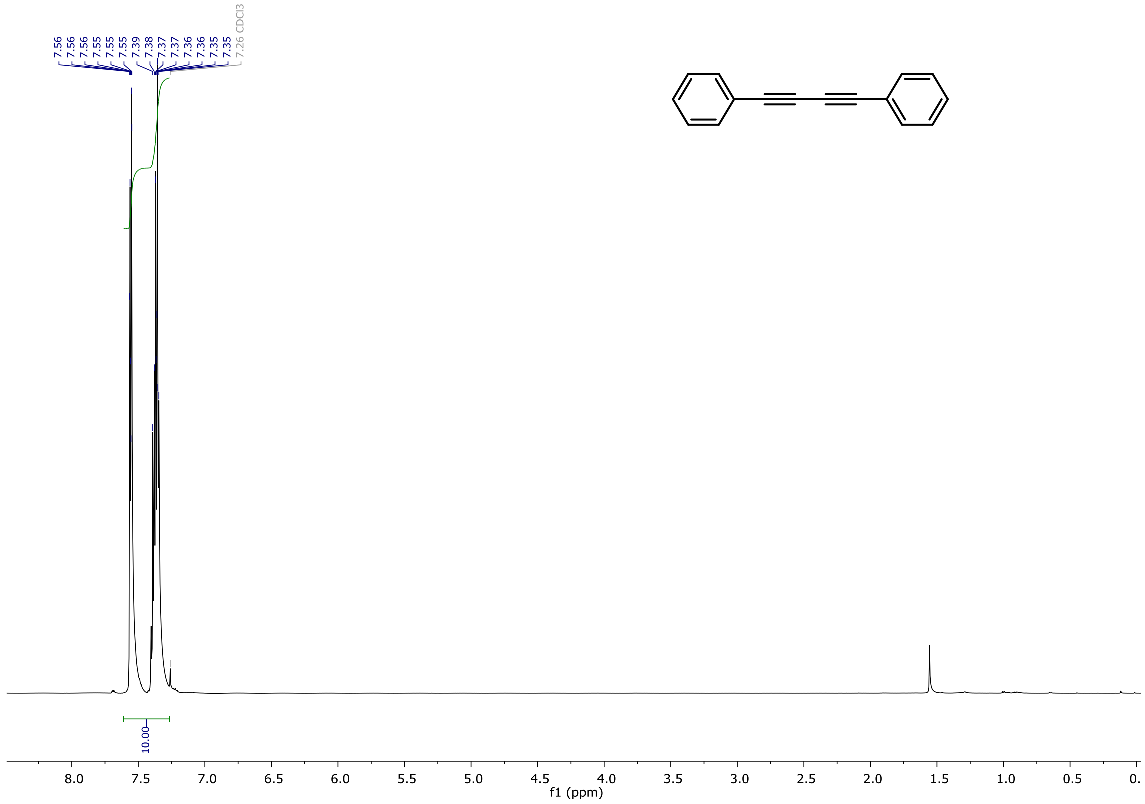

Figure S11. ^{1}H NMR spectrum of **2d**.

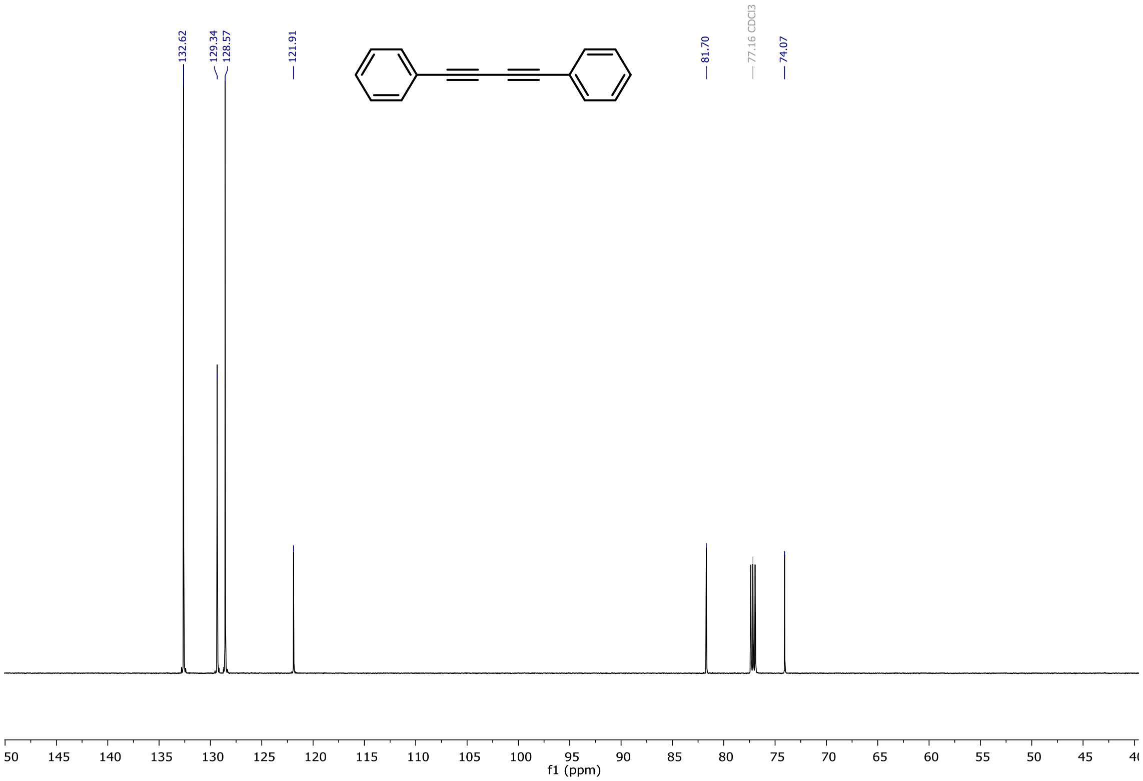

Figure S12. ^{13}C NMR spectrum of **2d**.

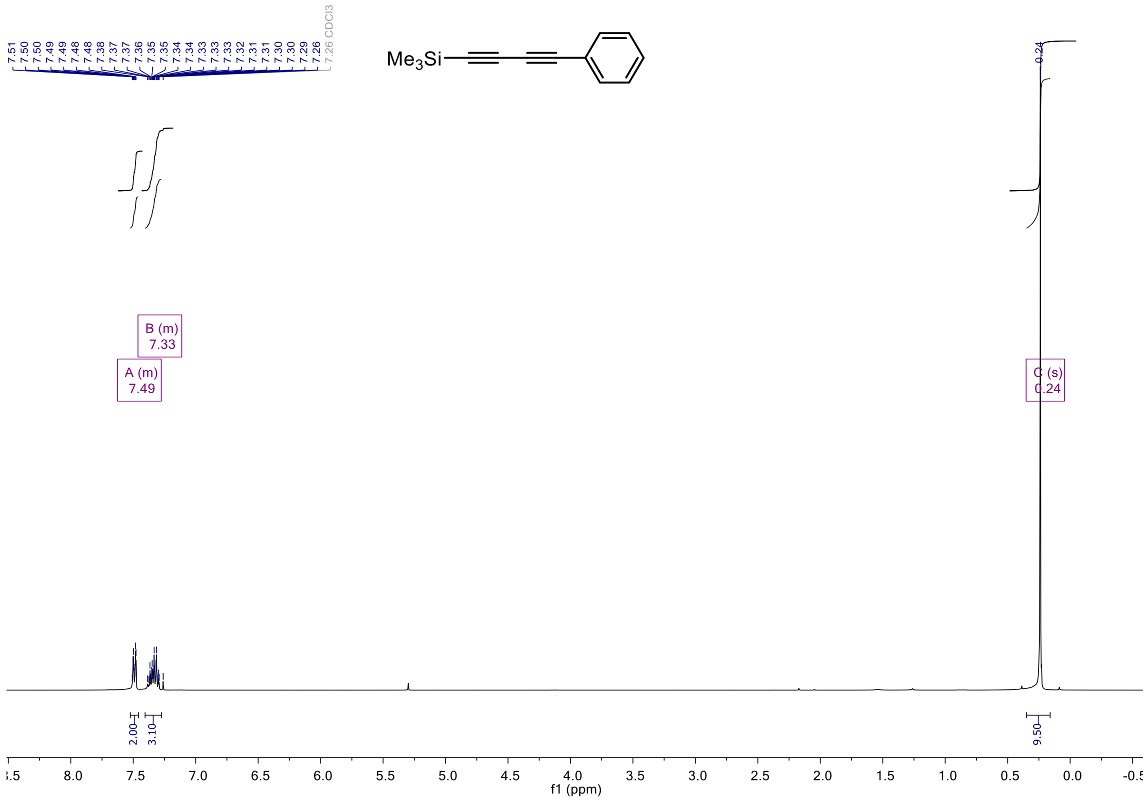

Figure S13. ^{1}H NMR spectrum of **2e**.

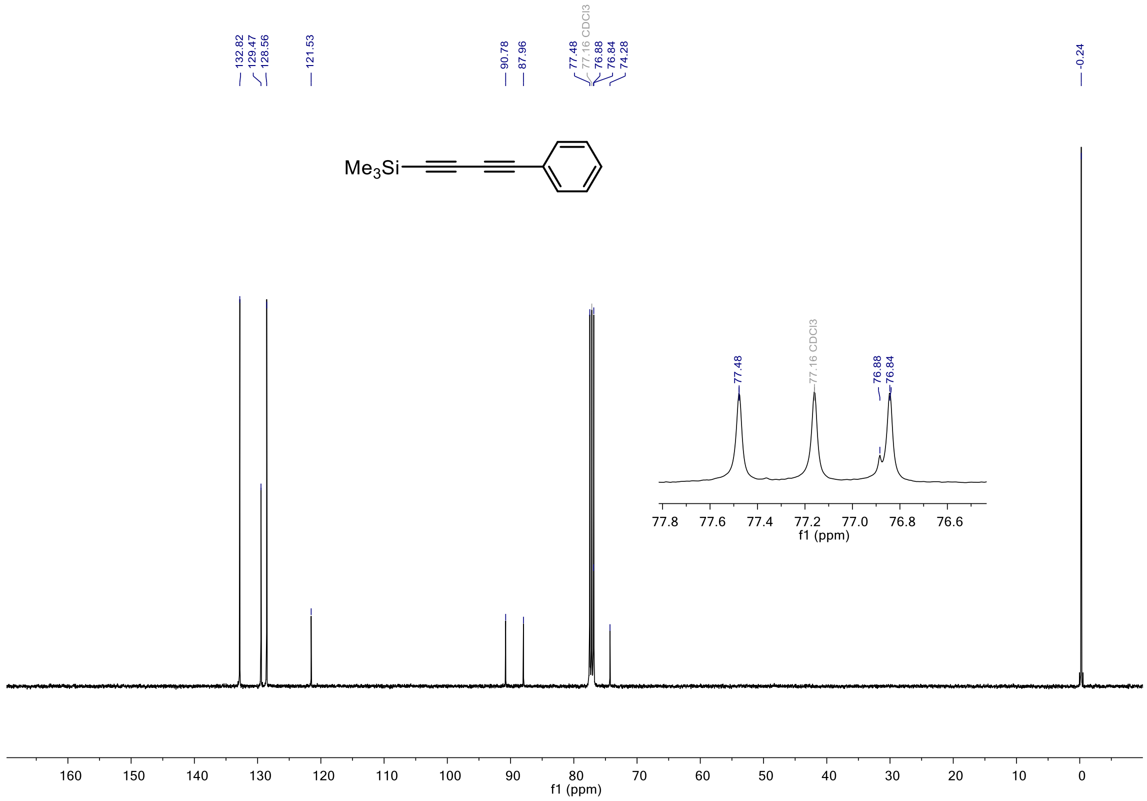

Figure S14. ^{13}C NMR spectrum of **2e**.

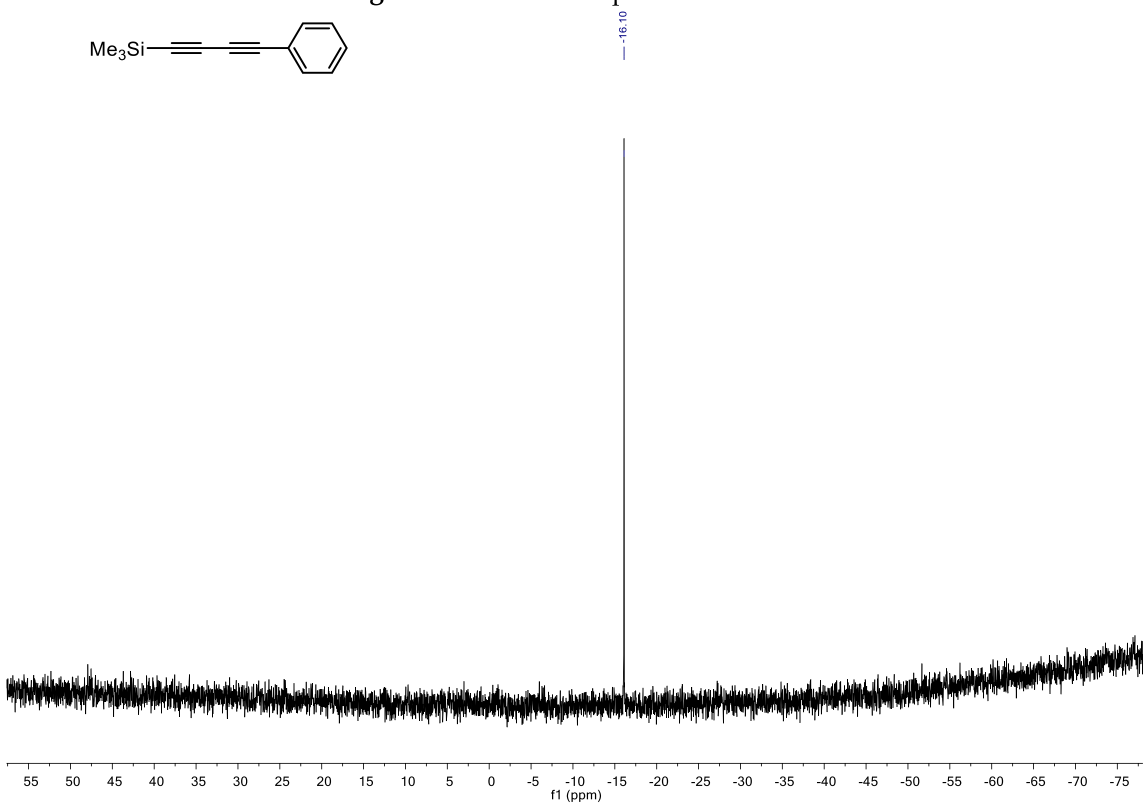

Figure S15. ^{29}Si NMR spectrum of **2e**.

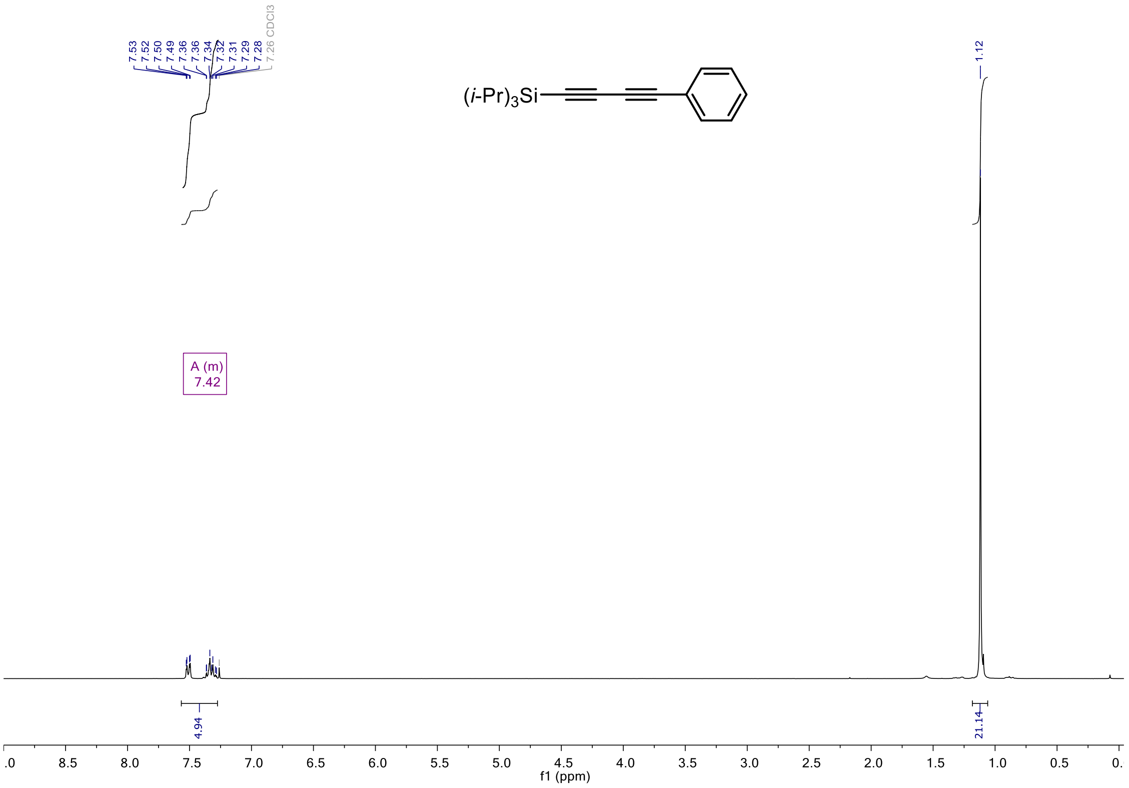

Figure S16. ^{1}H NMR spectrum of **2f**.

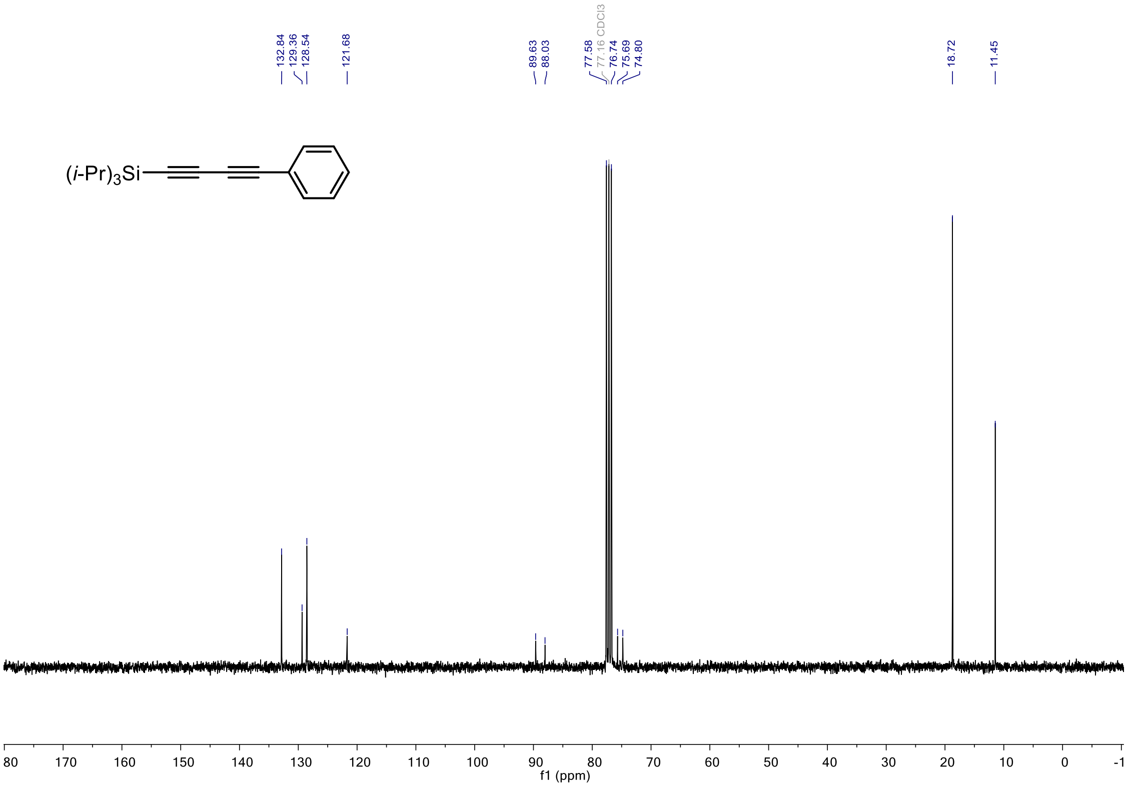

Figure S17. ^{13}C NMR spectrum of **2f**.

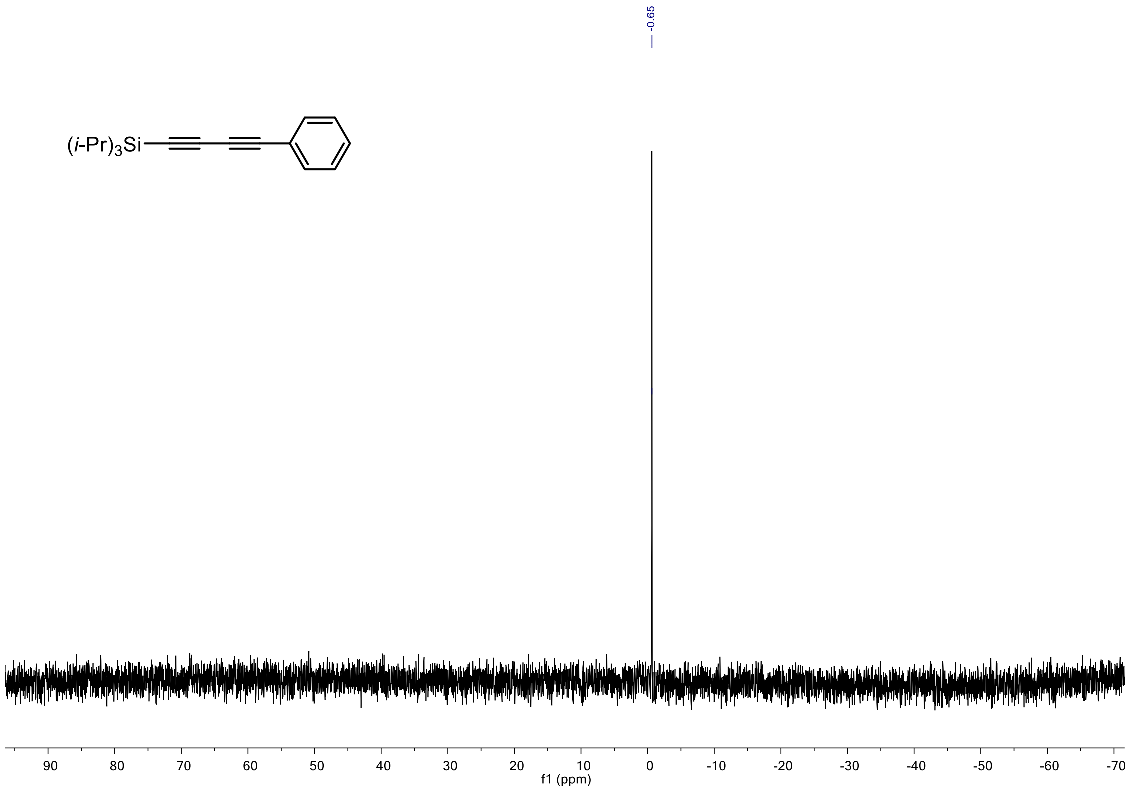

Figure S18. ^{29}Si NMR spectrum of **2f**.

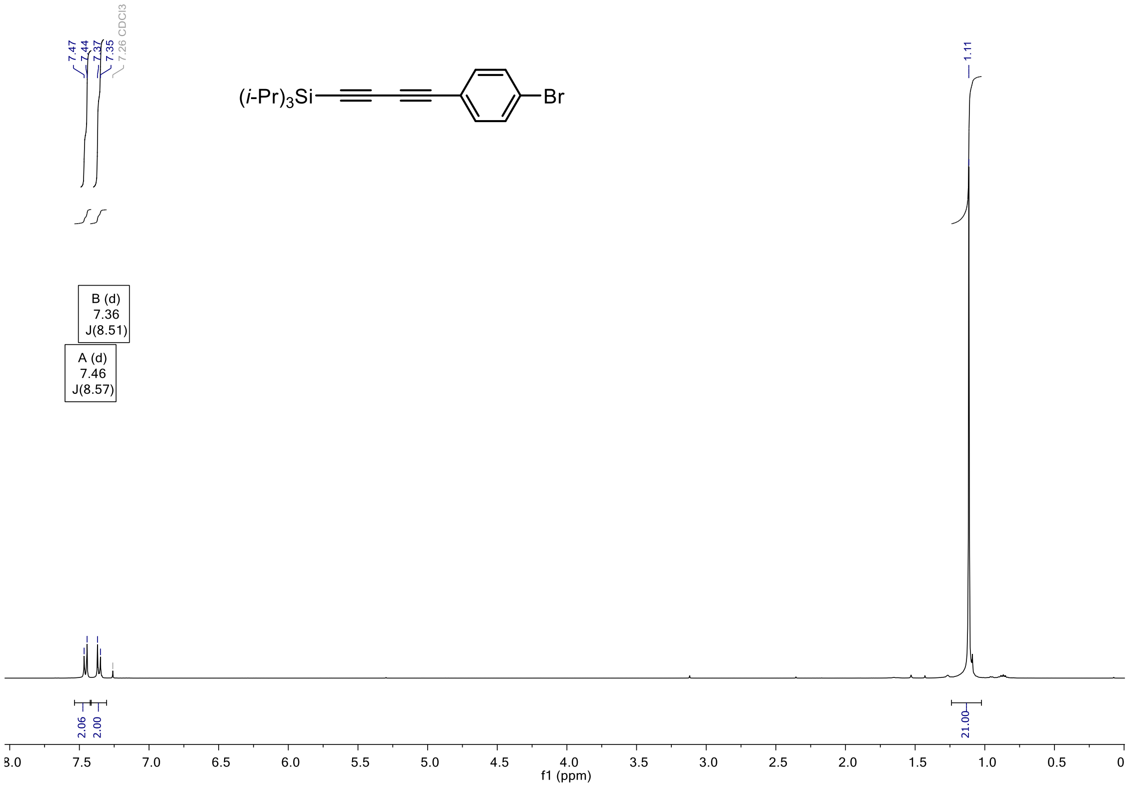

Figure S19. ^{1}H NMR spectrum of **2g**.

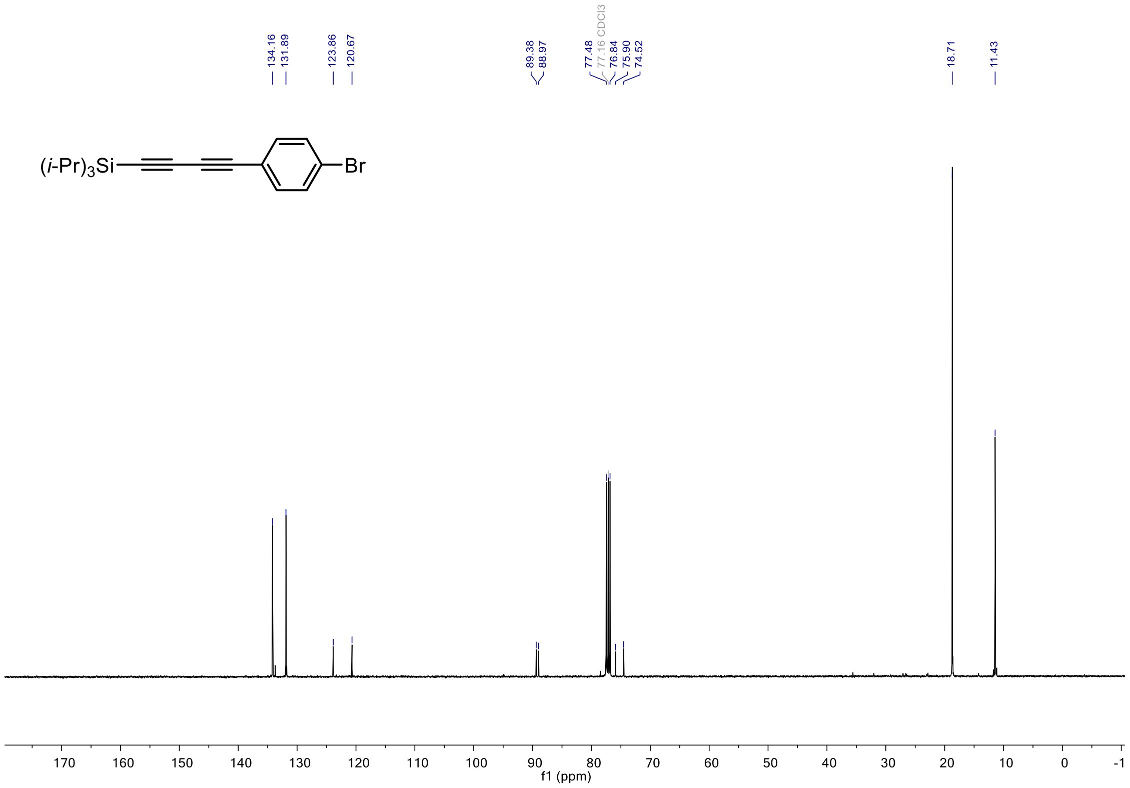

Figure S20. ^{13}C NMR spectrum of **2g**.

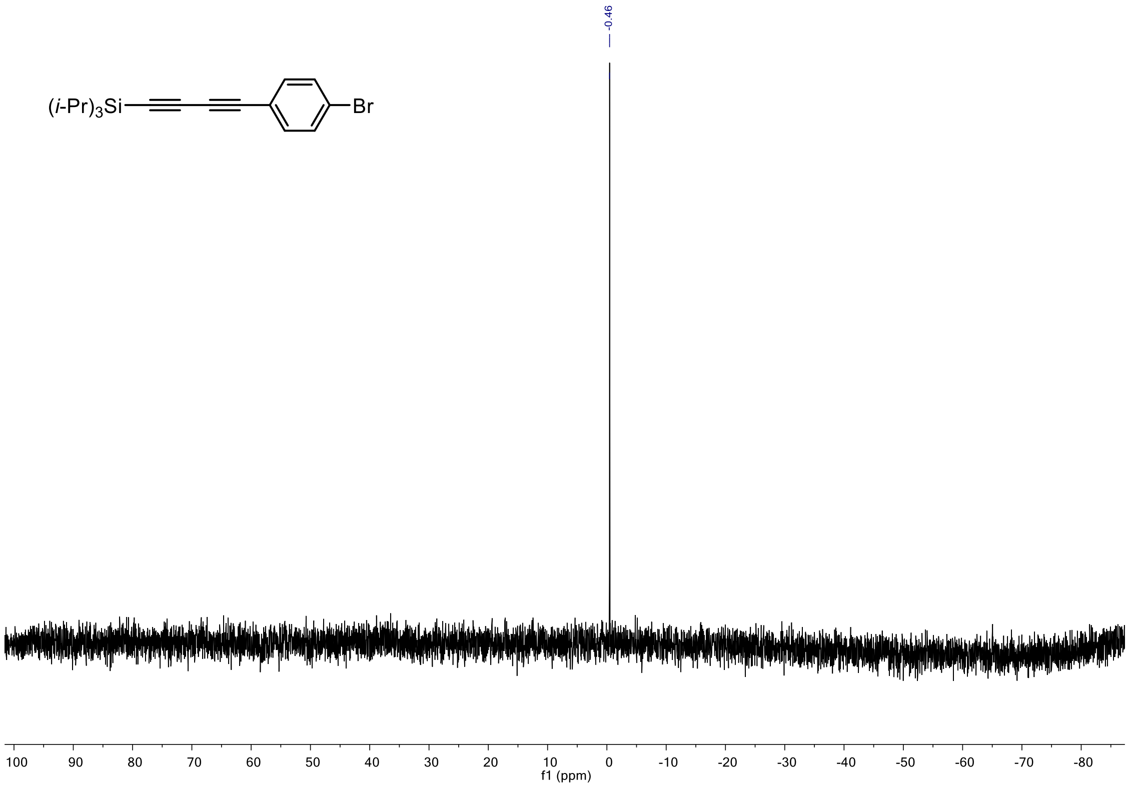

Figure S21. ^{29}Si NMR spectrum of **2g**.

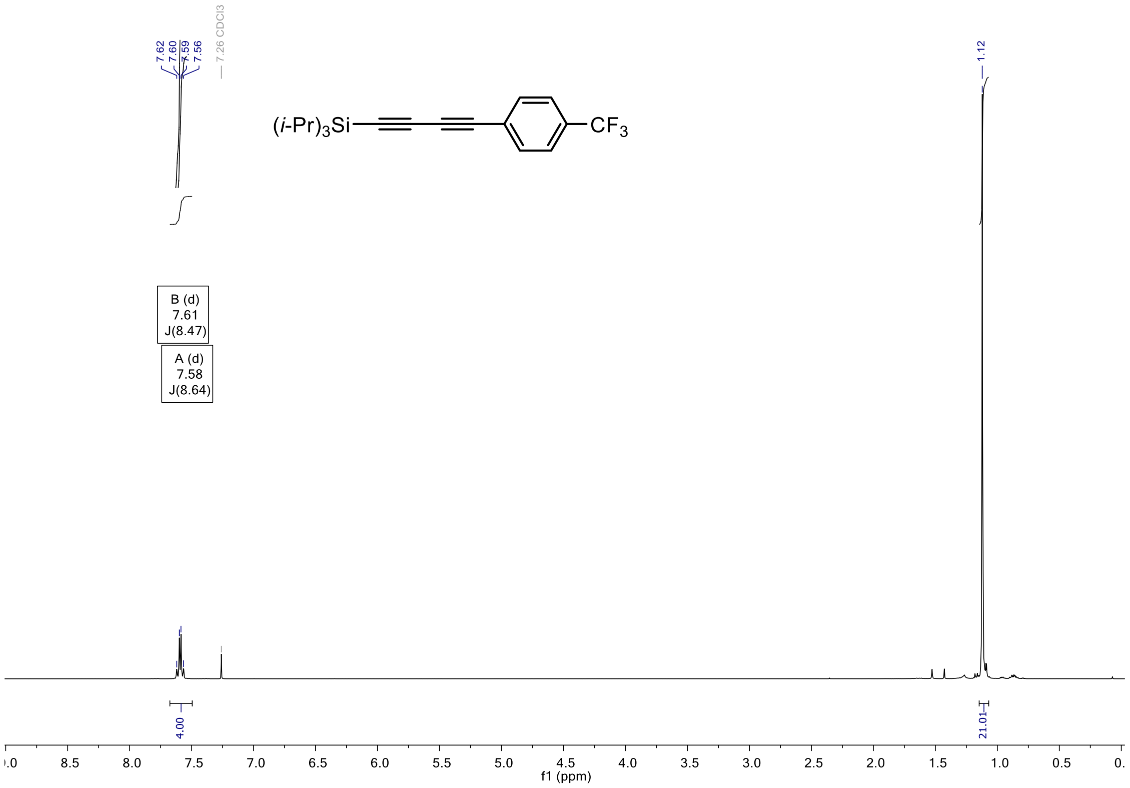

Figure S22. ^{1}H NMR spectrum of **2h**.

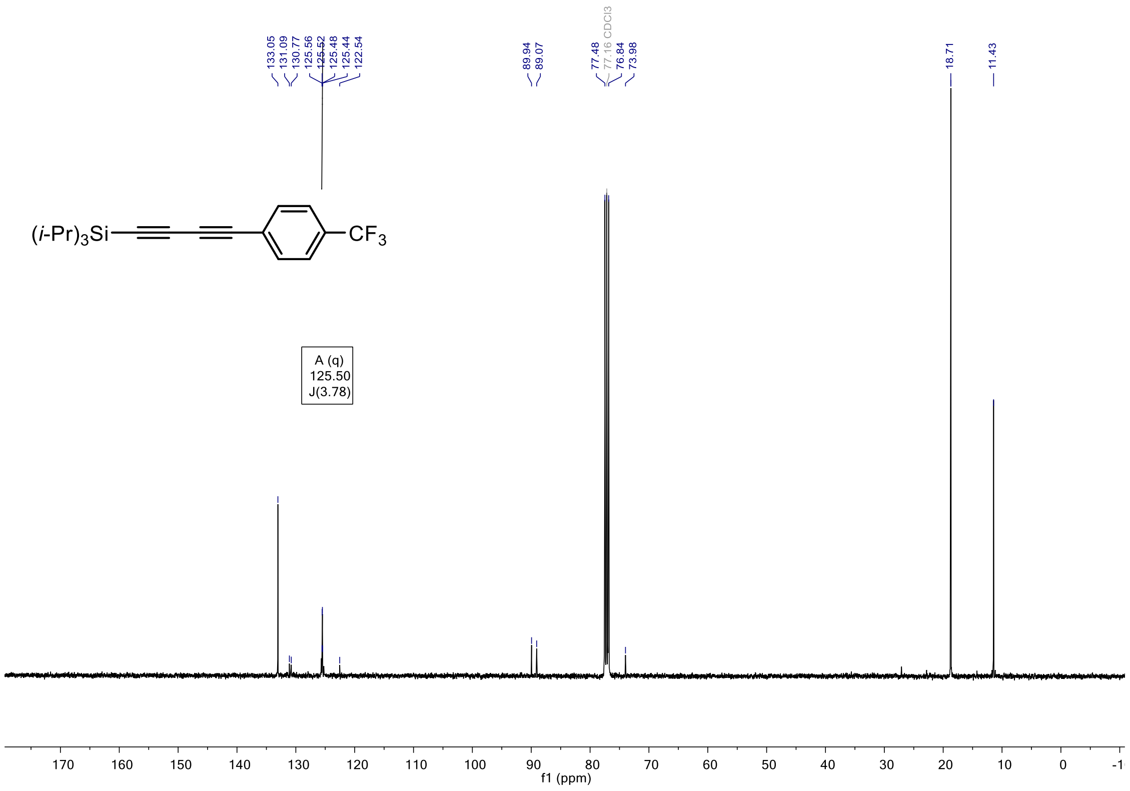

Figure S23. ^{13}C NMR spectrum of **2h**.

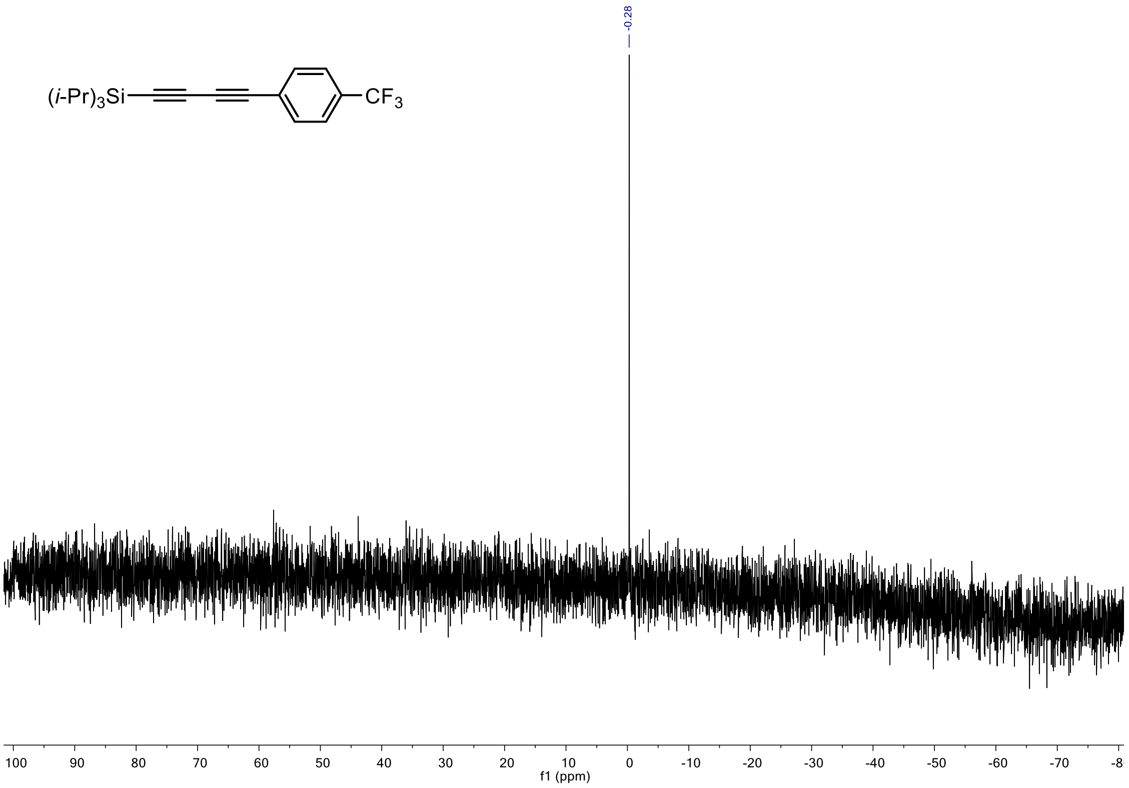

Figure S24. ^{29}Si NMR spectrum of **2h**.

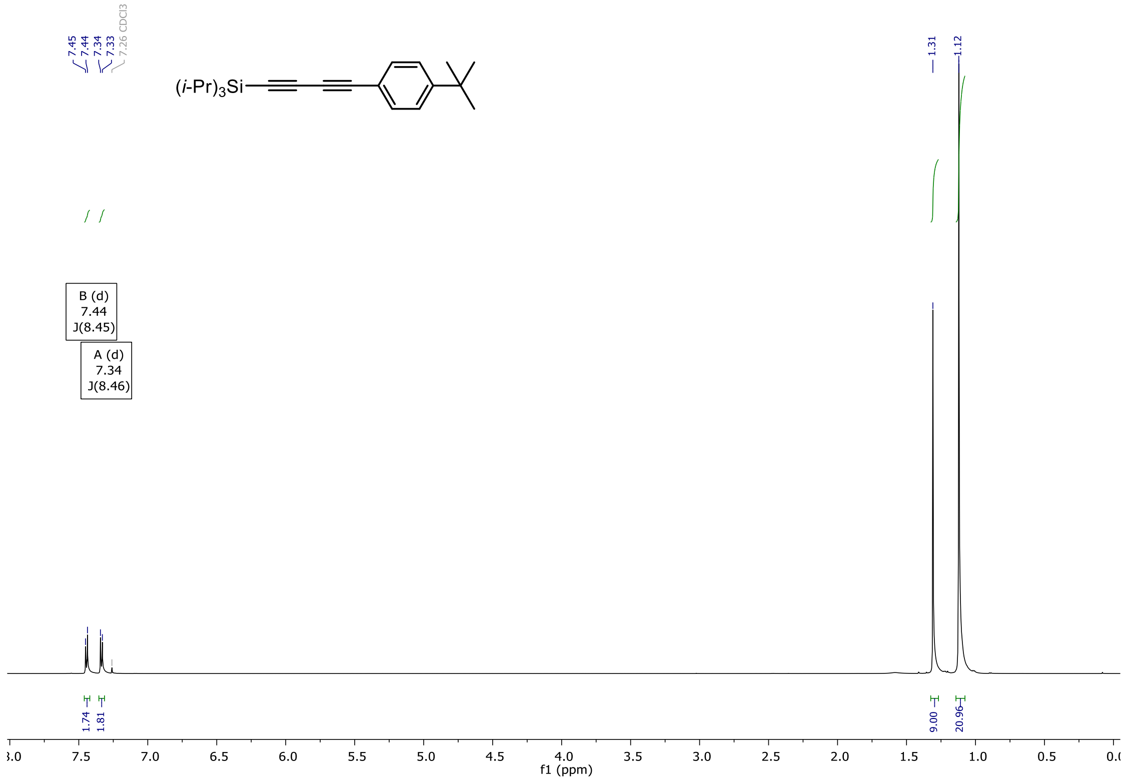

Figure S25. ^{1}H NMR spectrum of **2i**.

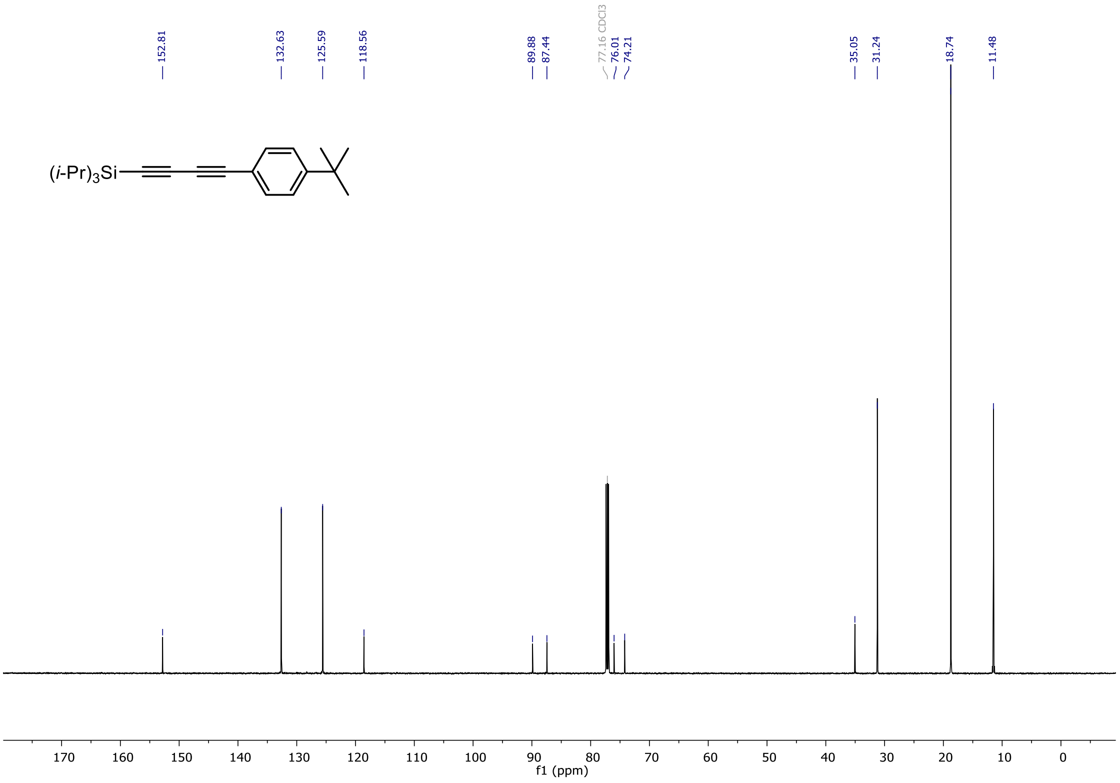

Figure S26. ^{13}C NMR spectrum of **2i**.

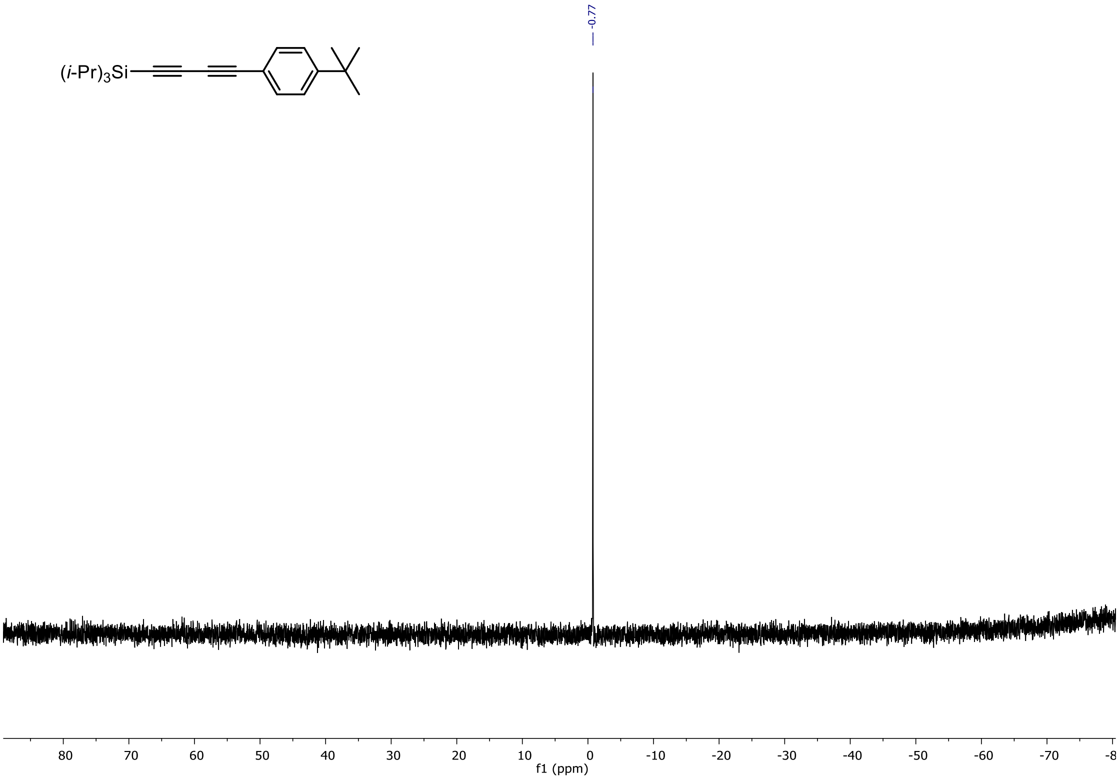

Figure S27. ^{29}Si NMR spectrum of **2i**.

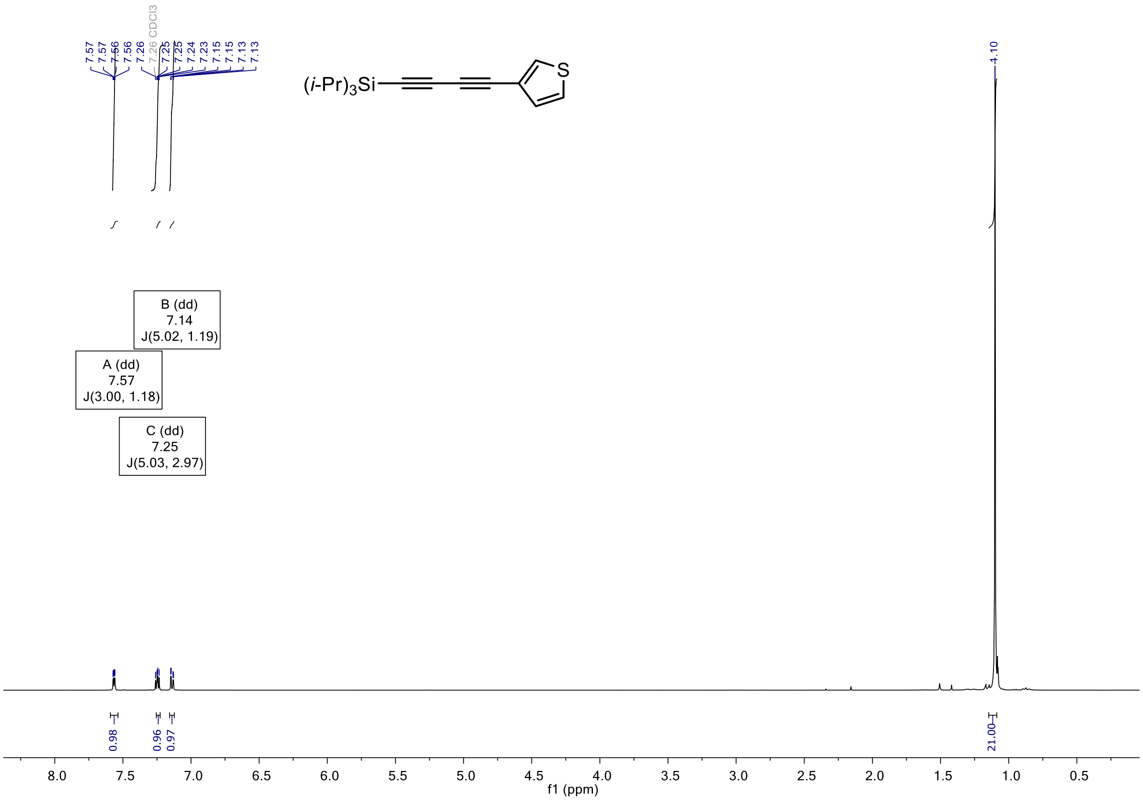

Figure S28. ^{1}H NMR spectrum of **2j**.

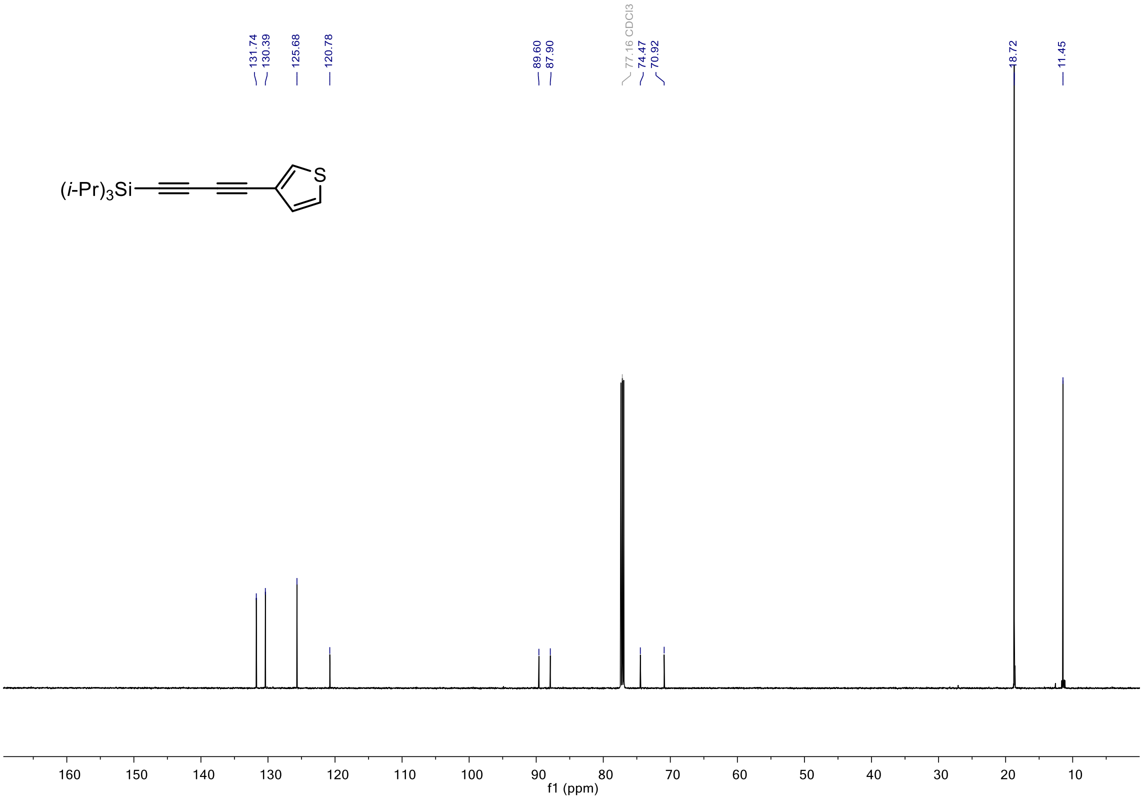

Figure S29. ^{13}C NMR spectrum of **2j**.

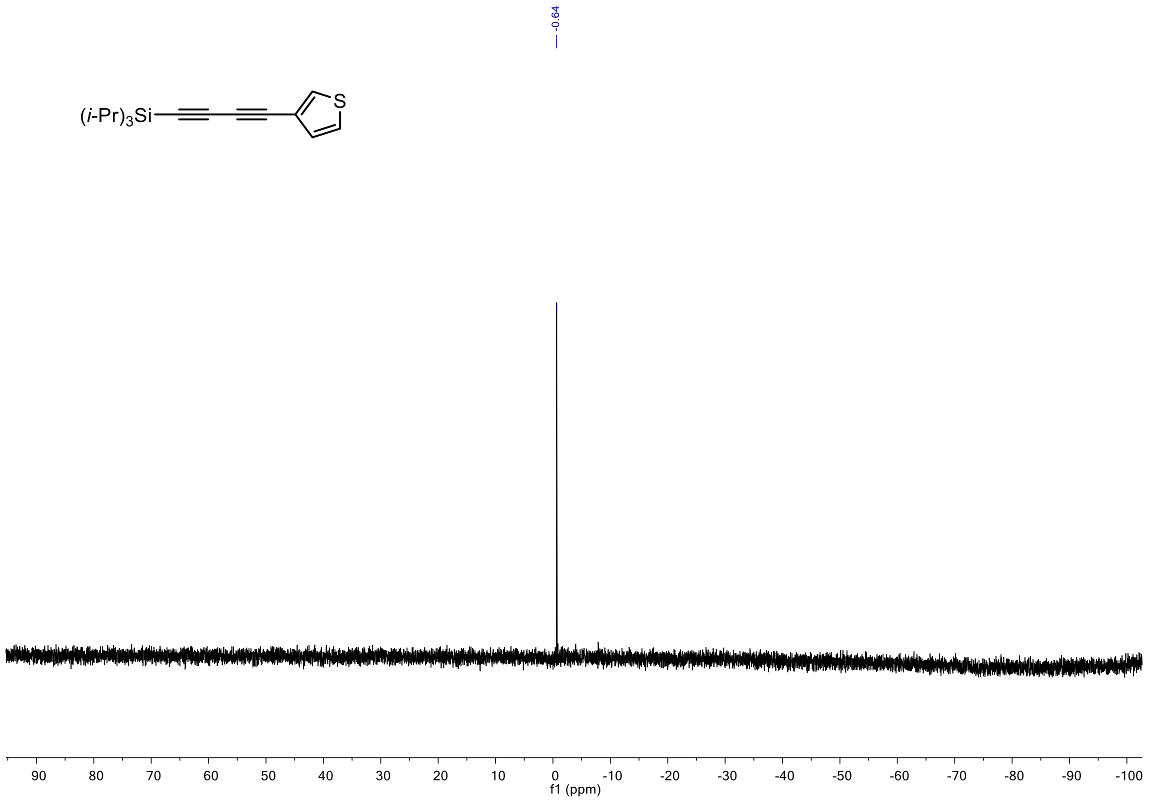

Figure S30. ^{29}Si NMR spectrum of **2j**.

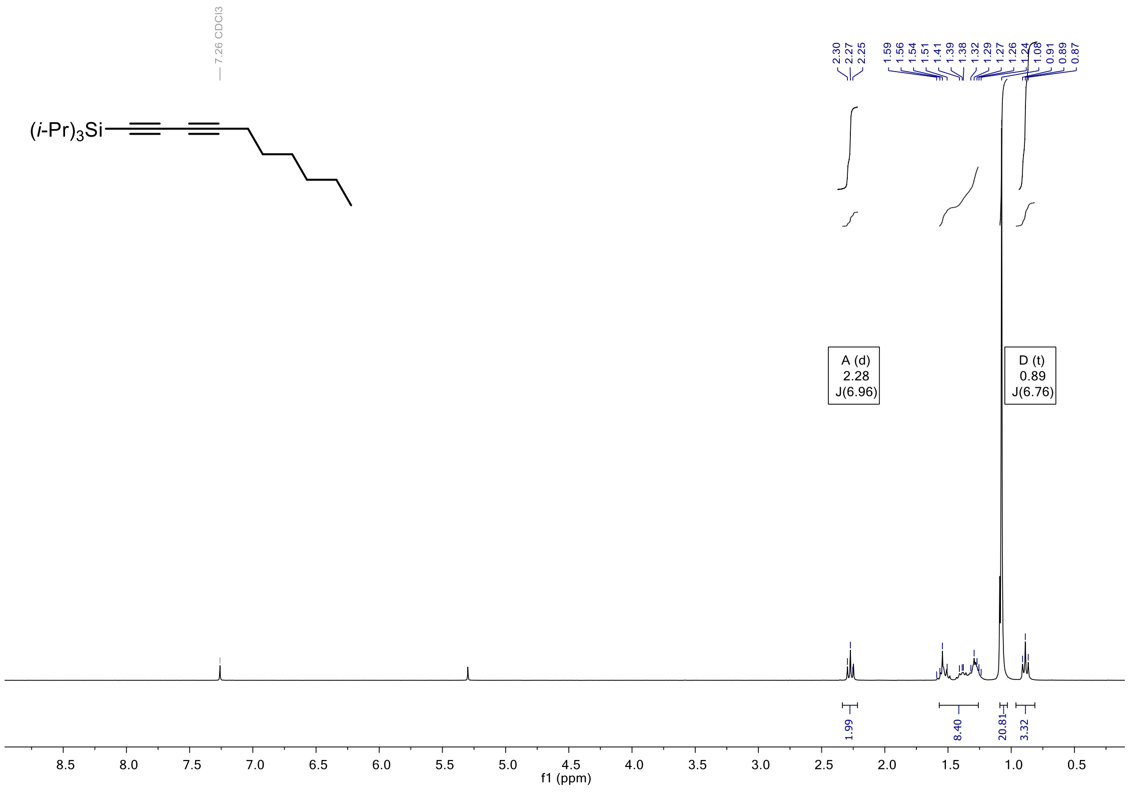

Figure S31. ^{1}H NMR spectrum of **2k**.

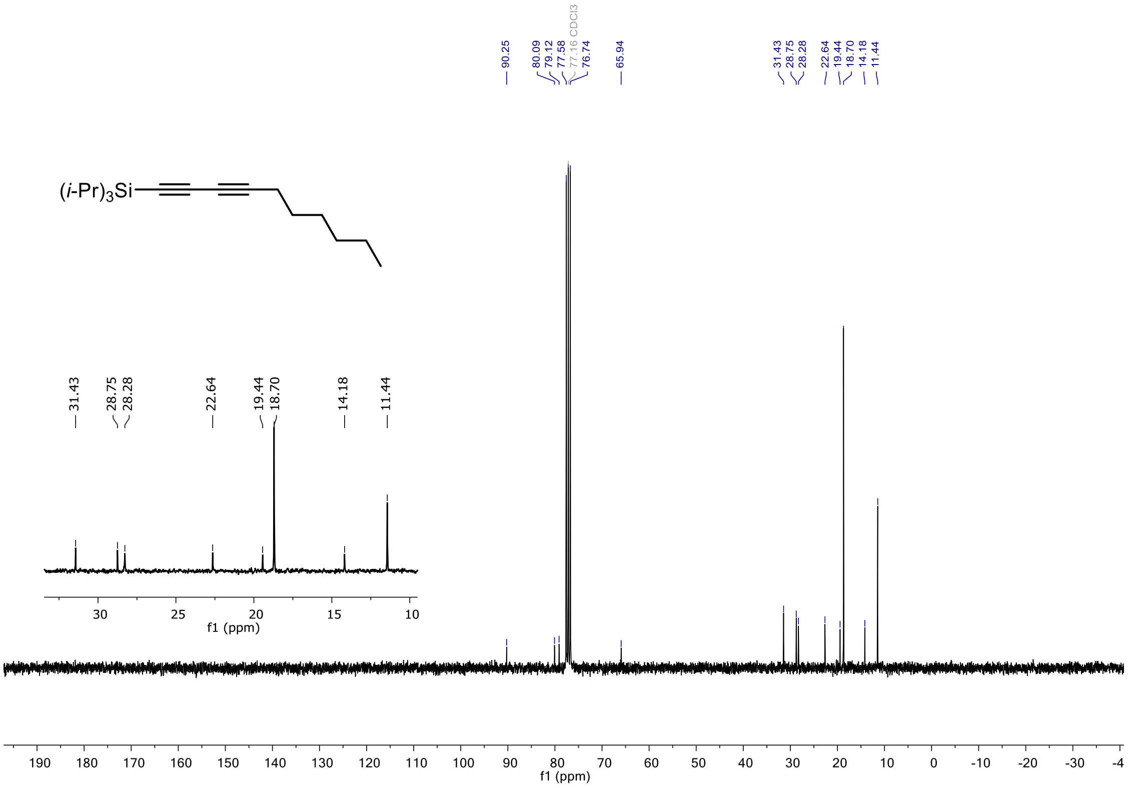

Figure S32. ^{13}C NMR spectrum of **2k**.

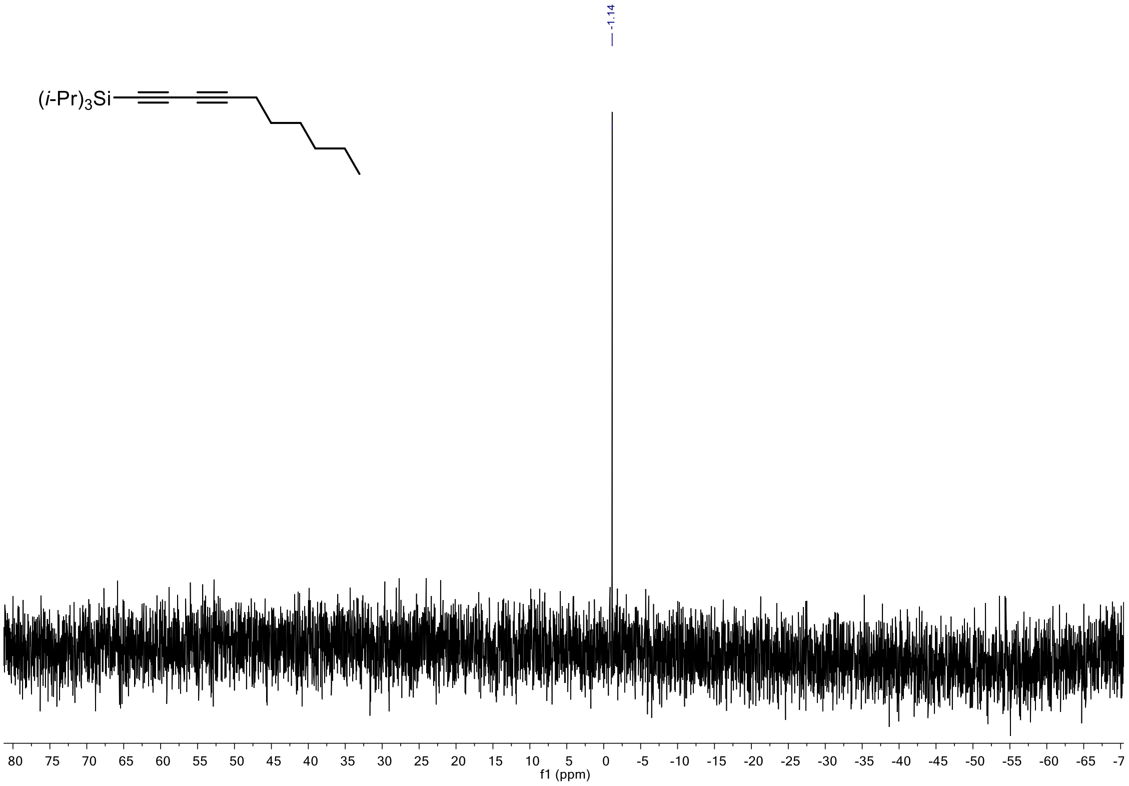

Figure S33. ^{29}Si NMR spectrum of **2k**.

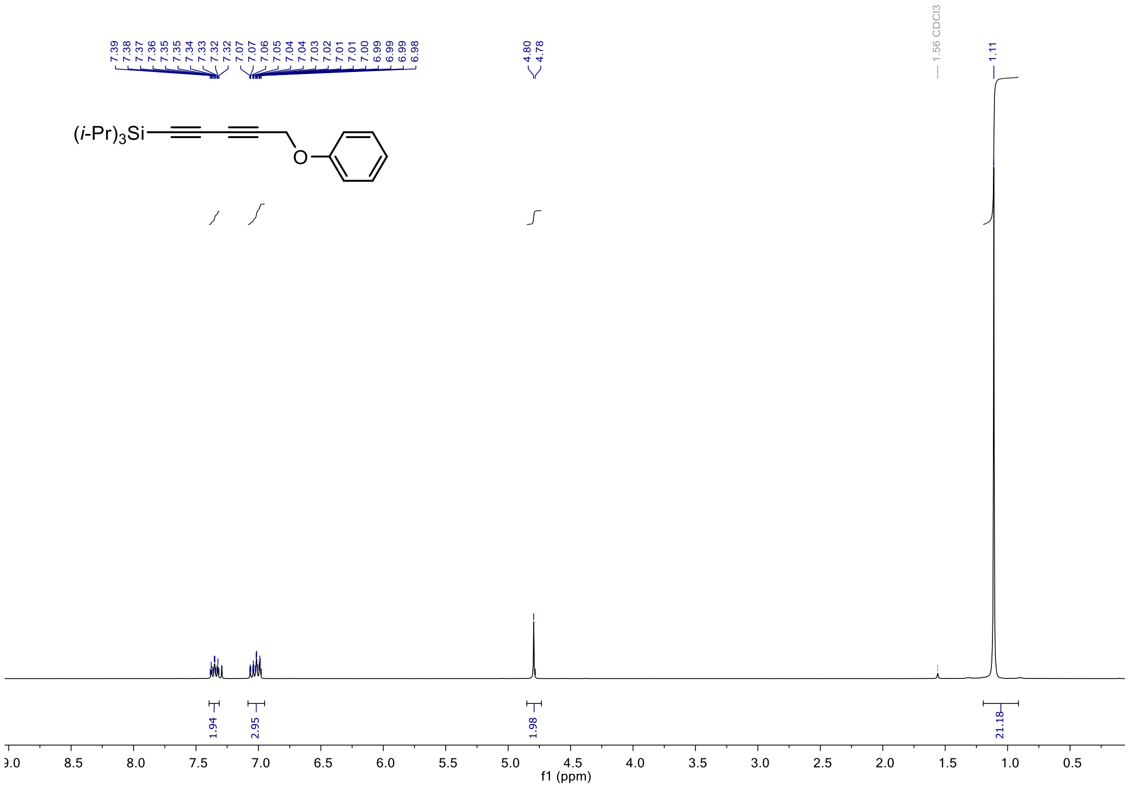

Figure S34. ^{1}H NMR spectrum of **2l**.

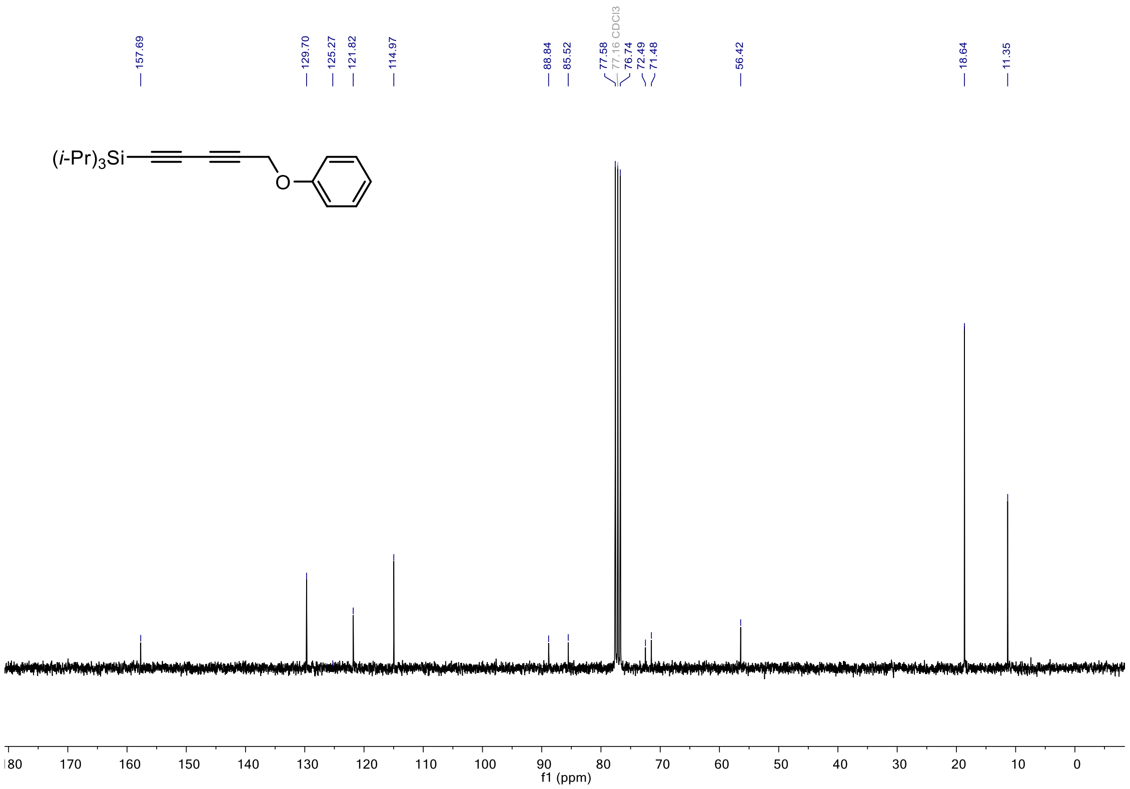

Figure S35. ^{13}C NMR spectrum of **2l**.

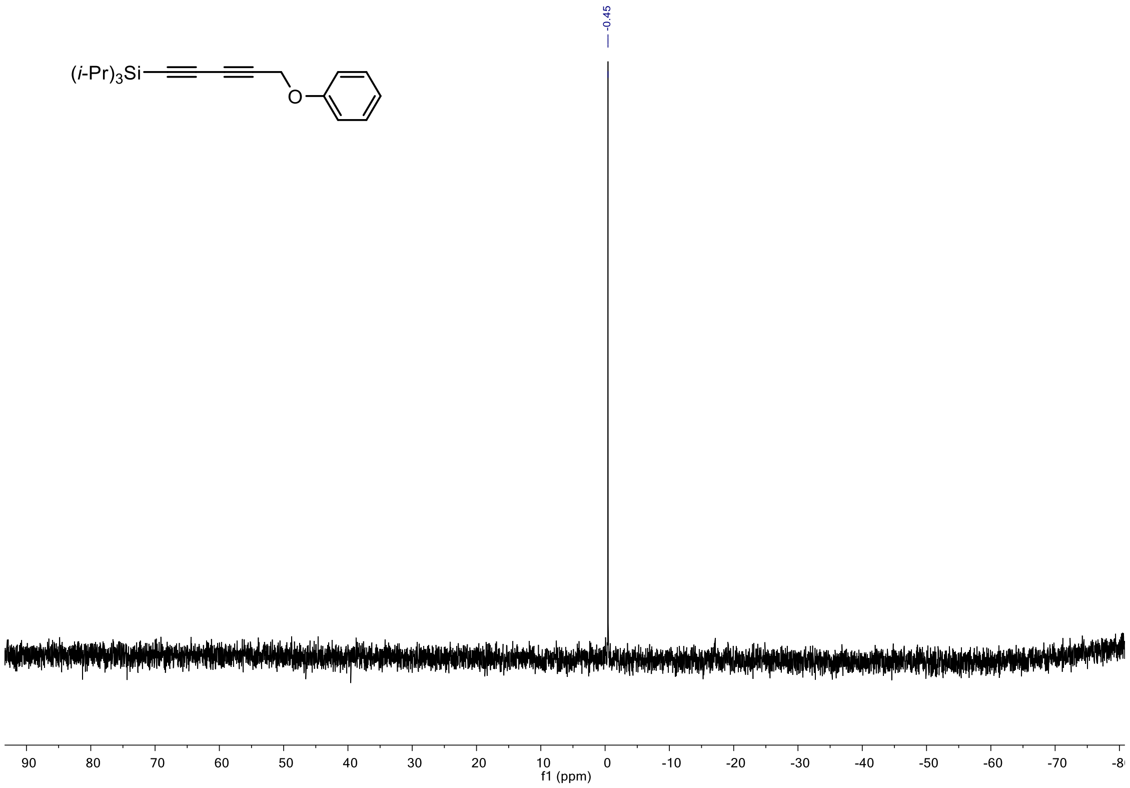

Figure S36. ^{29}Si NMR spectrum of **2l**.

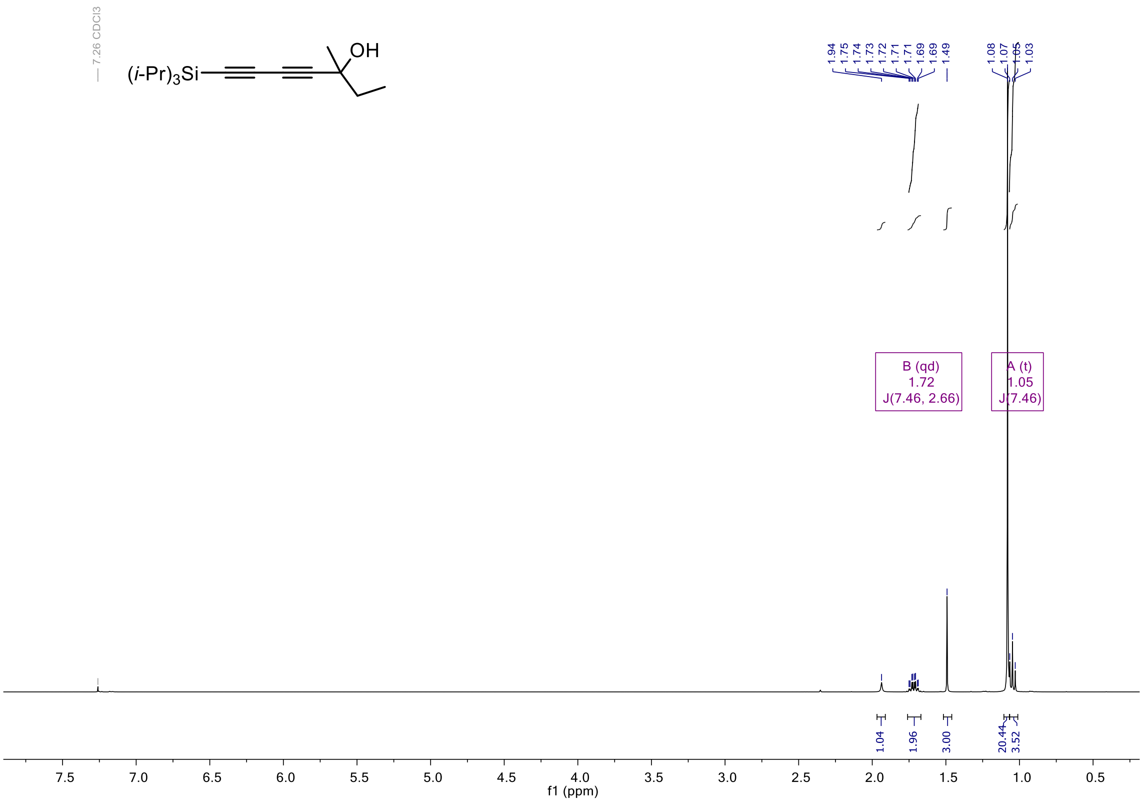

Figure S37. ^{1}H NMR spectrum of **2m**.

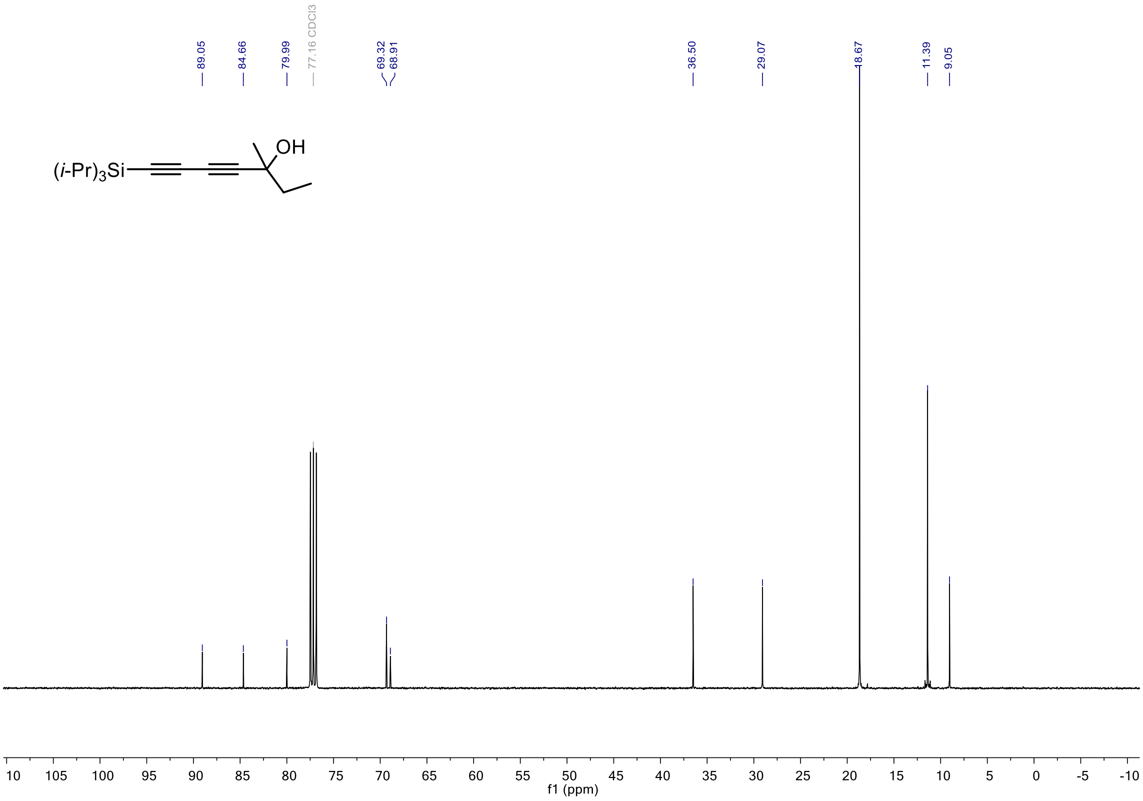

Figure S38. ^{13}C NMR spectrum of **2m**.

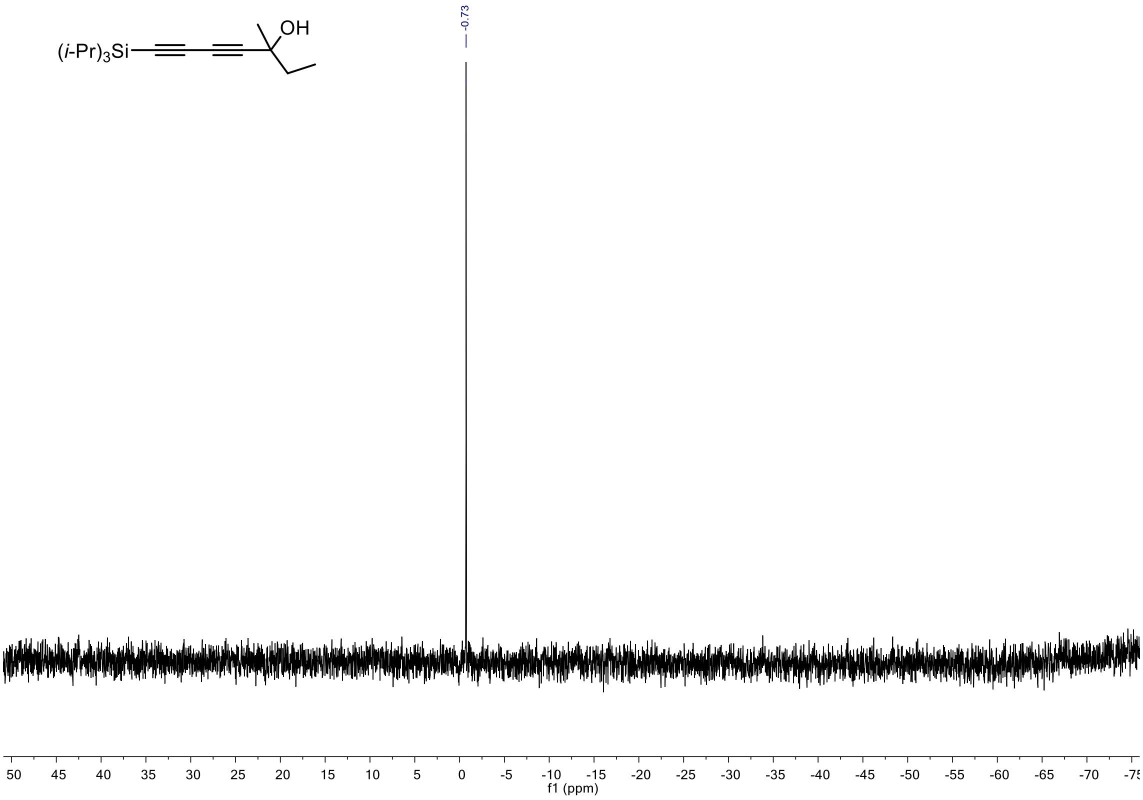

Figure S39. ^{29}Si NMR spectrum of **2m**.

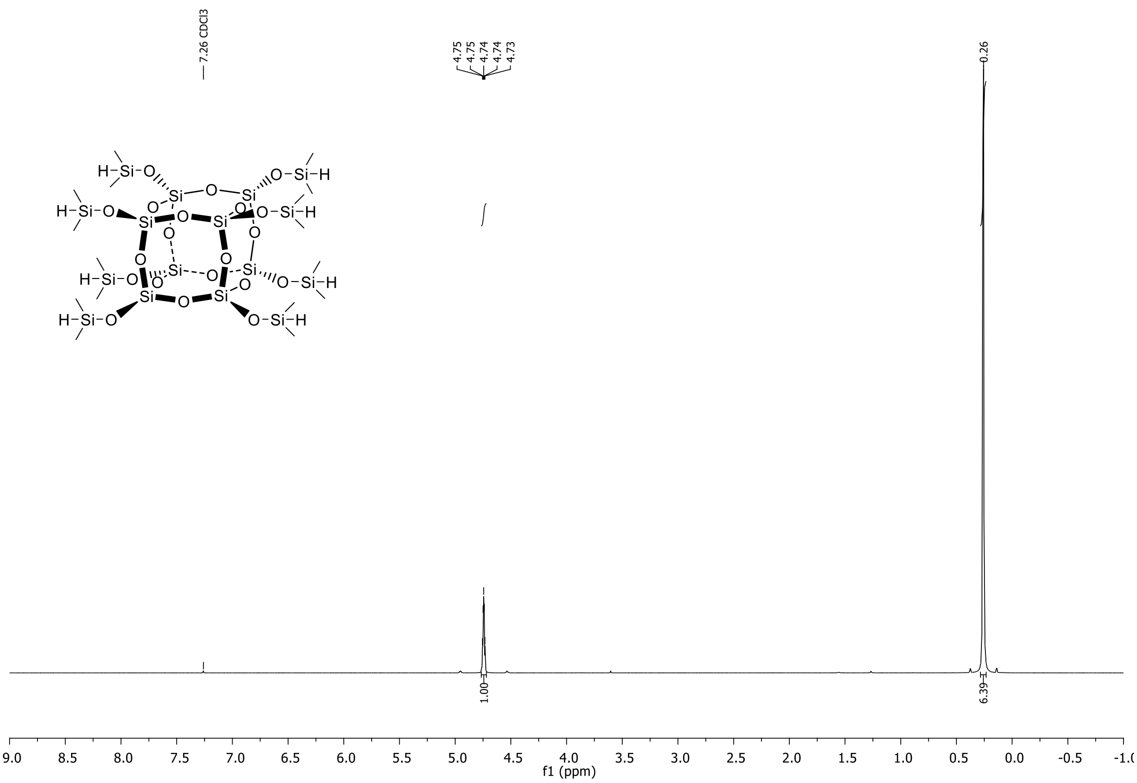

Figure S40. ^{1}H NMR spectra of compound **1**.

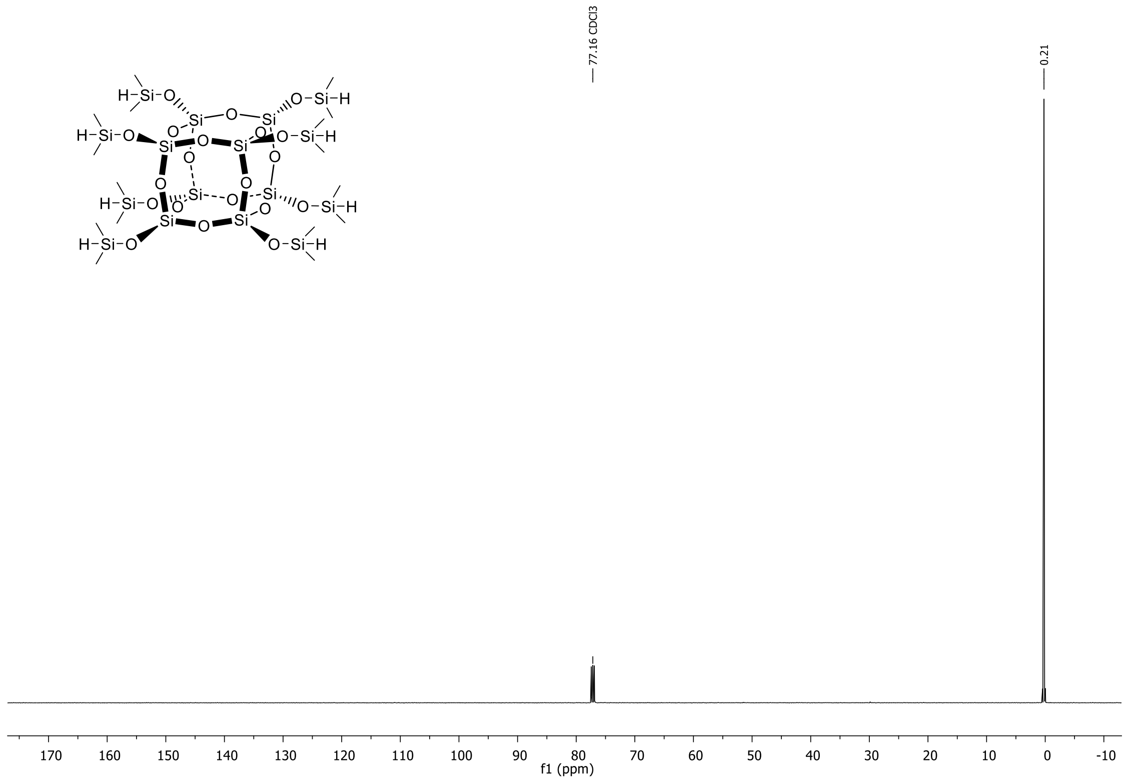

Figure S41. ^{13}C NMR spectra of compound **1**.

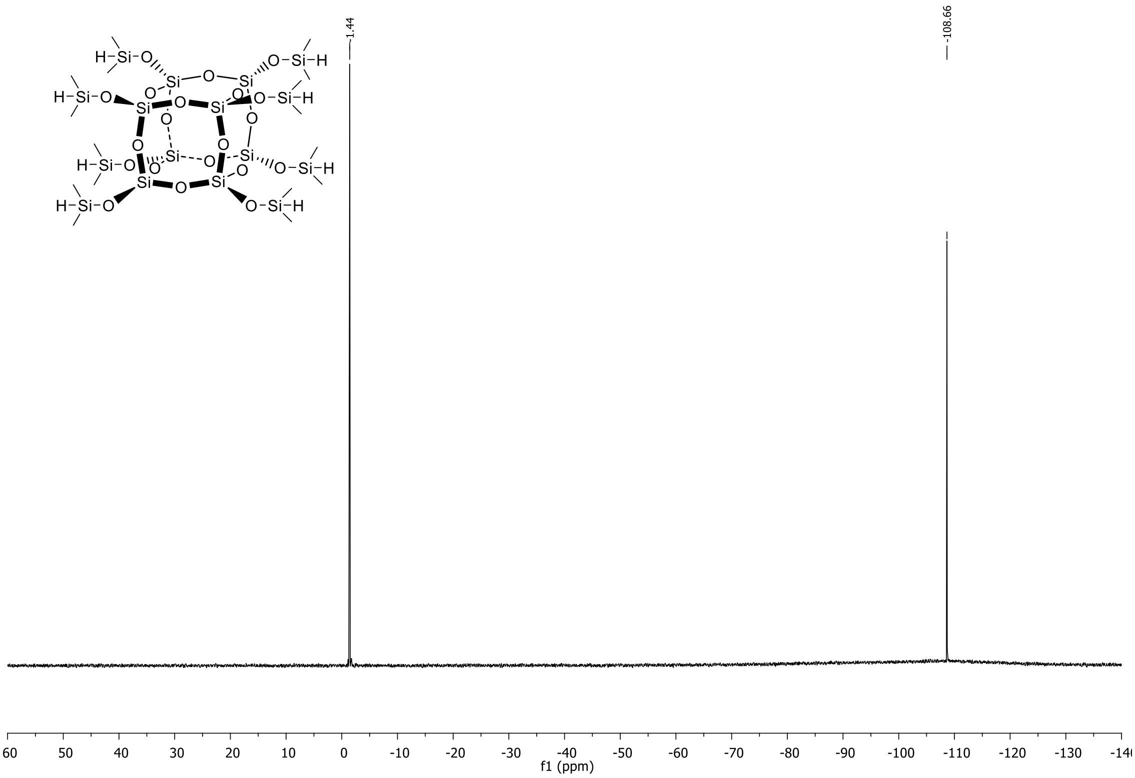

Figure S42. ^{29}Si NMR spectra of compound **1**.

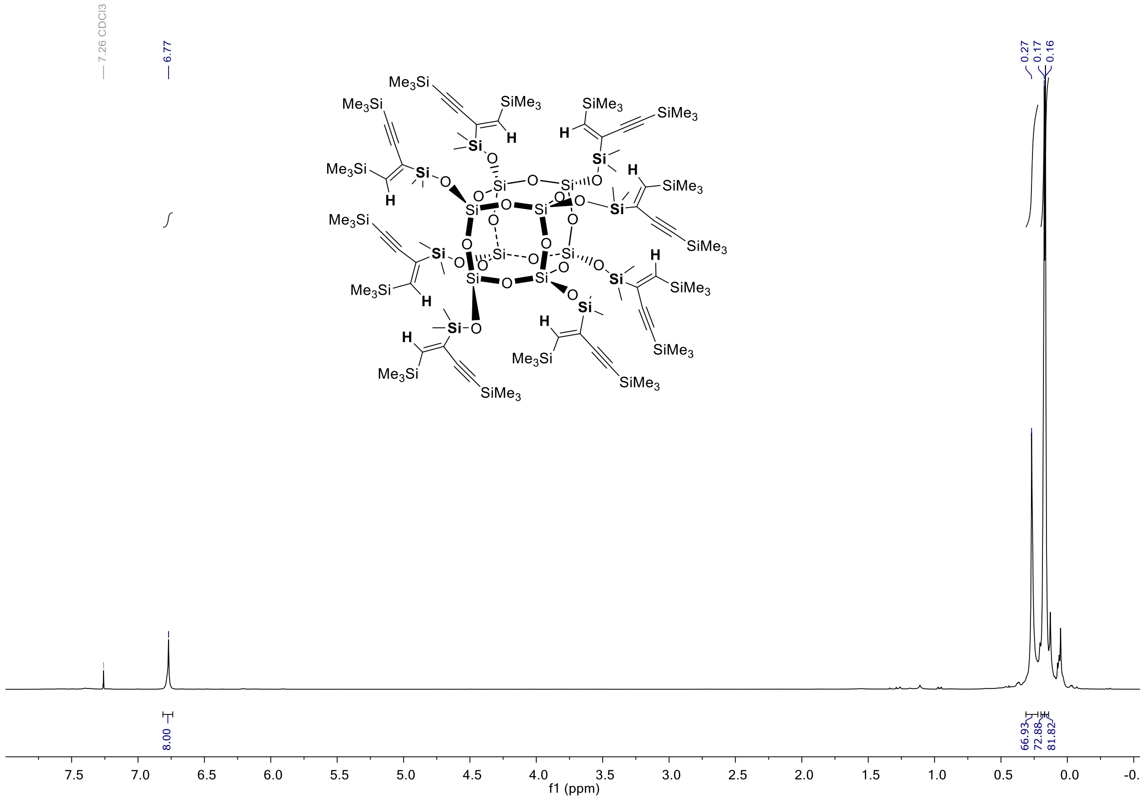

Figure S43. ^{1}H NMR of compound **3a**.

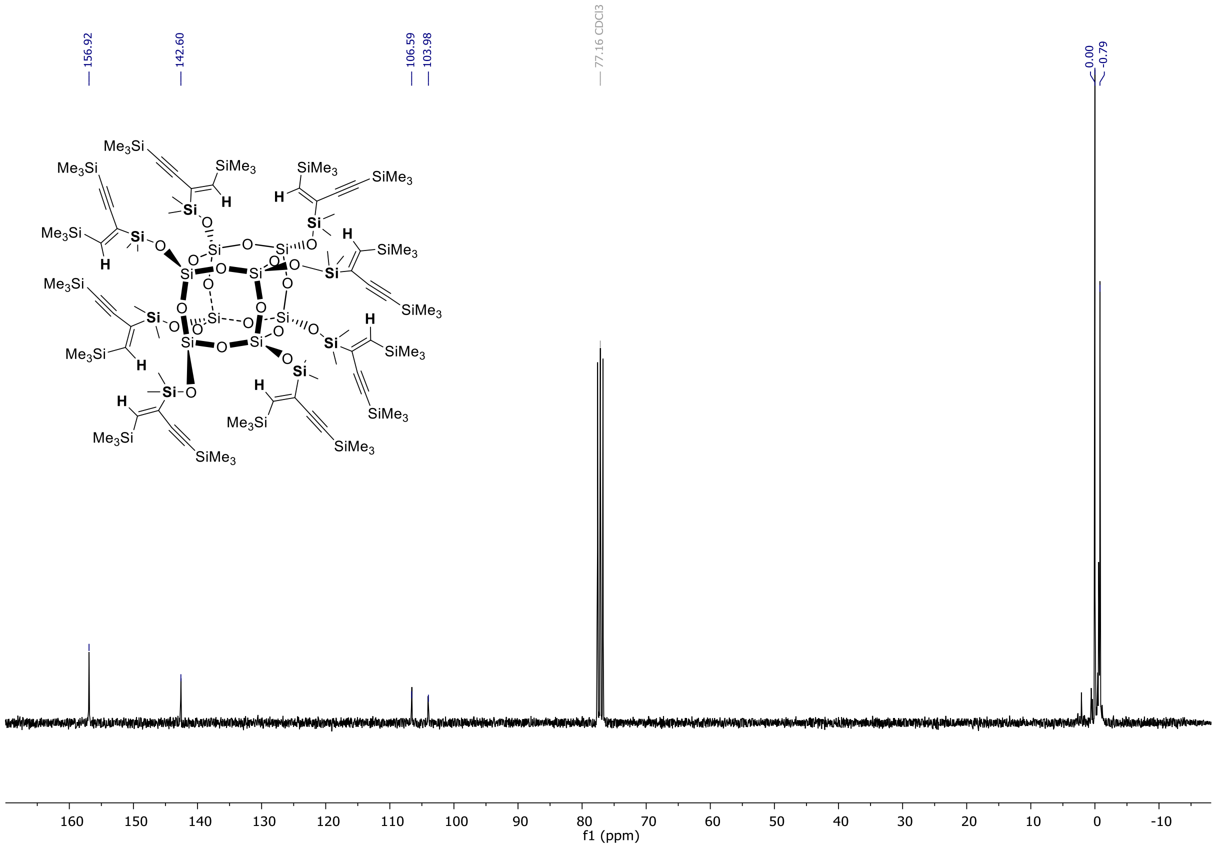

Figure S44. ^{13}C NMR of compound **3a**.

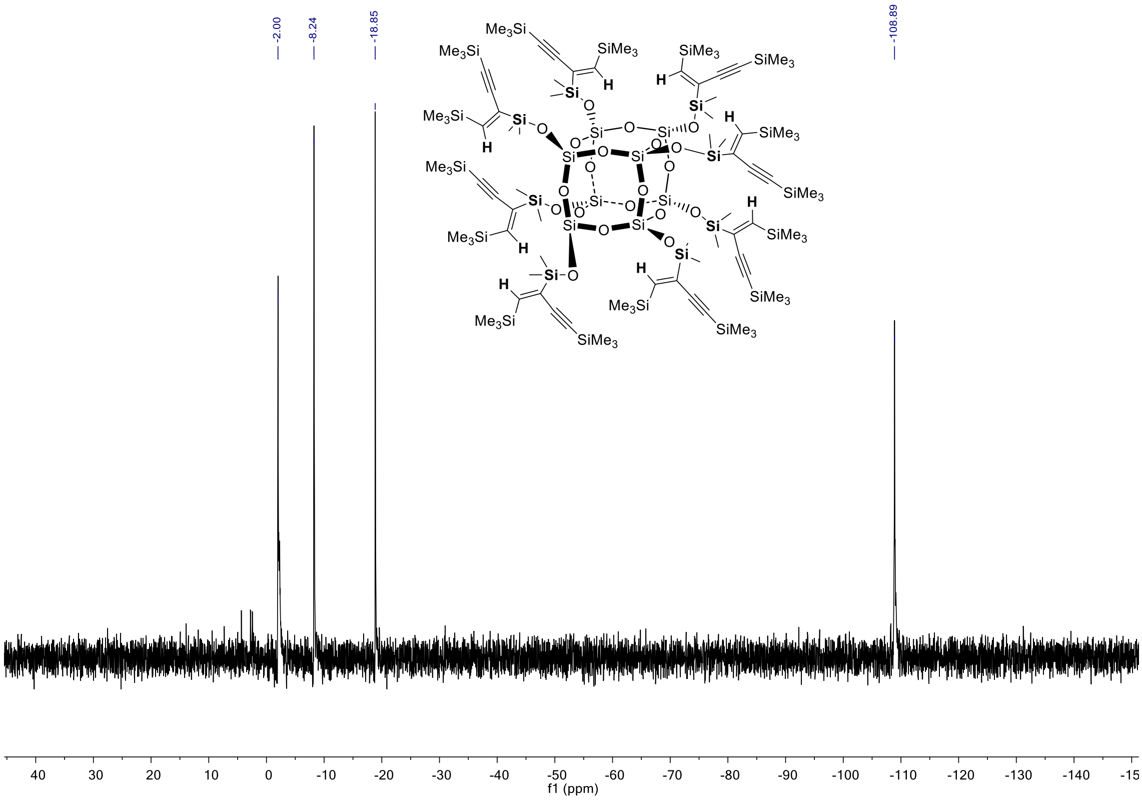

Figure S45. ^{29}Si NMR of compound **3a**.

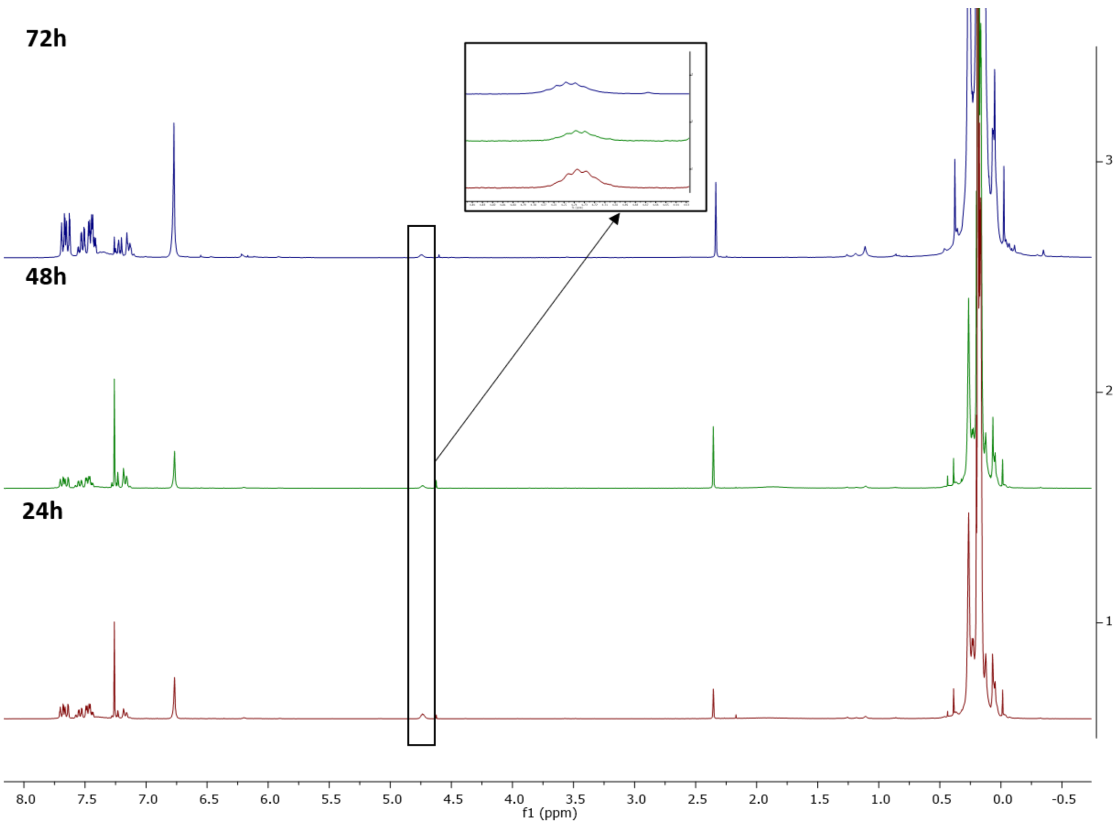

Figure S46. The progress of the synthesis of **3a** under optimized conditions after 24h, 48h and 72h.

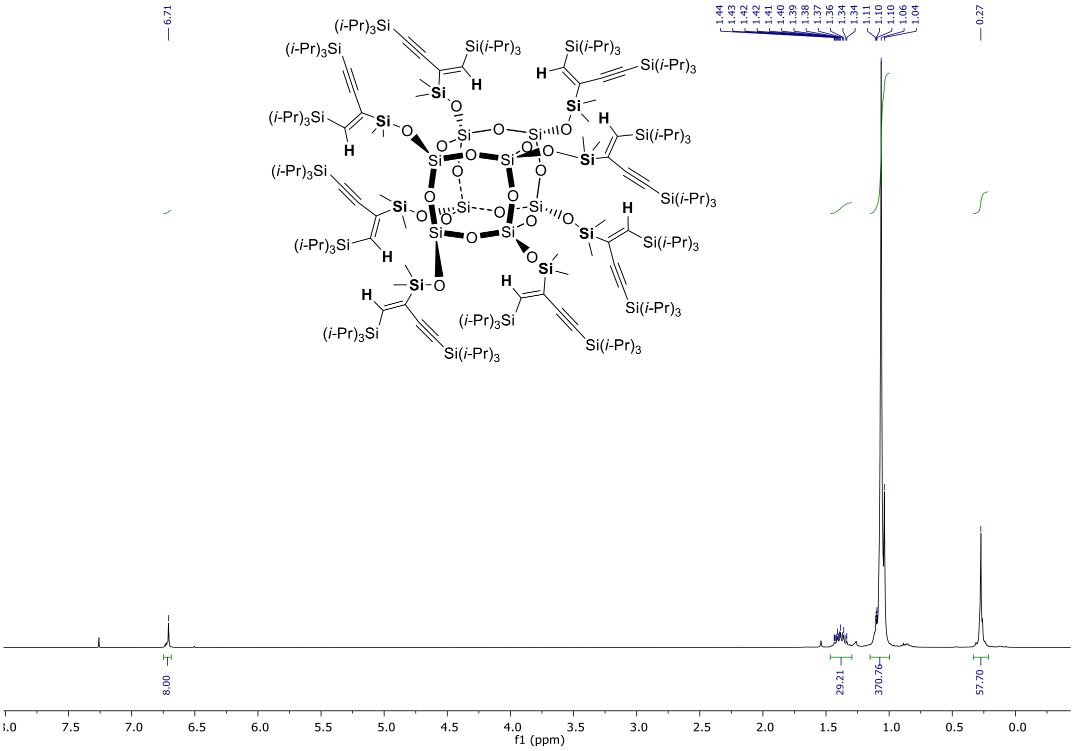

Figure S47. ^{1}H NMR of compound **3b**.

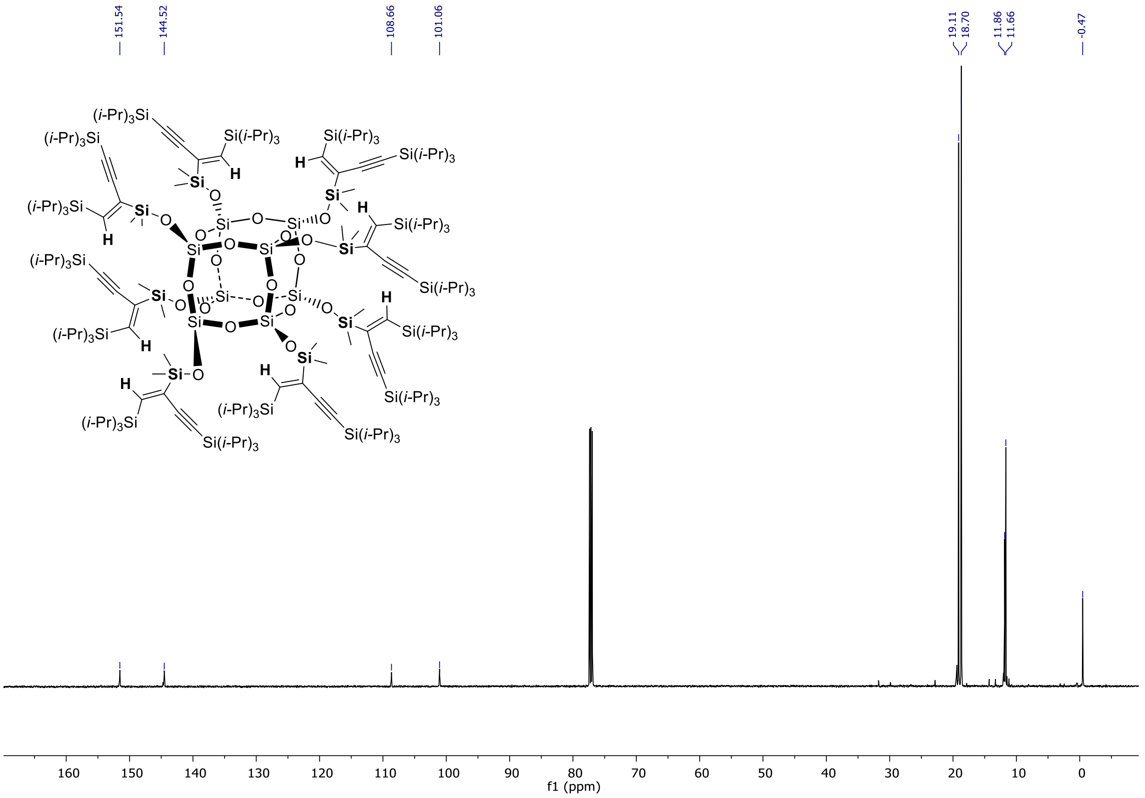

Figure S46. ^{13}C NMR of compound **3b**.

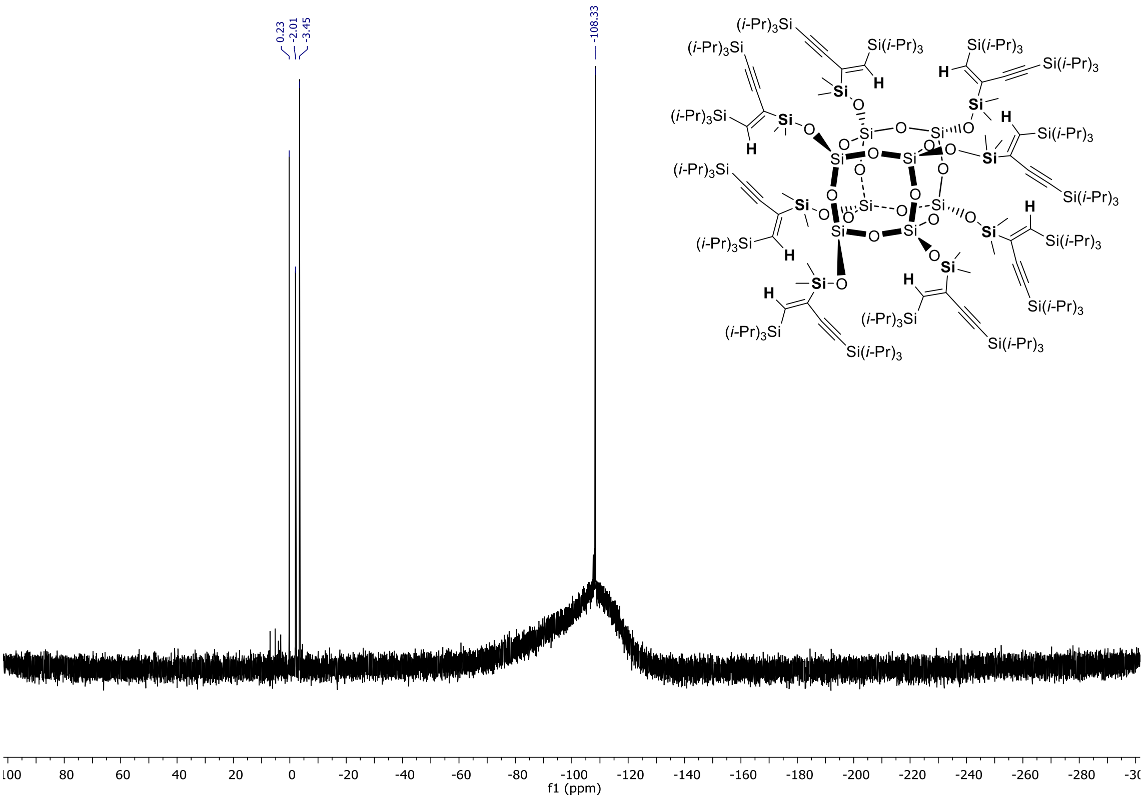

Figure S48. ^{29}Si NMR of compound **3b**.

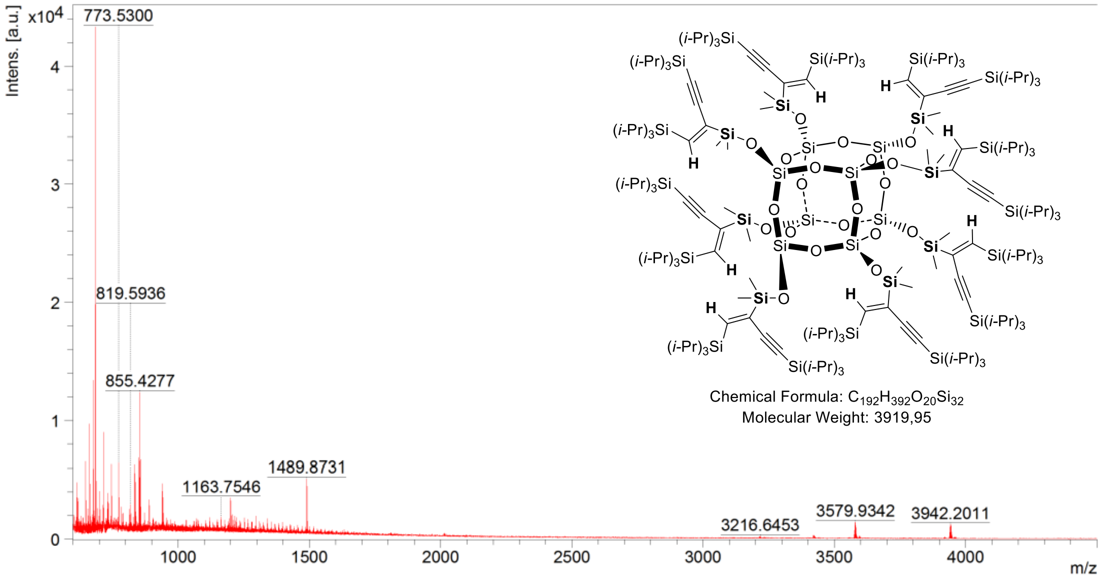

Figure S49. MALDI TOF MS spectra of compound **3b**.

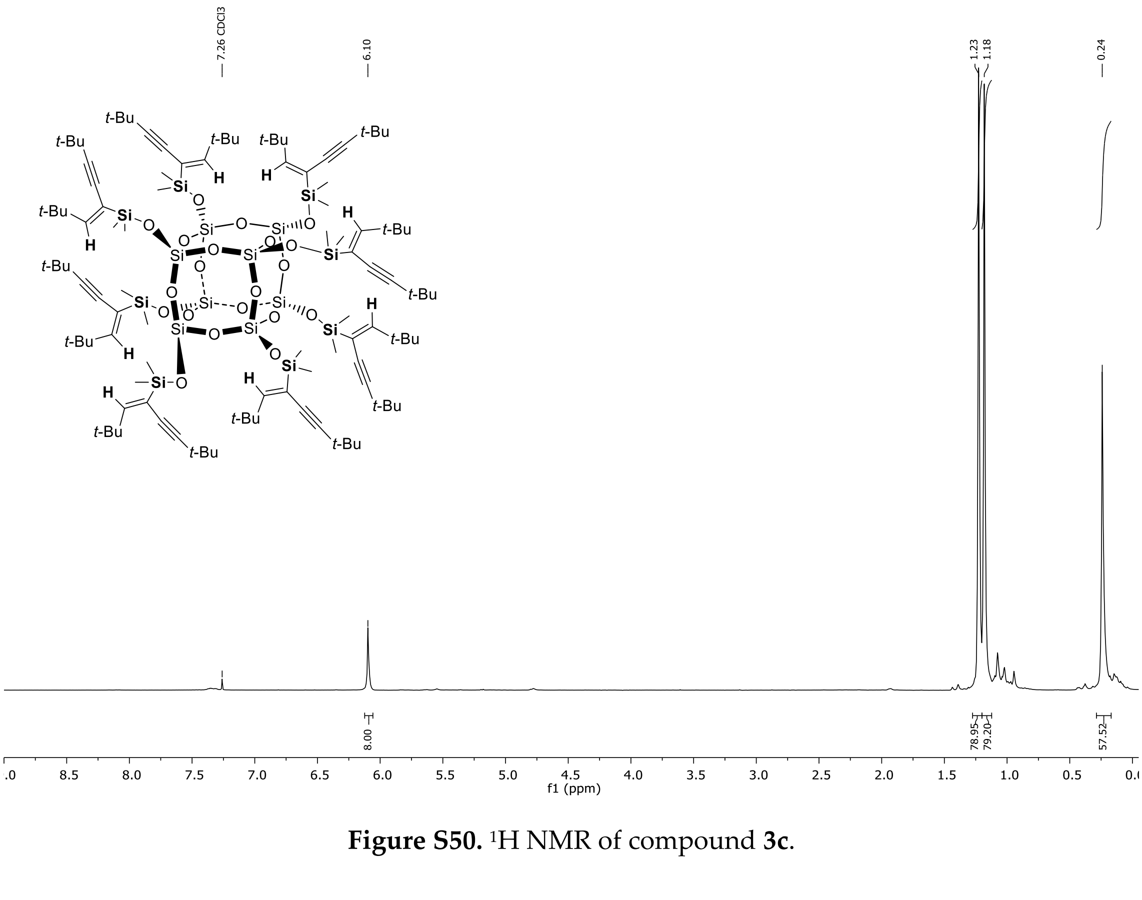

Figure S50. ¹H NMR of compound **3c**.

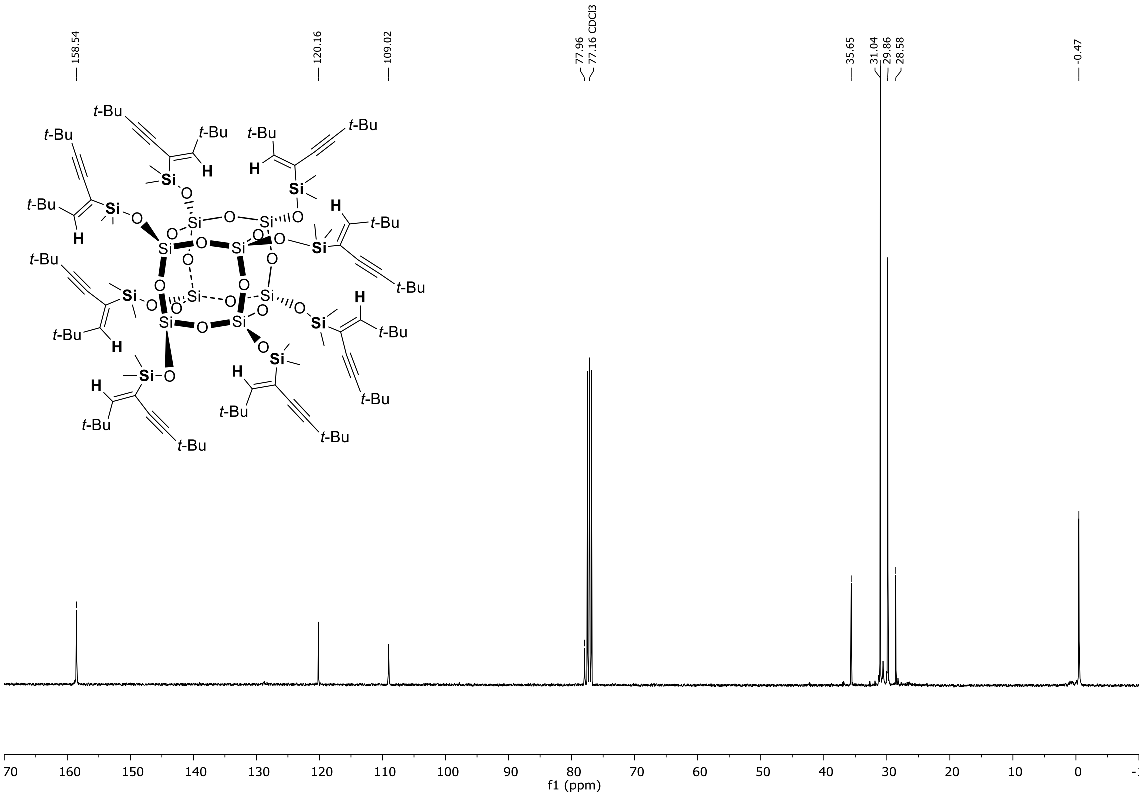

Figure S51. ^{13}C NMR of compound **3c**.

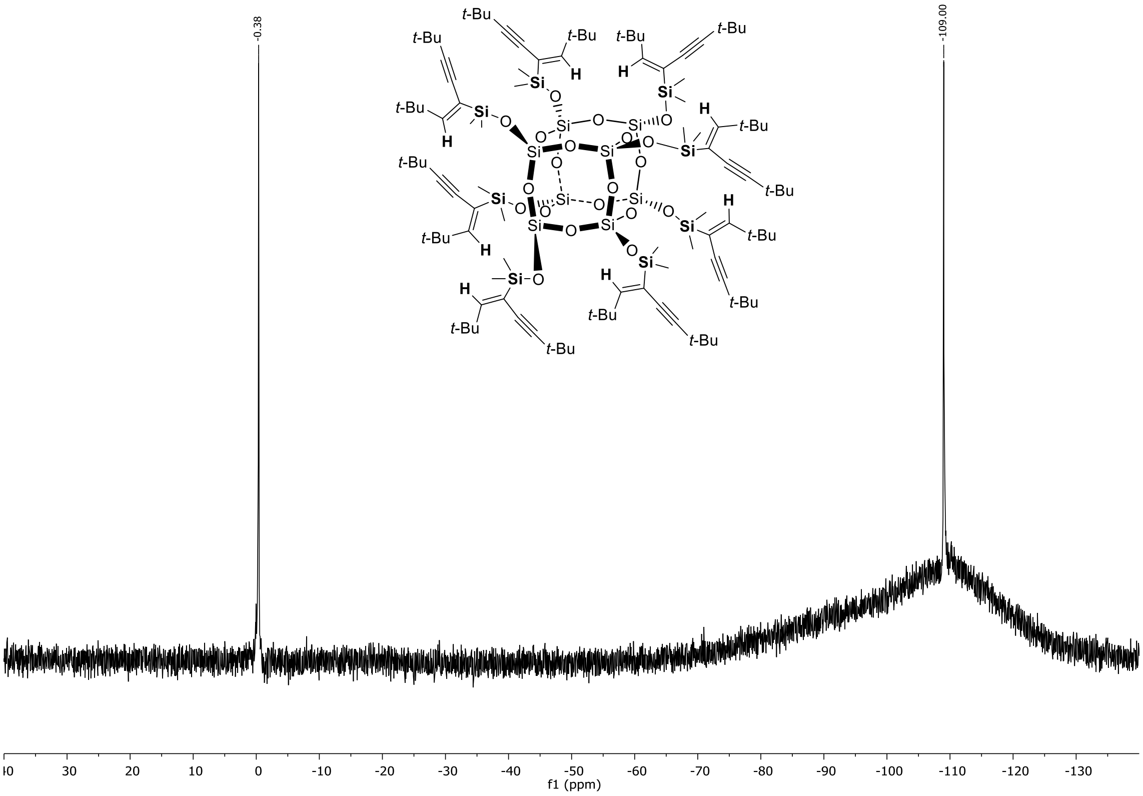

Figure S52. ^{29}Si NMR of compound **3c**.

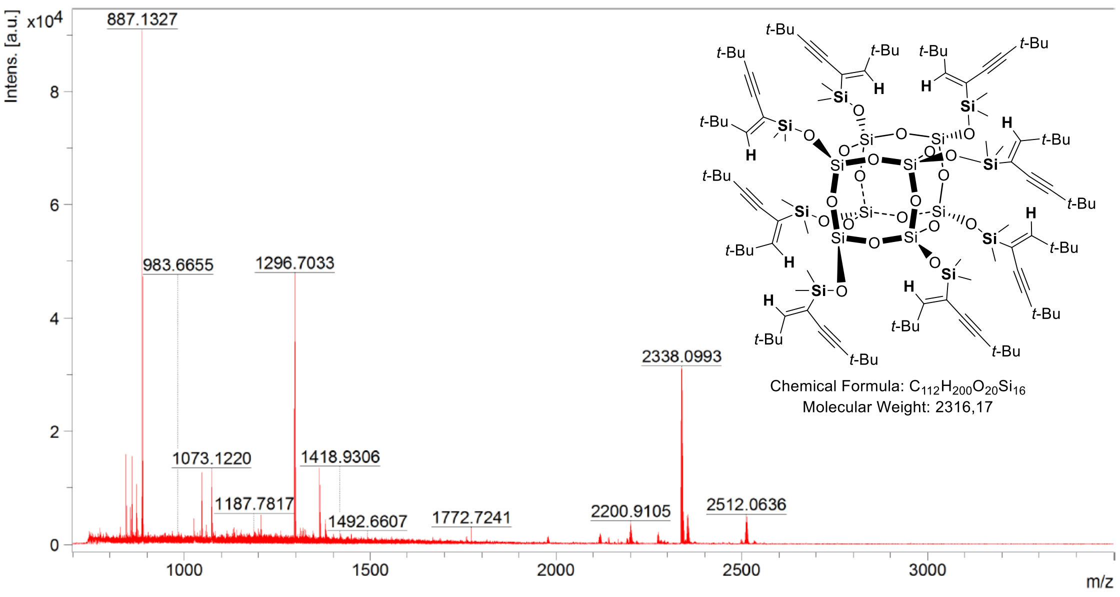

Figure S53. MALDI TOF MS spectra of compound **3c**.

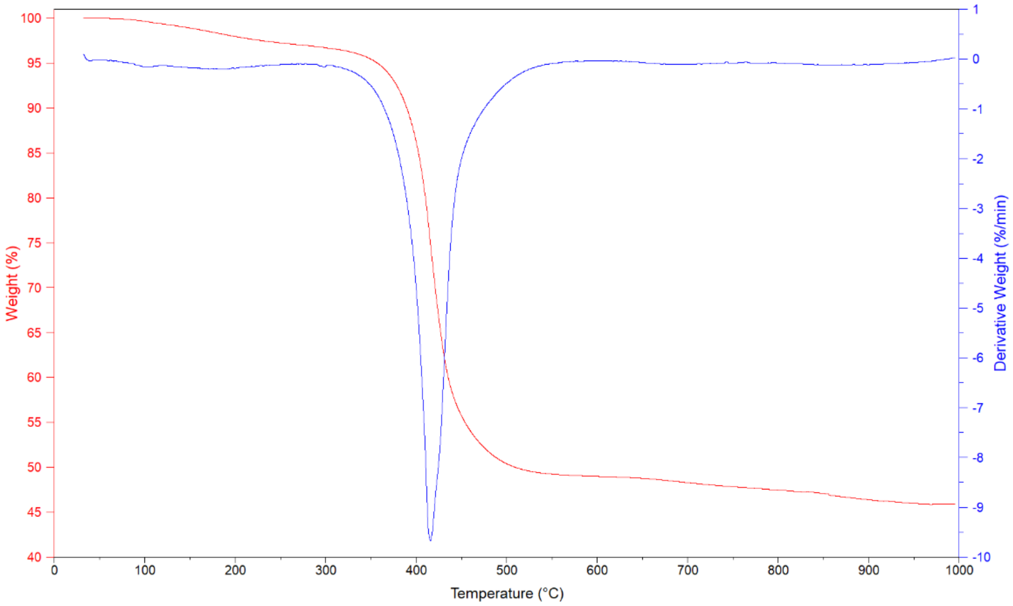

Figure S54. TGA/DTG curves of compound **3c**.

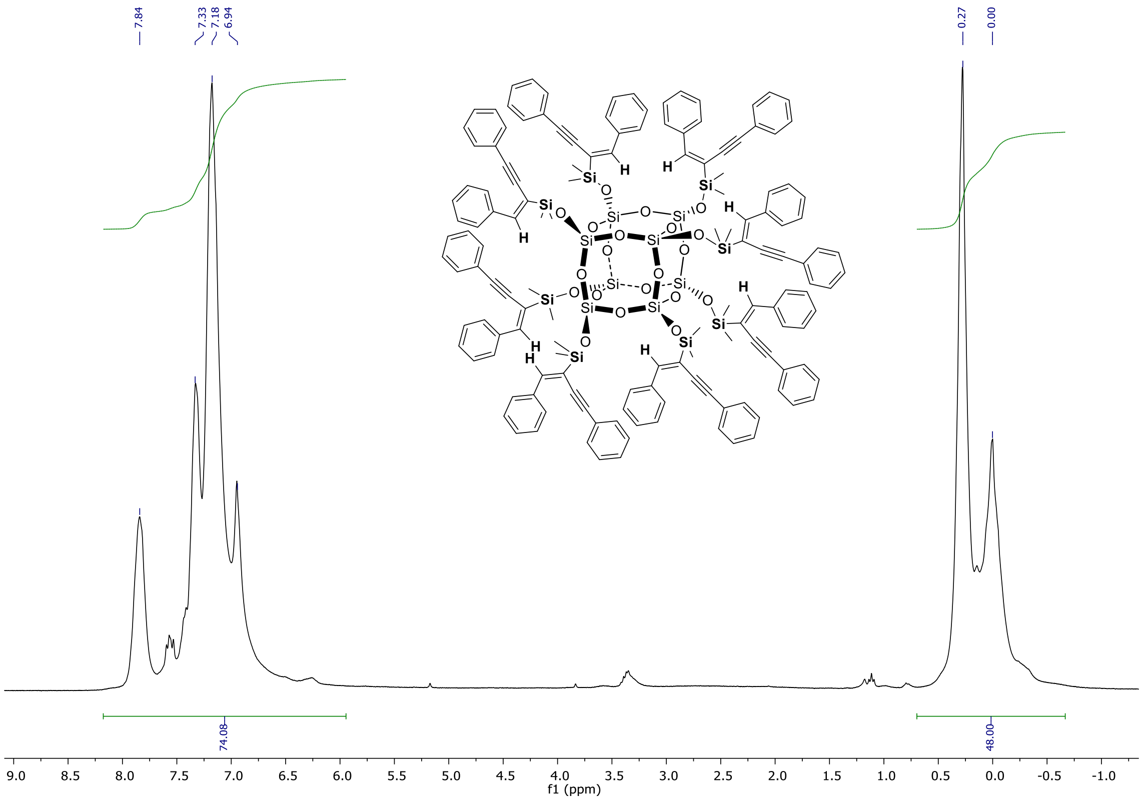

Figure S55. ^{1}H NMR of compound **3d**.

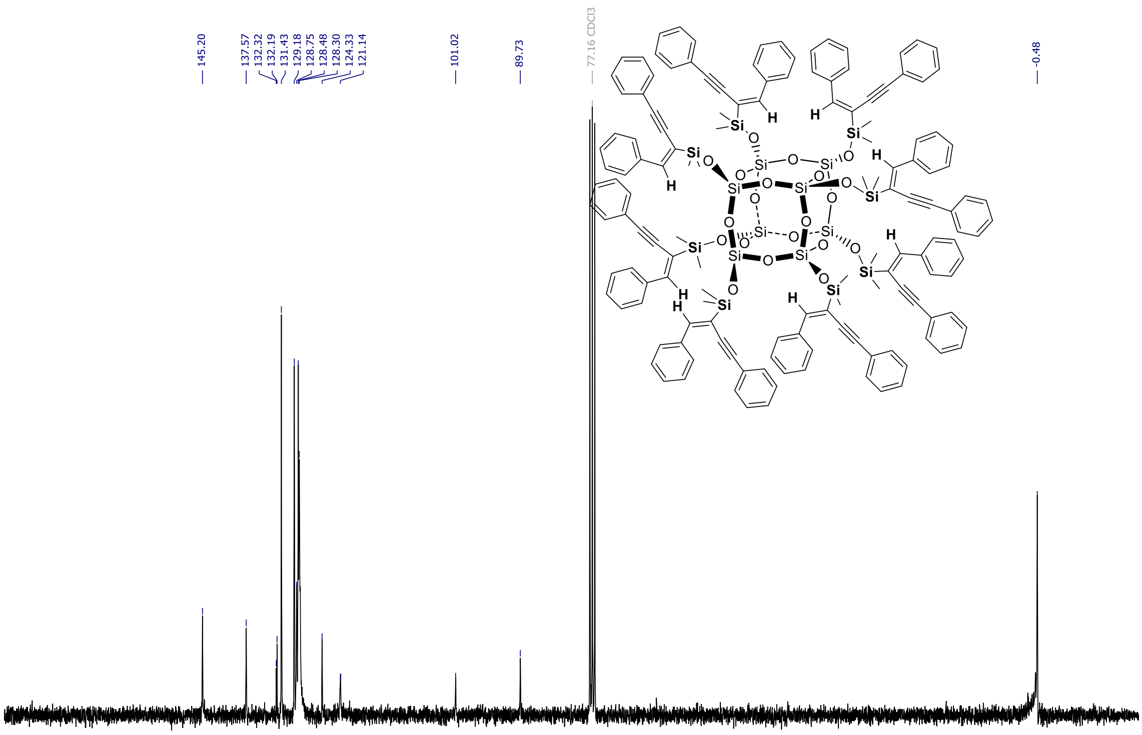

Figure S56. ^{13}C NMR of compound **3d**.

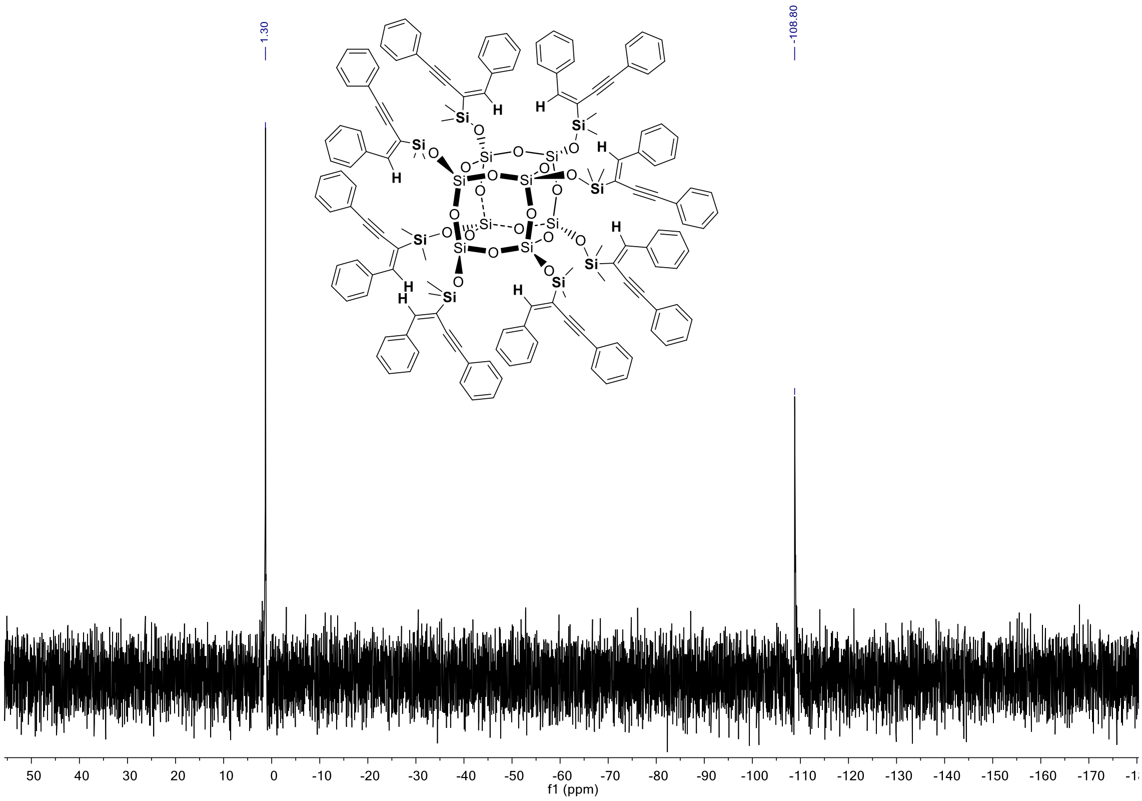

Figure S57. ^{29}Si NMR of compound **3d**.

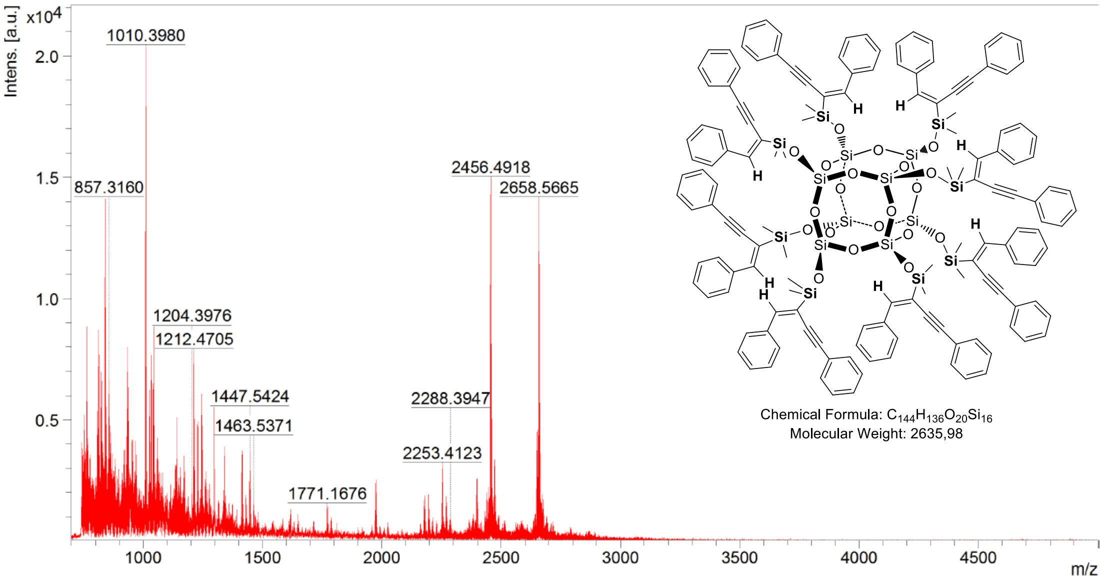

Figure S58. MALDI TOF MS spectra of compound **3d**.

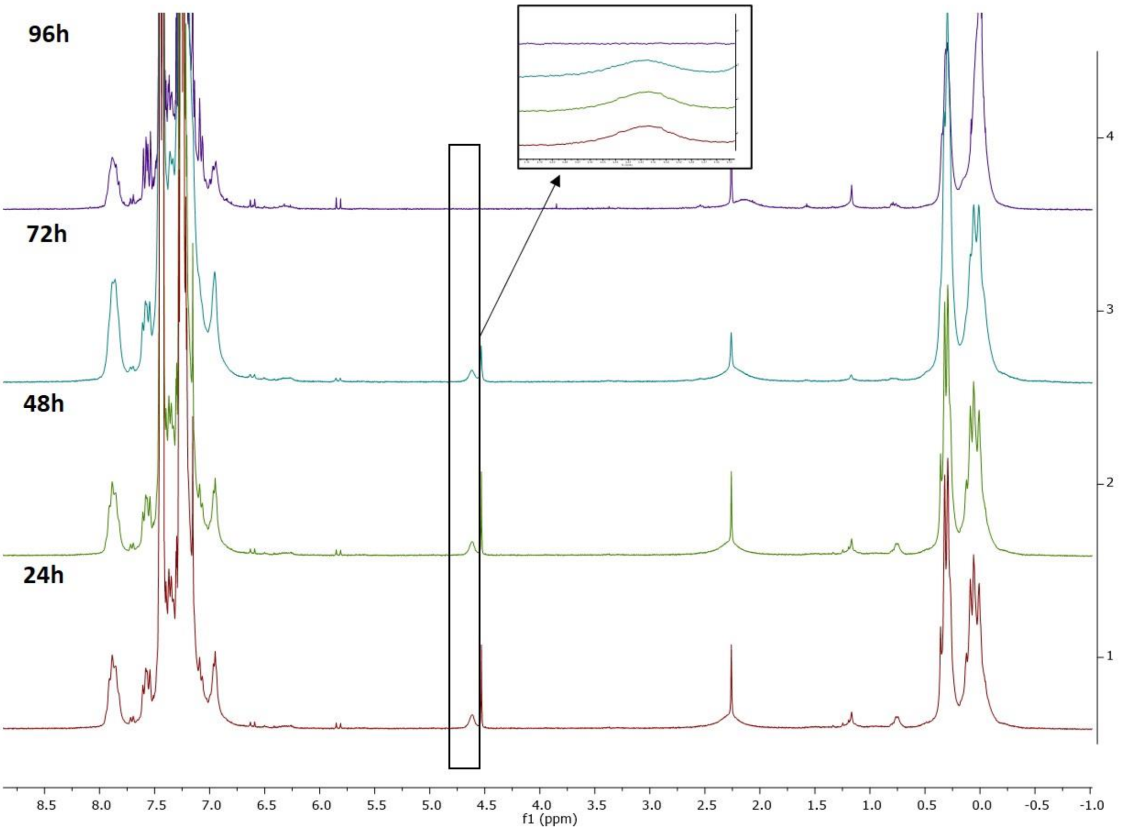

Figure S59. The progress of the synthesis of **3d** under optimized conditions after 24h, 48h, 72h and 96h.

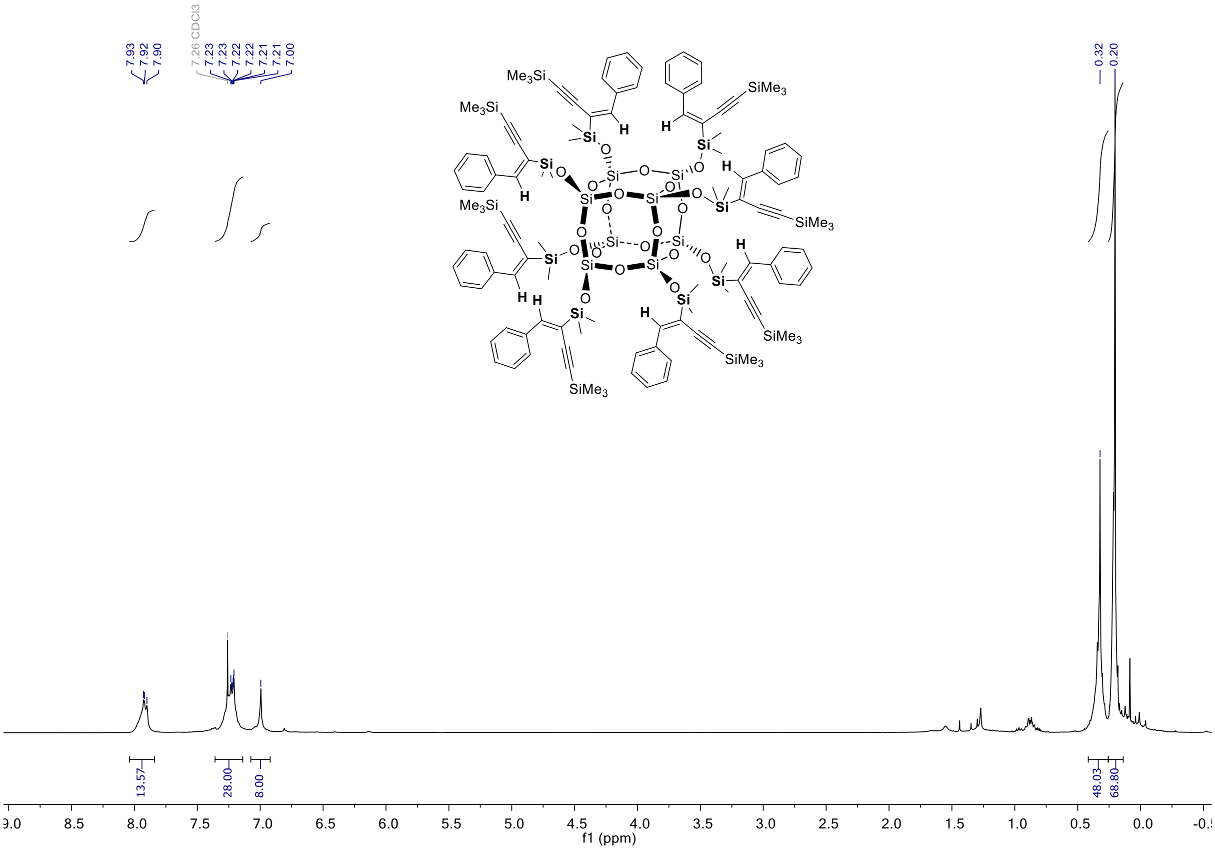

Figure S60. ^{1}H NMR of compound **3e**.

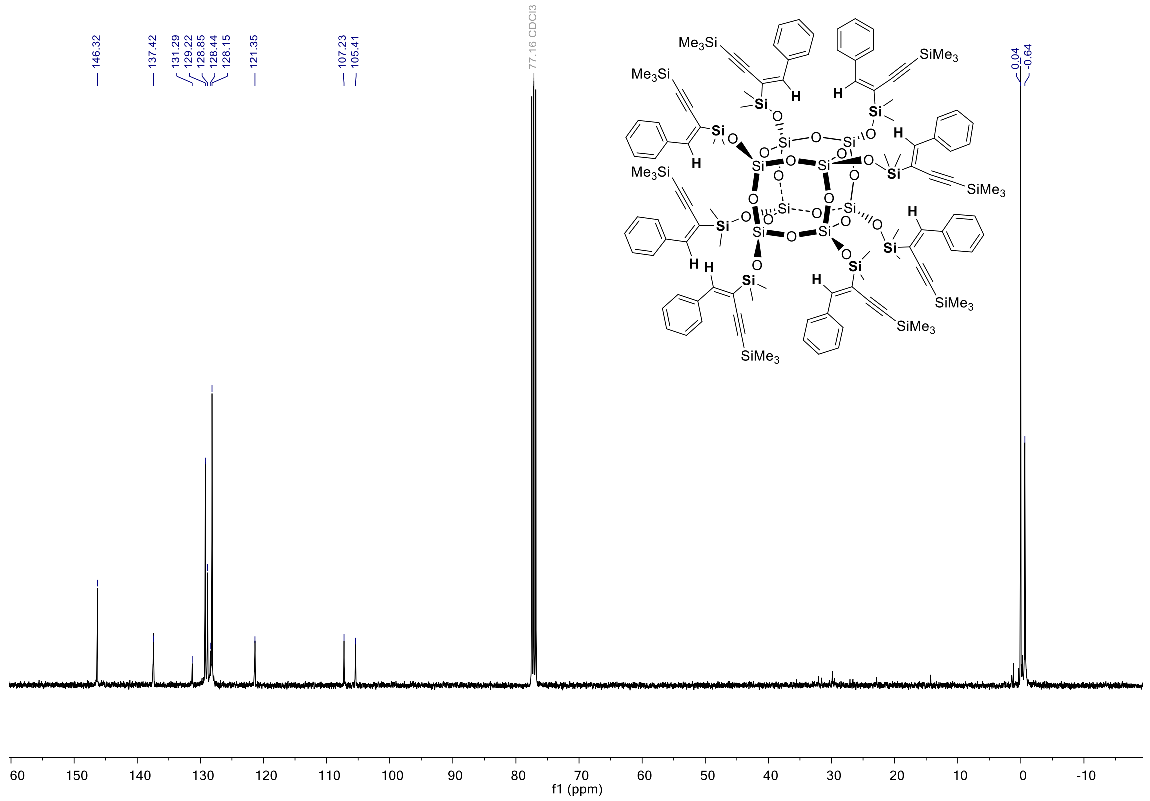

Figure S61. ^{13}C NMR of compound **3e**.

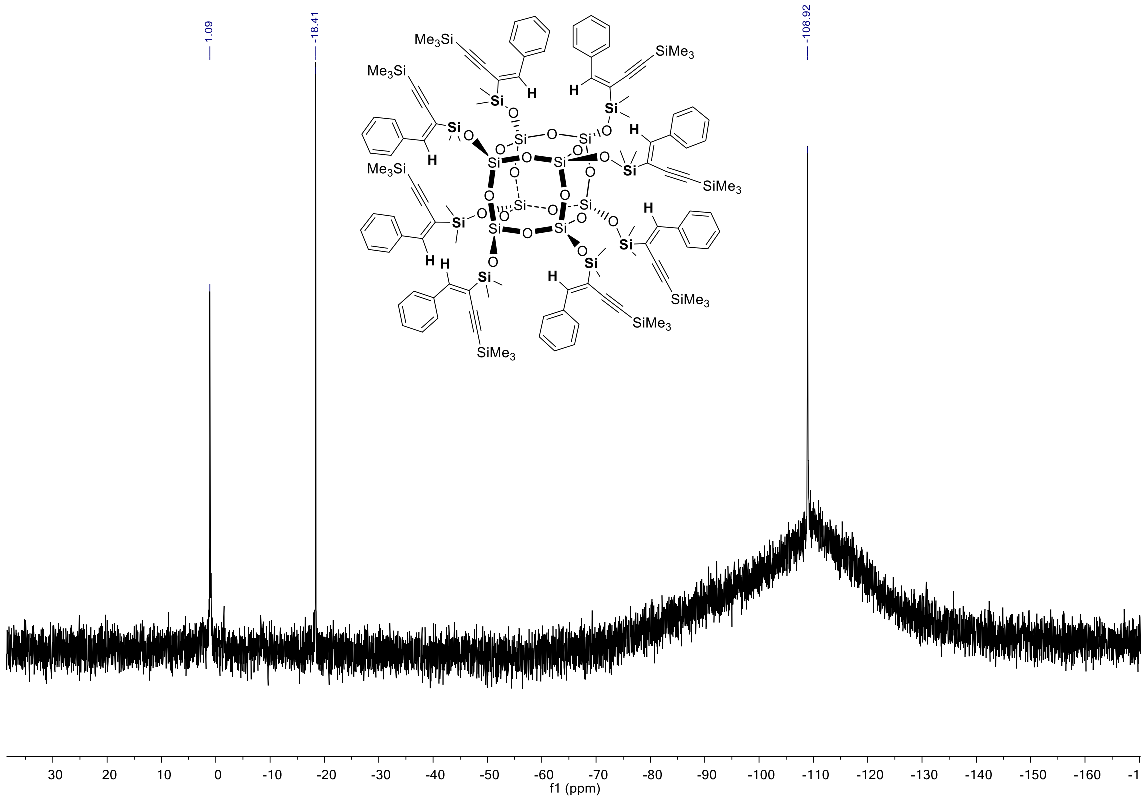

Figure S62. ^{29}Si NMR of compound **3e**.

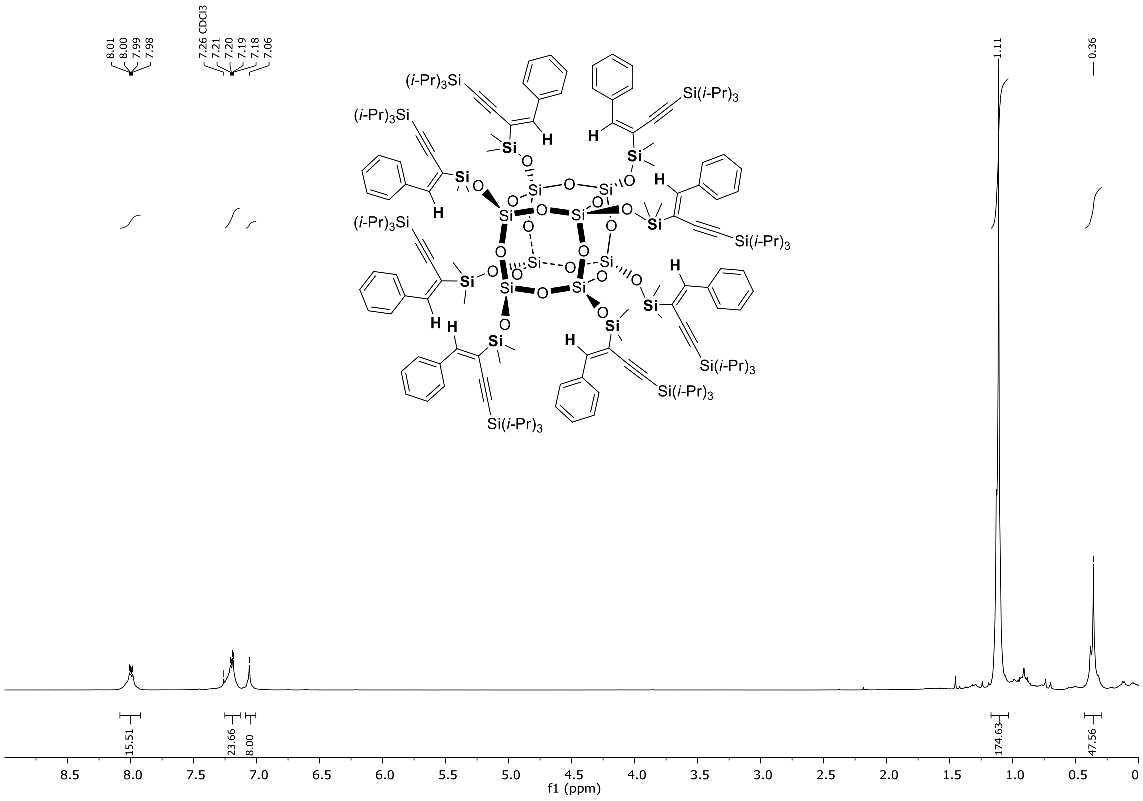

Figure S63. ^{1}H NMR of compound **3f**.

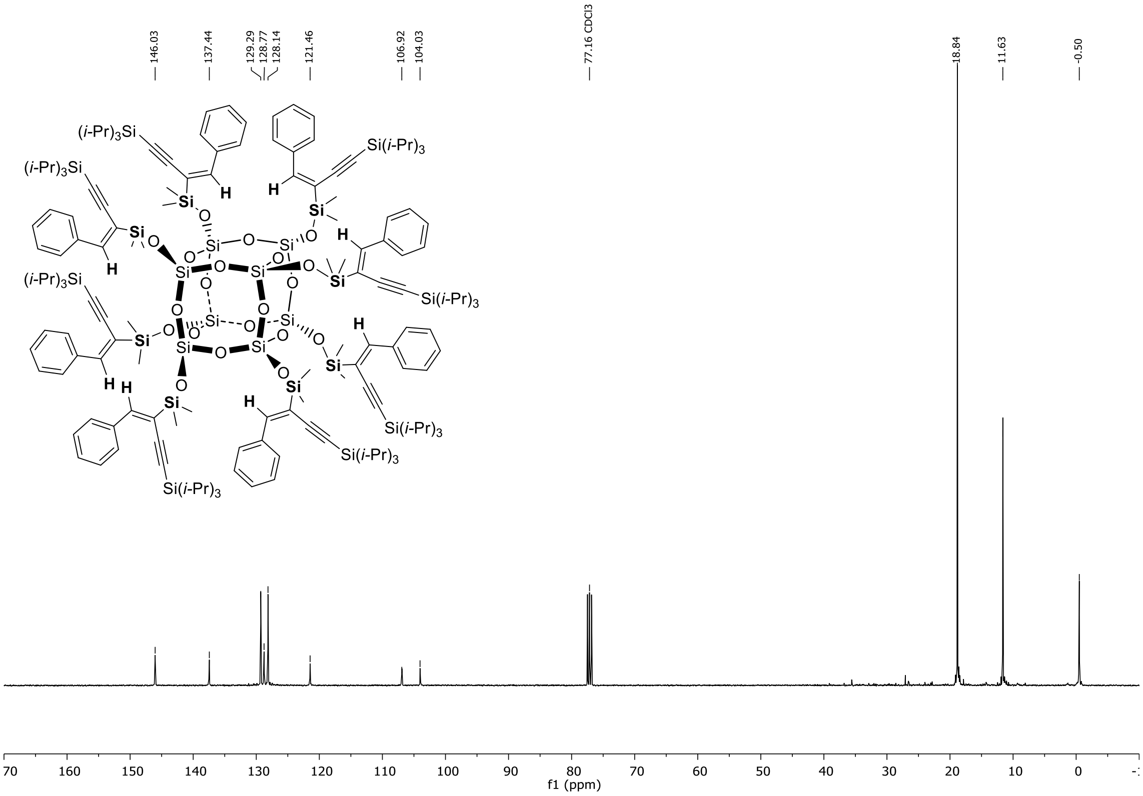

Figure S64. ^{13}C NMR of compound **3f**.

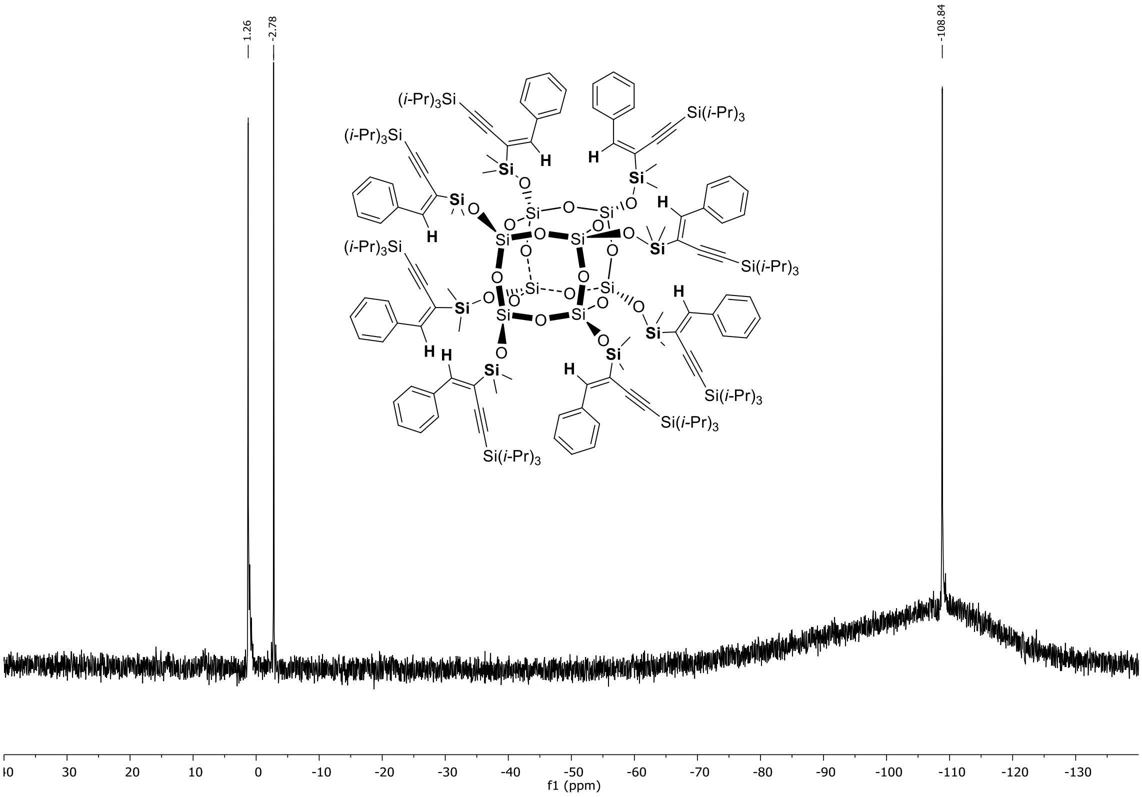

Figure S65. ^{29}Si NMR of compound **3f**.

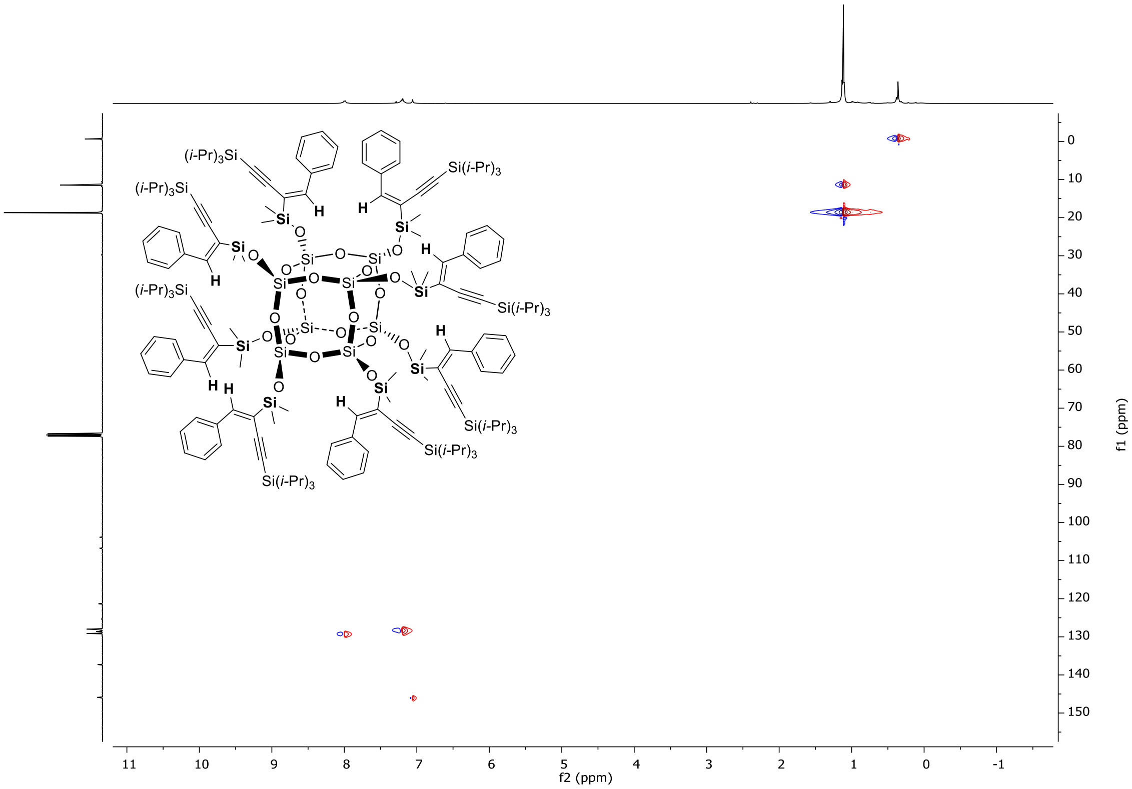

Figure S66. ^{1}H-^{13}C HSQC NMR of compound **3f**.

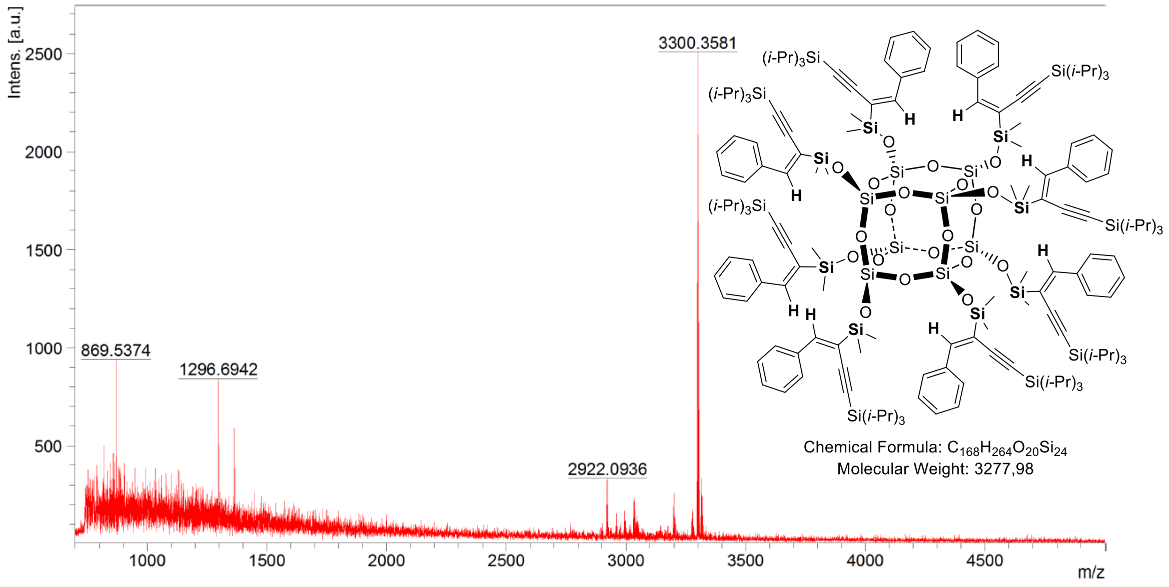

Figure S67. MALDI TOF MS spectra of compound **3f**.

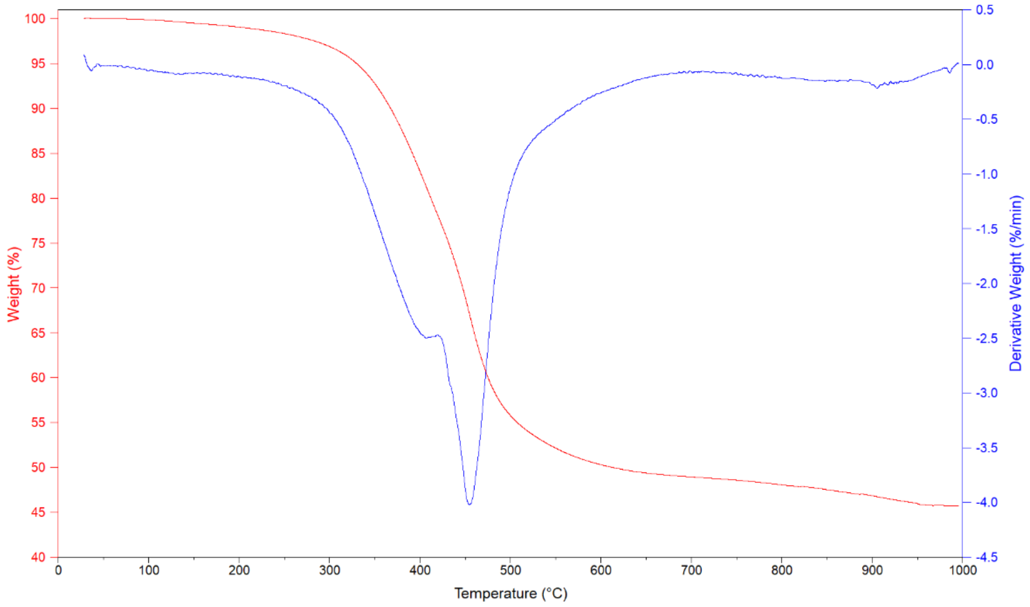

Figure S68. TGA/DTG curves of compound **3f**.

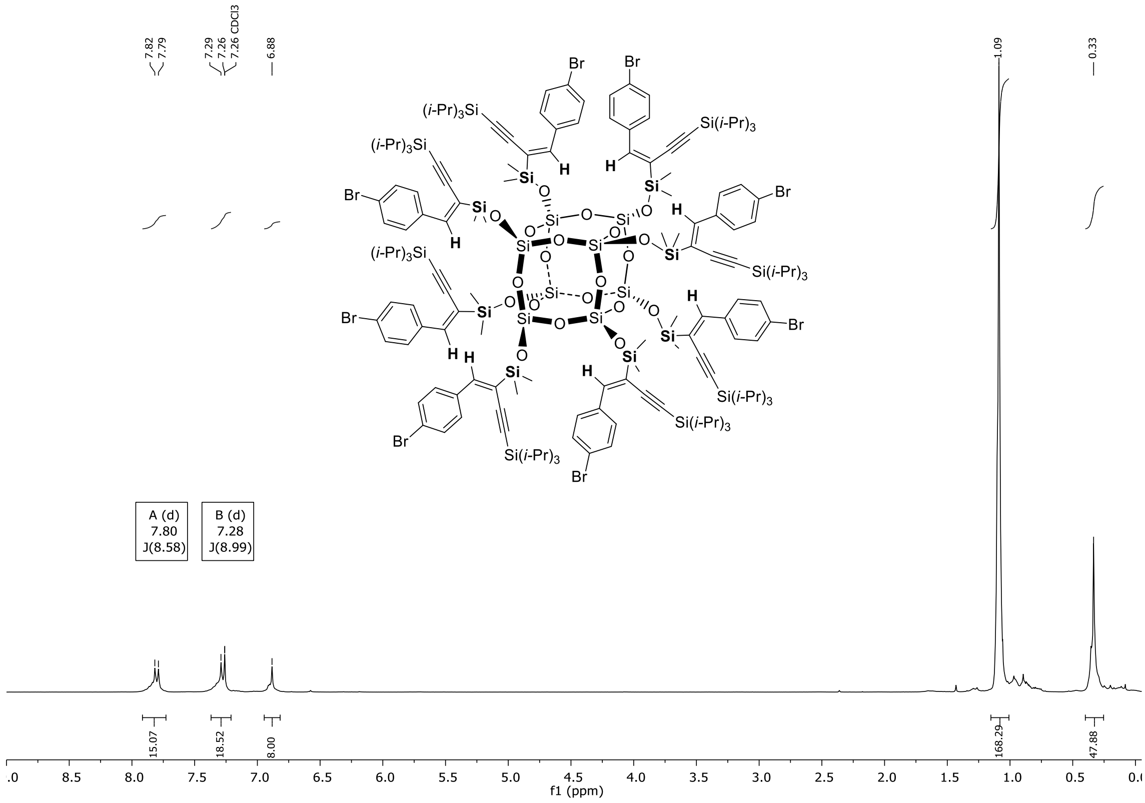

Figure S69. ^{1}H NMR of compound **3g**.

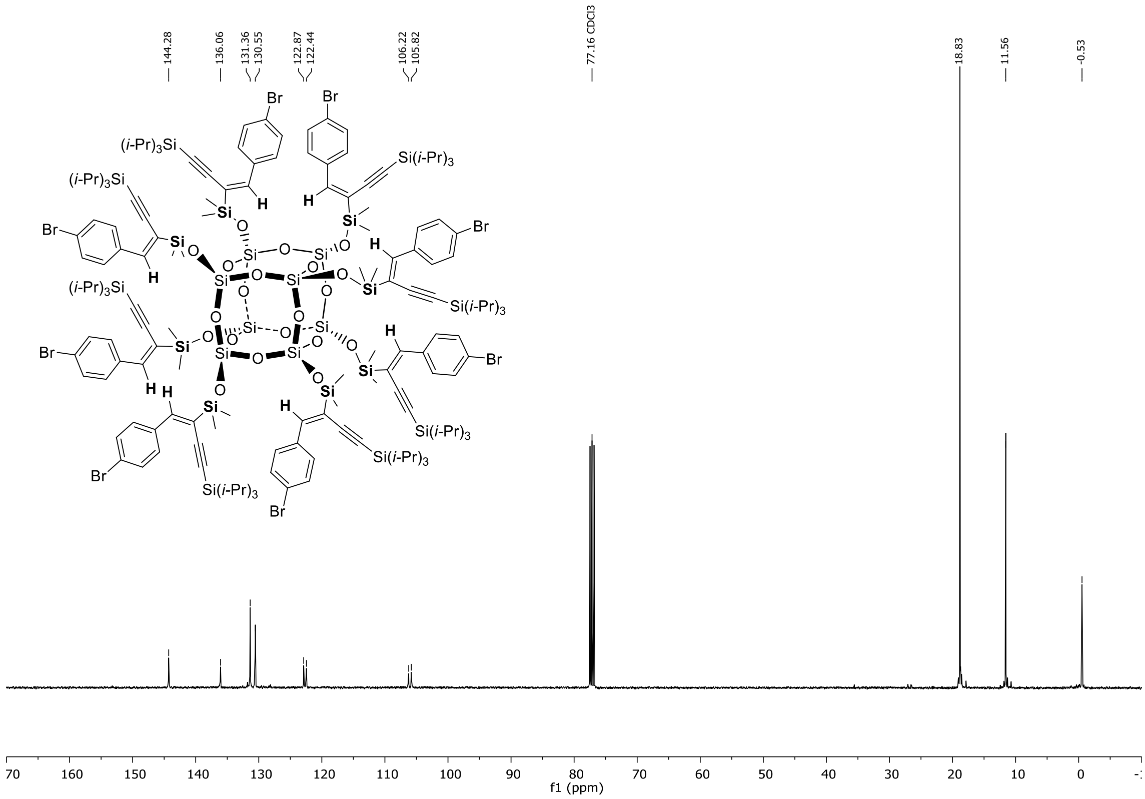

Figure S70. ^{13}C NMR of compound **3g.**

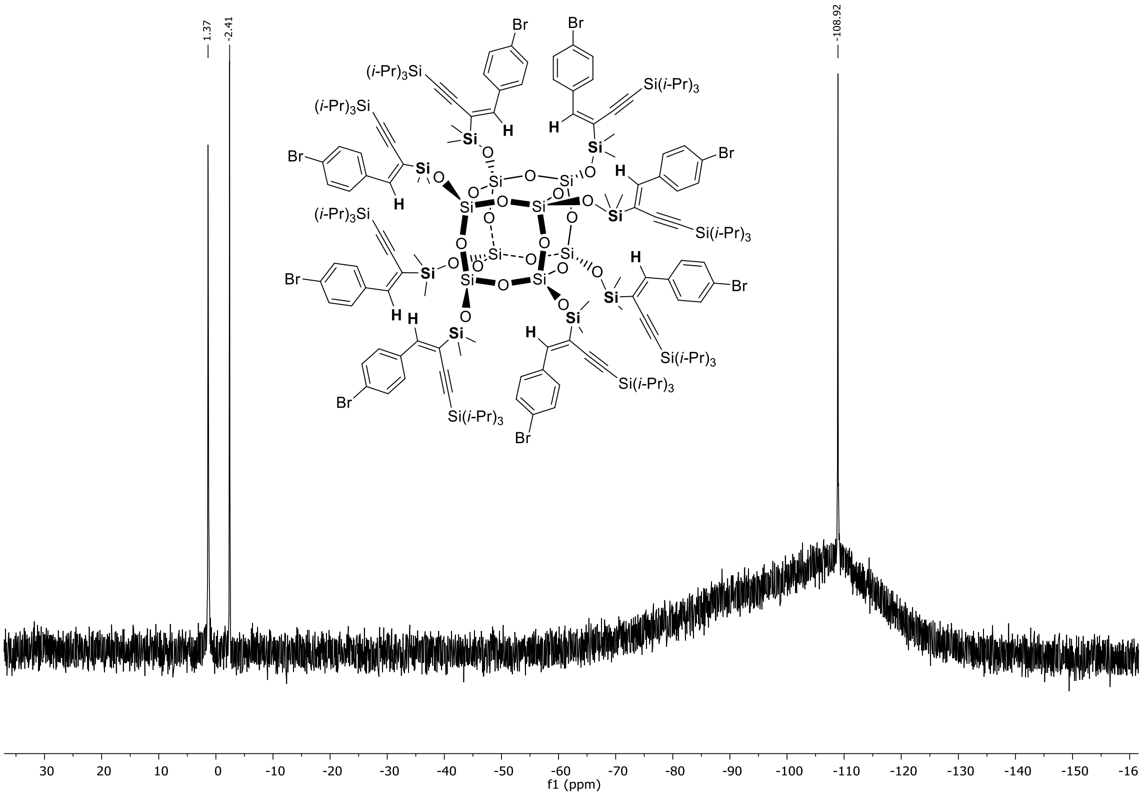

Figure S71. ^{29}Si NMR of compound **3g**.

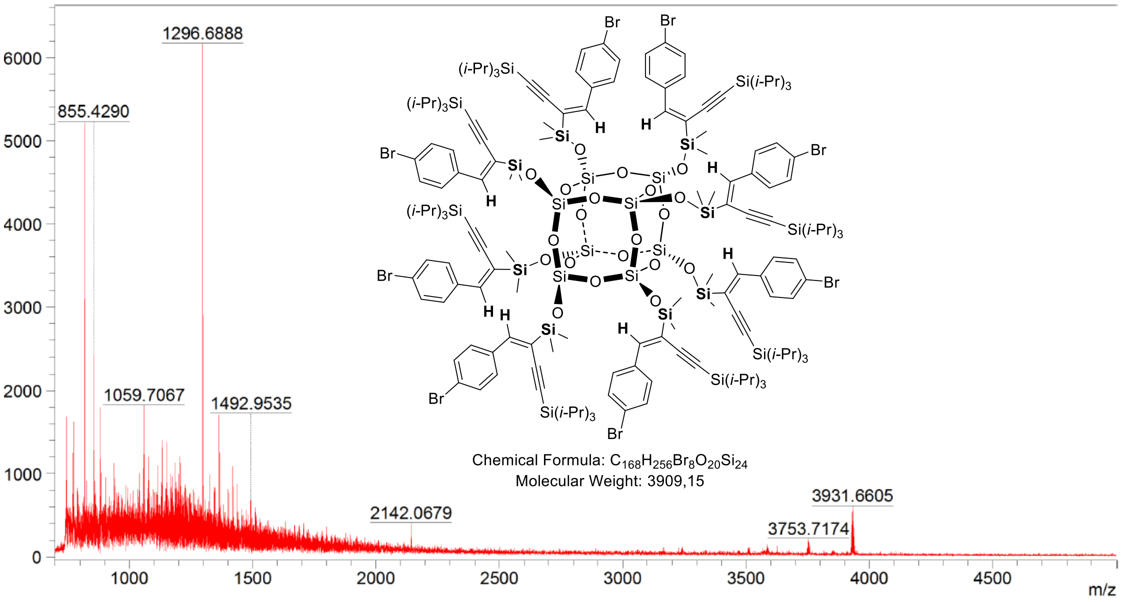

Figure S72. MALDI TOF MS spectra of compound **3g**.

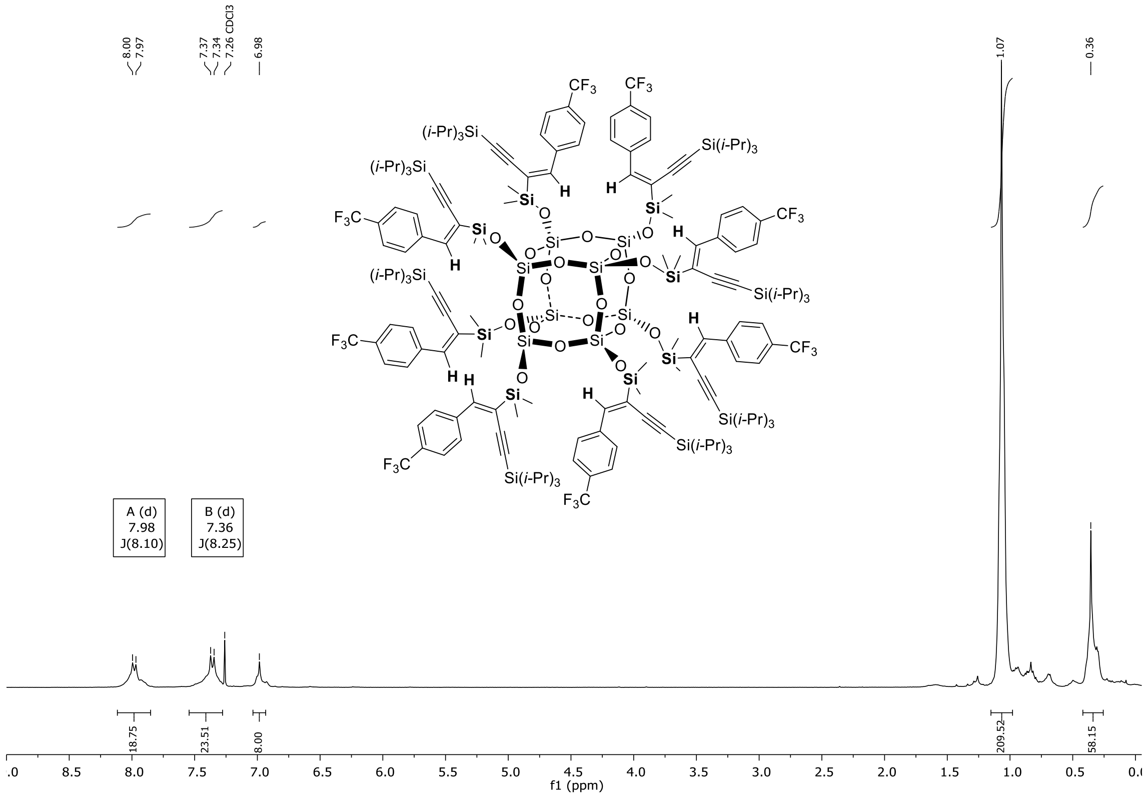

Figure S73. ^{1}H NMR of compound **3h**.

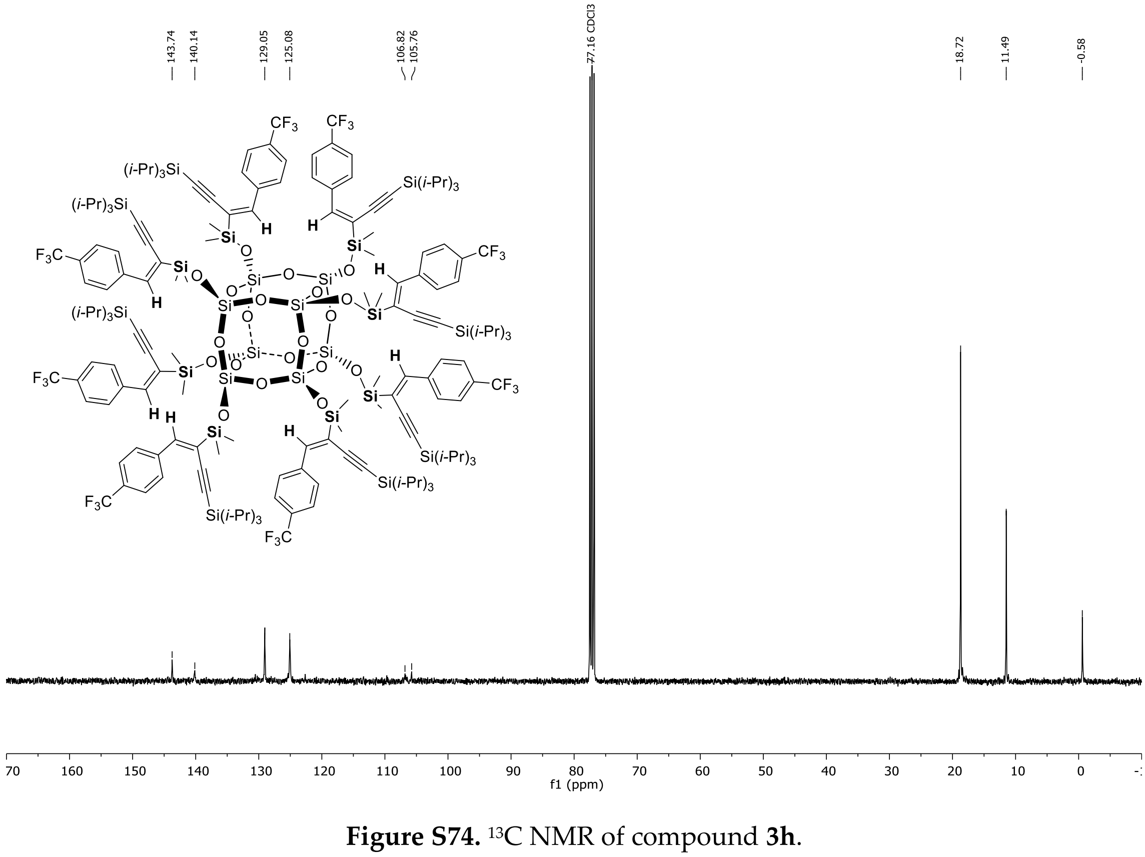

Figure S74. ^{13}C NMR of compound **3h**.

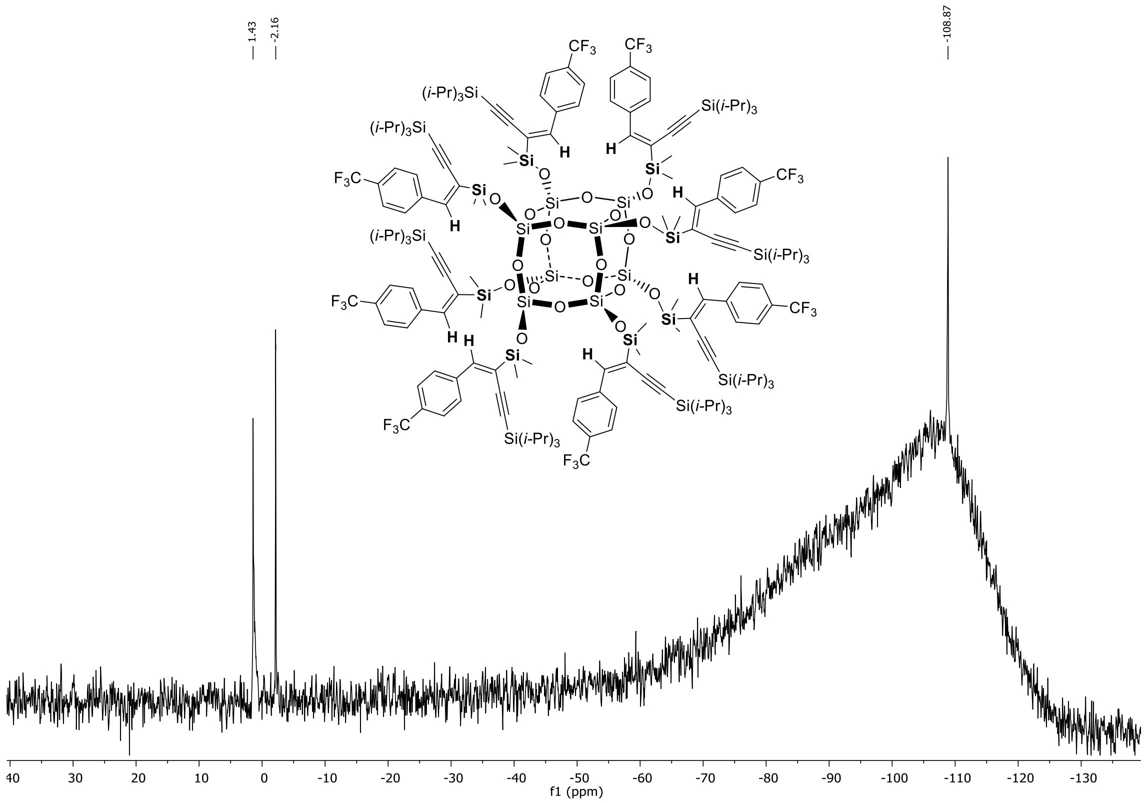

Figure S75. ^{29}Si NMR of compound **3h**.

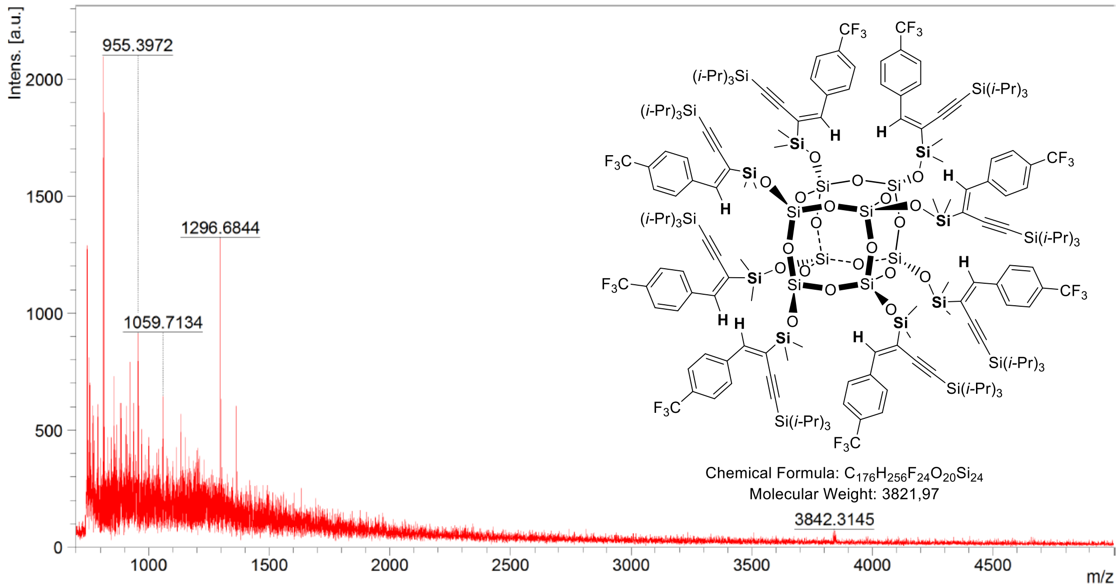

Figure S76. MALDI TOF MS spectra of compound **3h**.

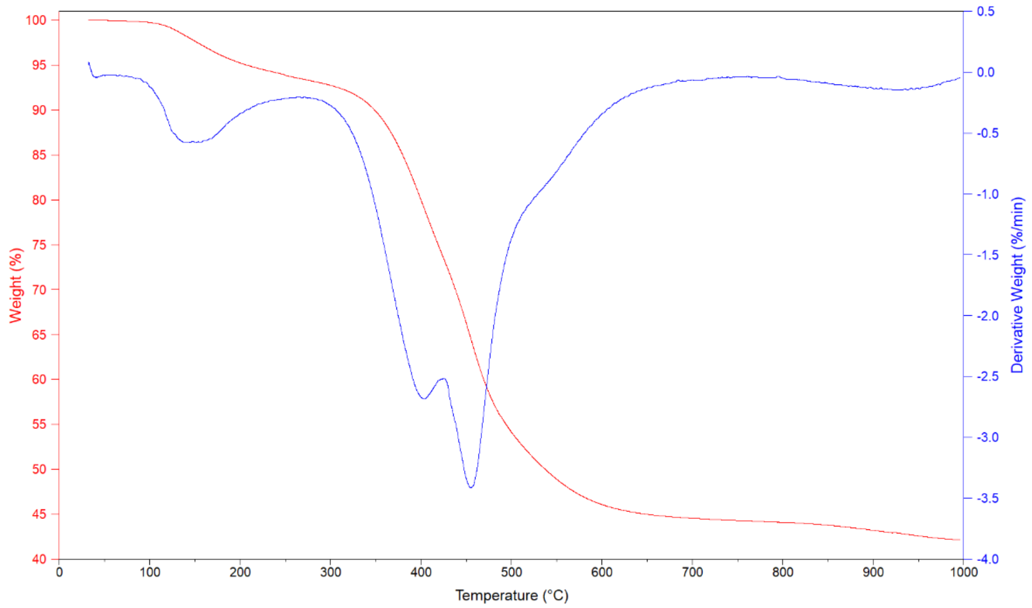

Figure S77. TGA/DTG curves of compound **3h**.

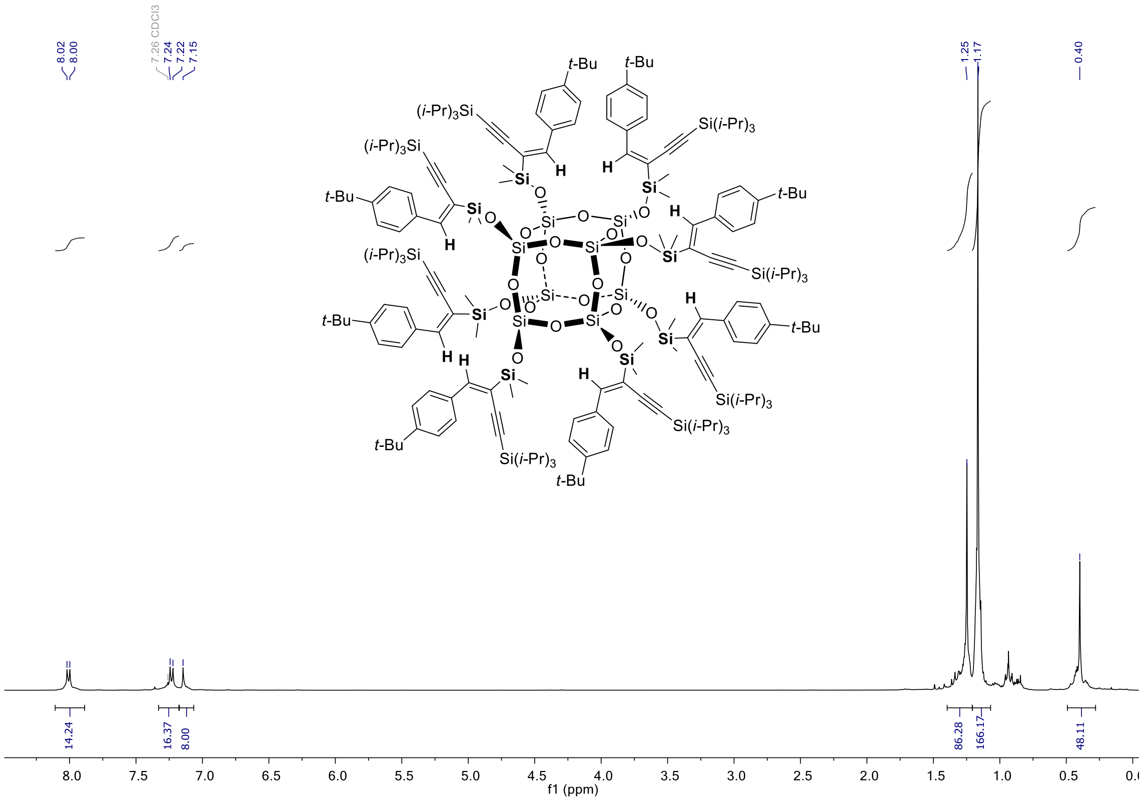

Figure S78. ^{1}H NMR of compound **3i**.

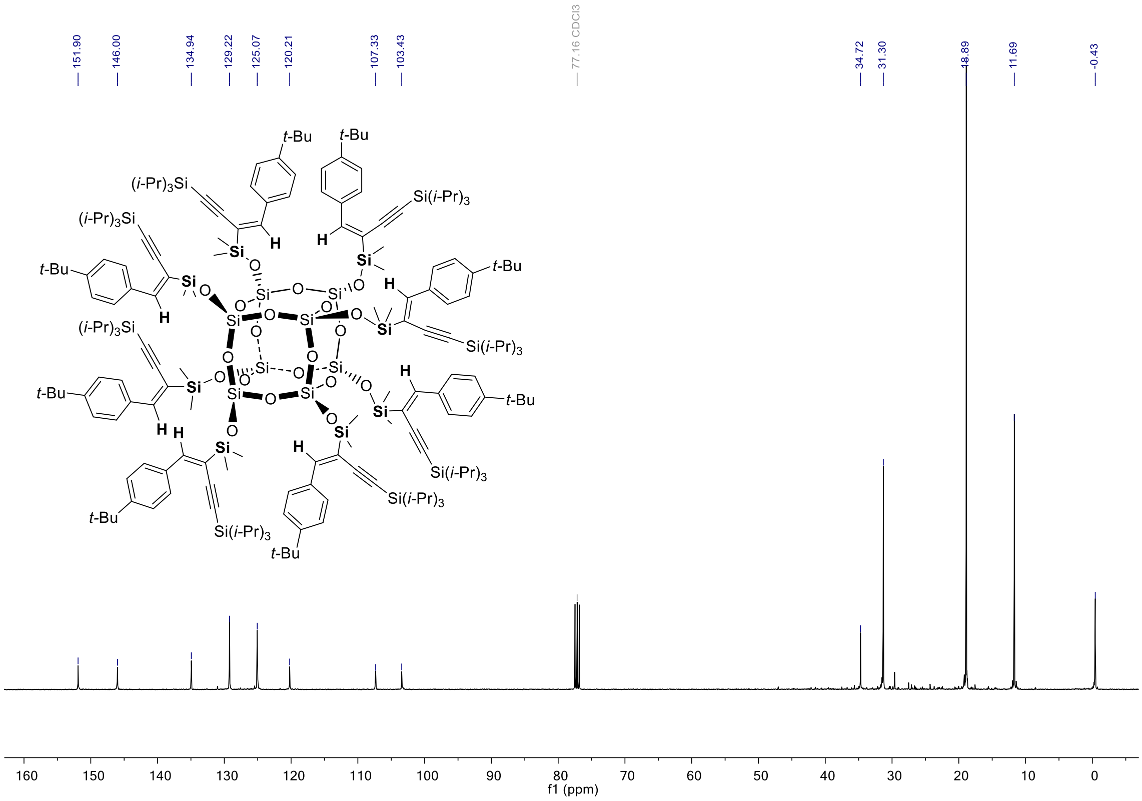

Figure S79. ^{13}C NMR of compound **3i**.

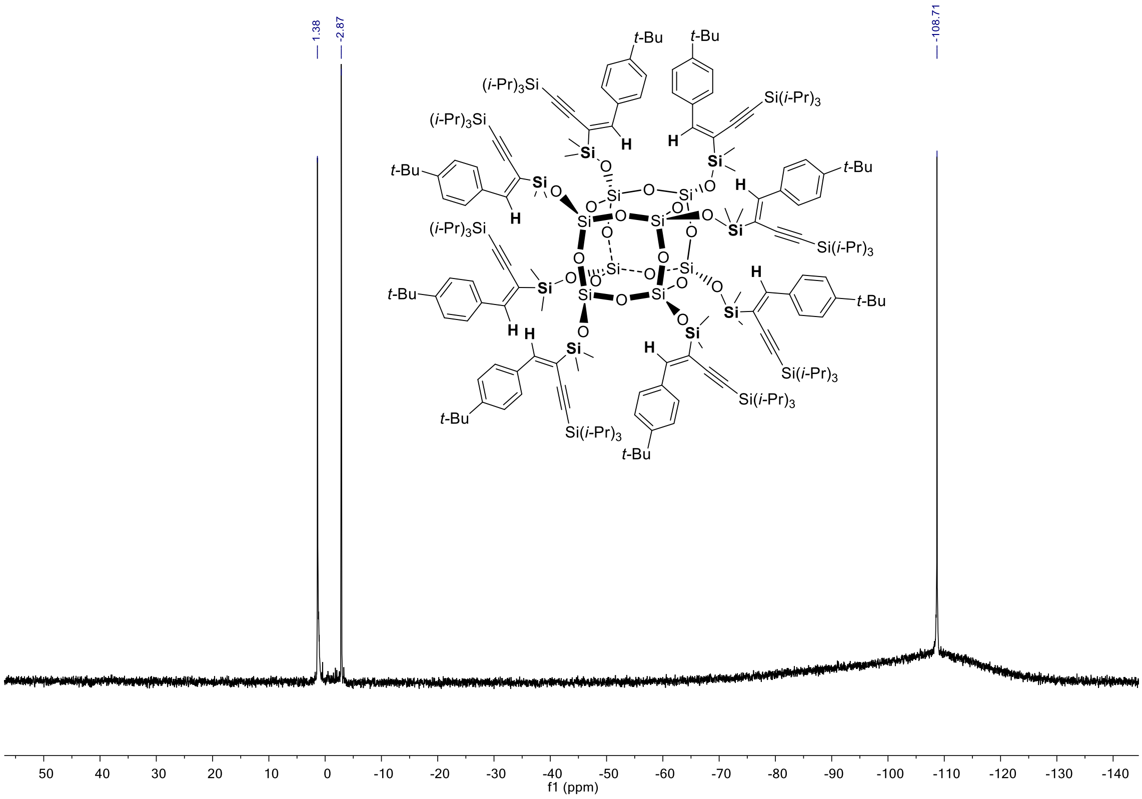

Figure S80. ^{29}Si NMR of compound **3i**.

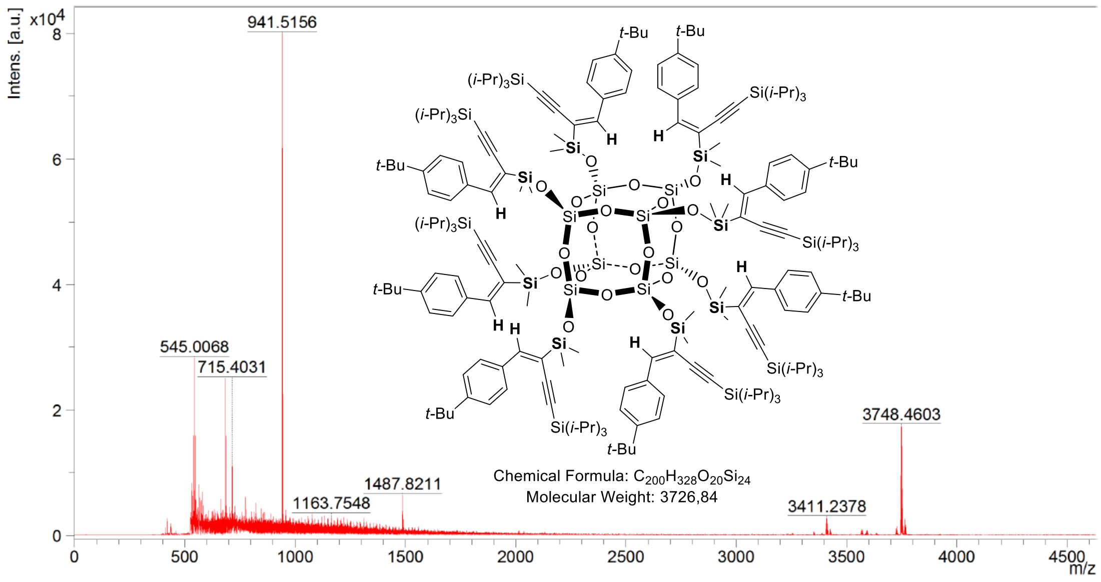

Figure S81. MALDI TOF MS spectra of compound **3i**.

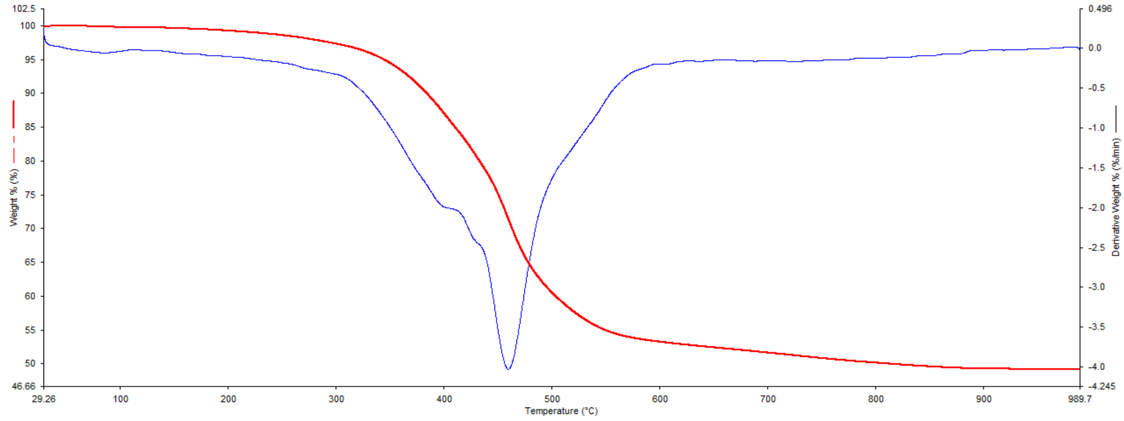

Figure S82. TGA/DTG curves of compound **3i**.

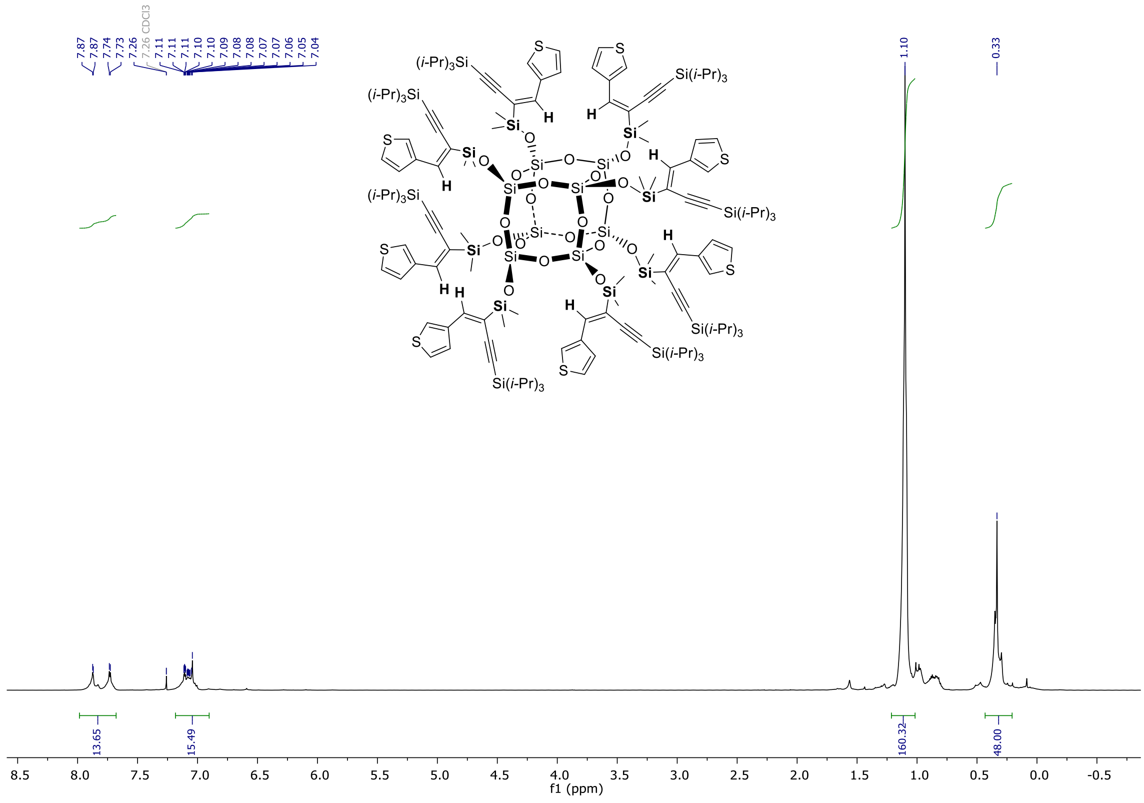

Figure S83. ^{1}H NMR of compound **3j**.

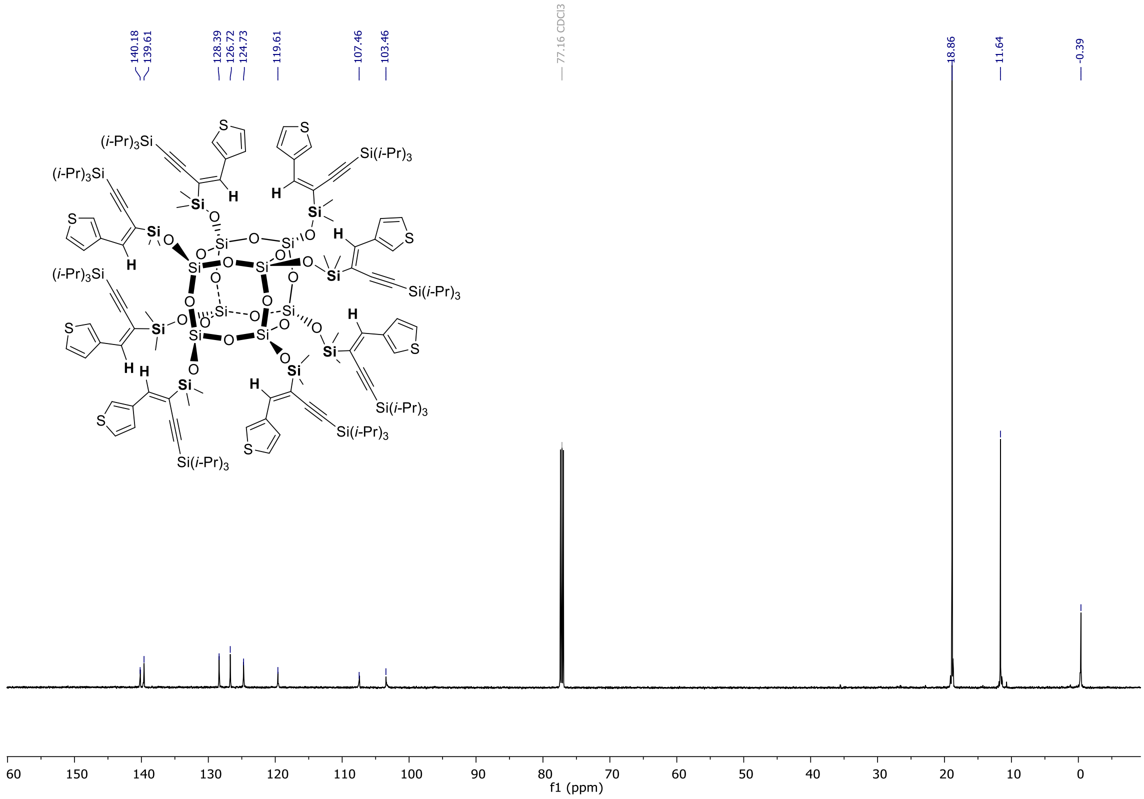

Figure S84. ^{13}C NMR of compound **3j**.

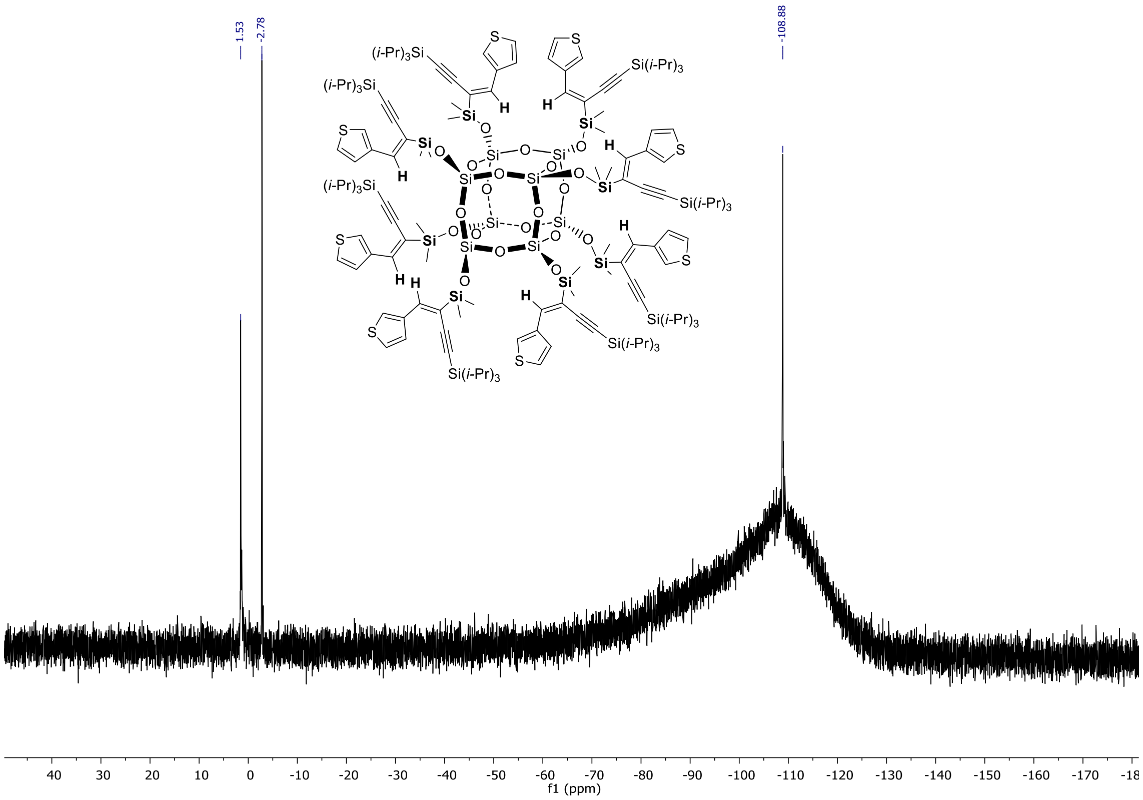

Figure S85. ^{29}Si NMR of compound **3j**.

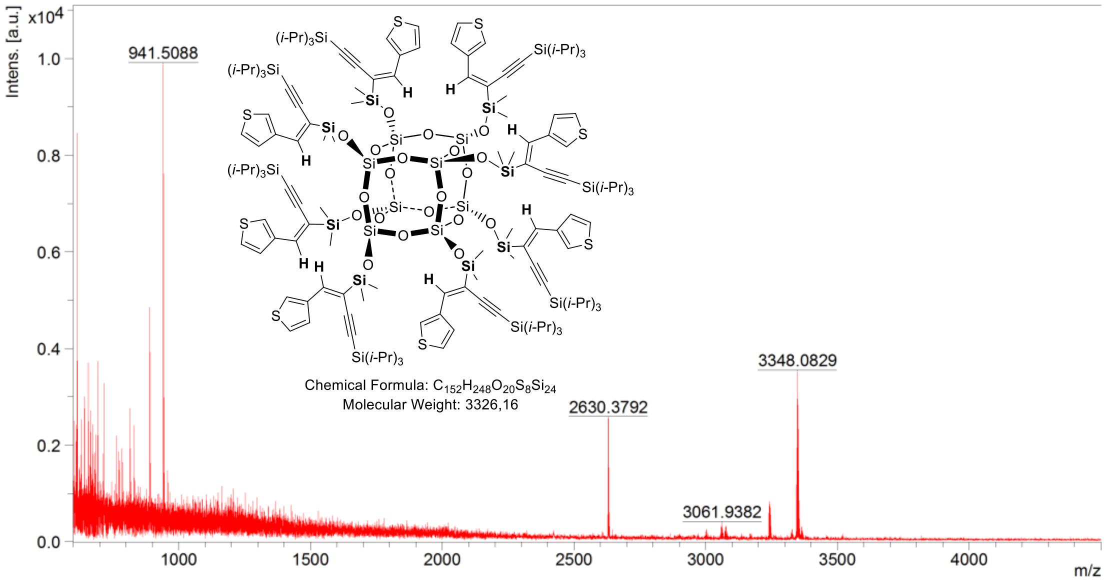

Figure S86. MALDI TOF MS spectra of compound **3j**.

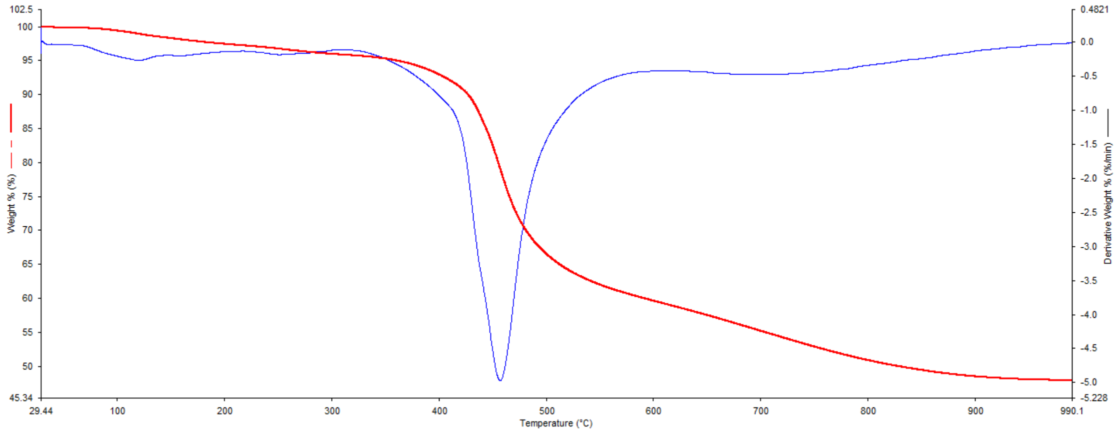

Figure S87. TGA/DTG curves of compound **3j**.

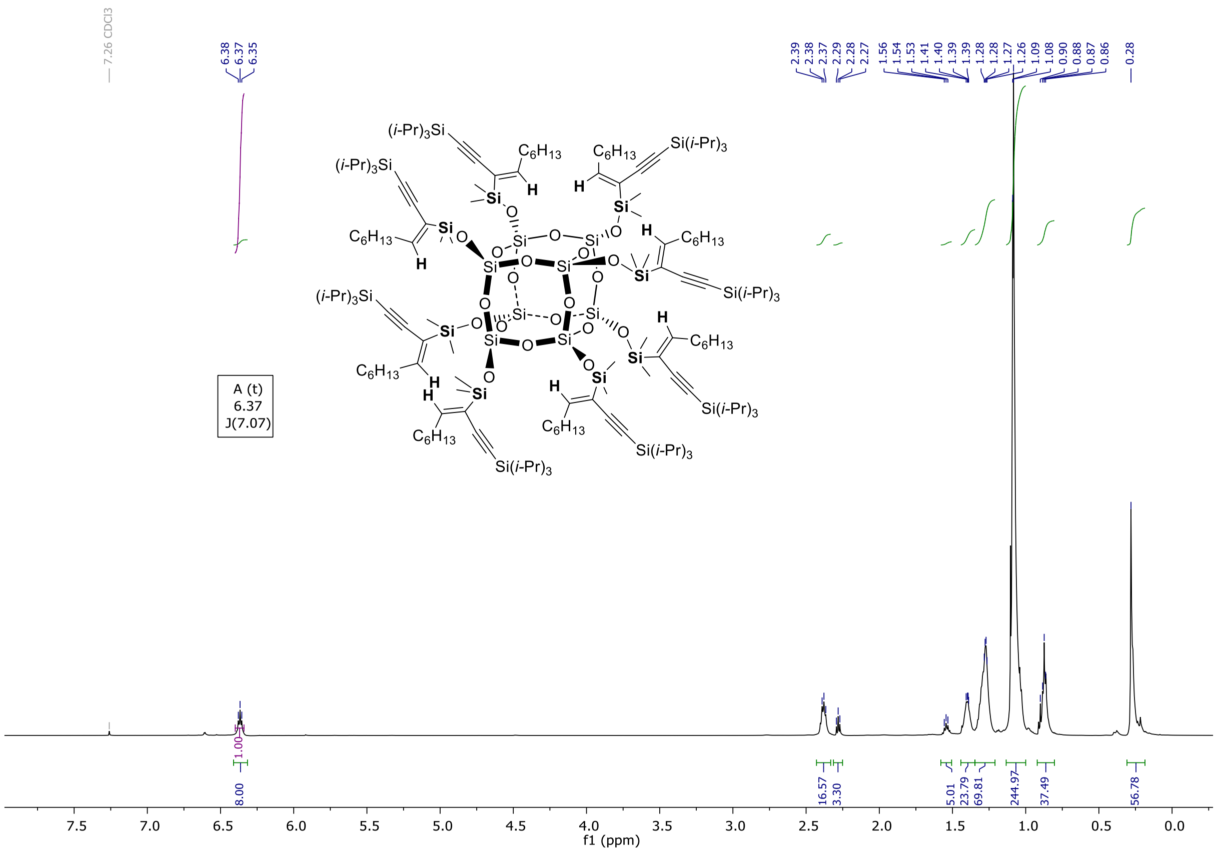

Figure S88. ^{1}H NMR of compound **3k**.

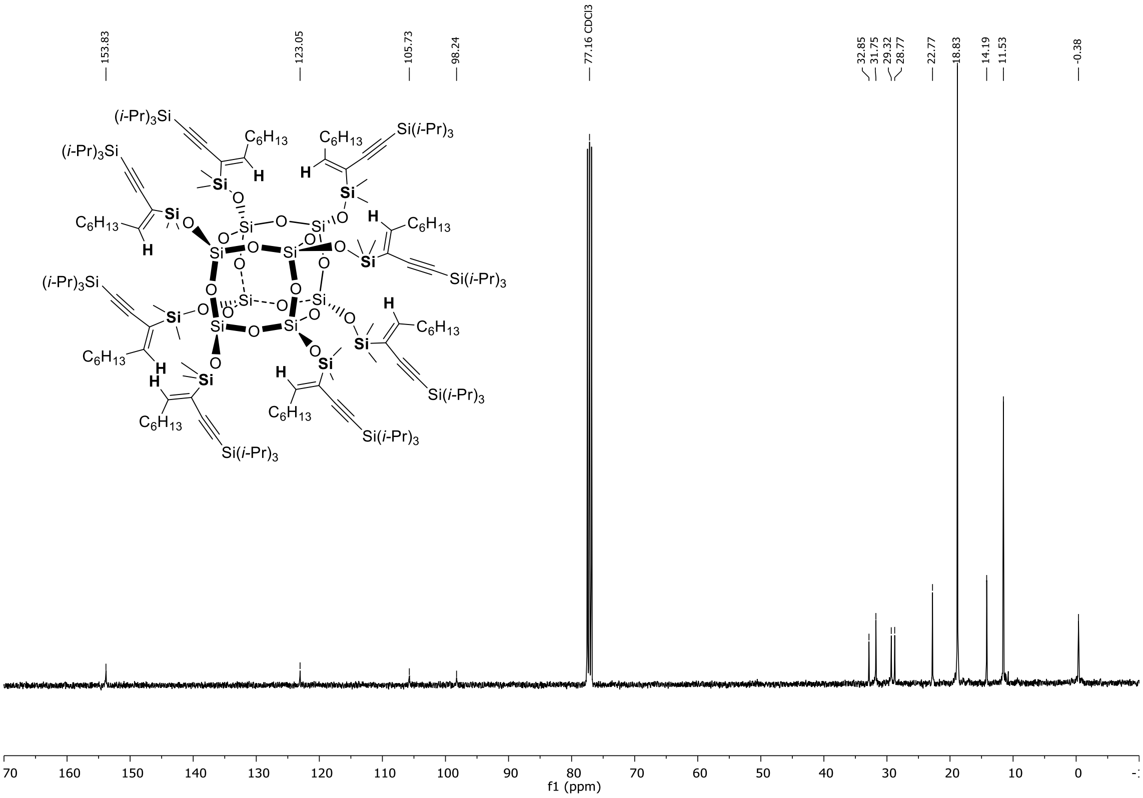

Figure S89. ^{13}C NMR of compound **3k**.

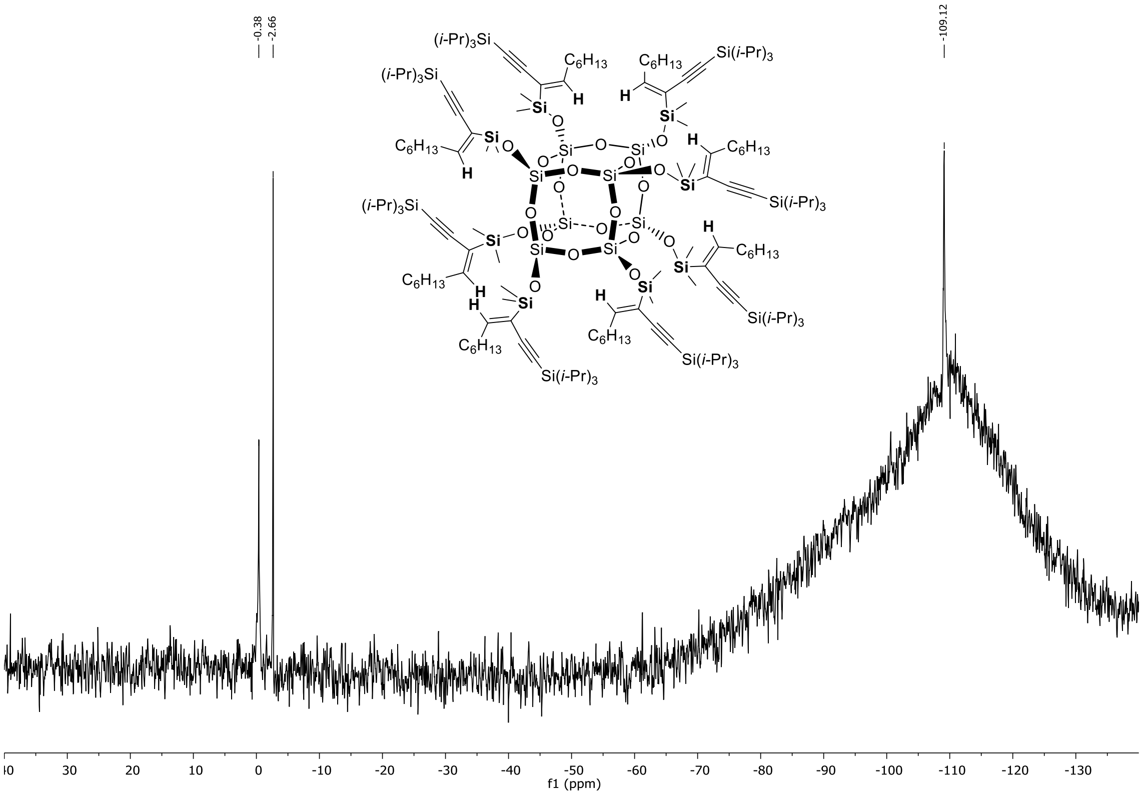

Figure S90. ^{29}Si NMR of compound **3k**.

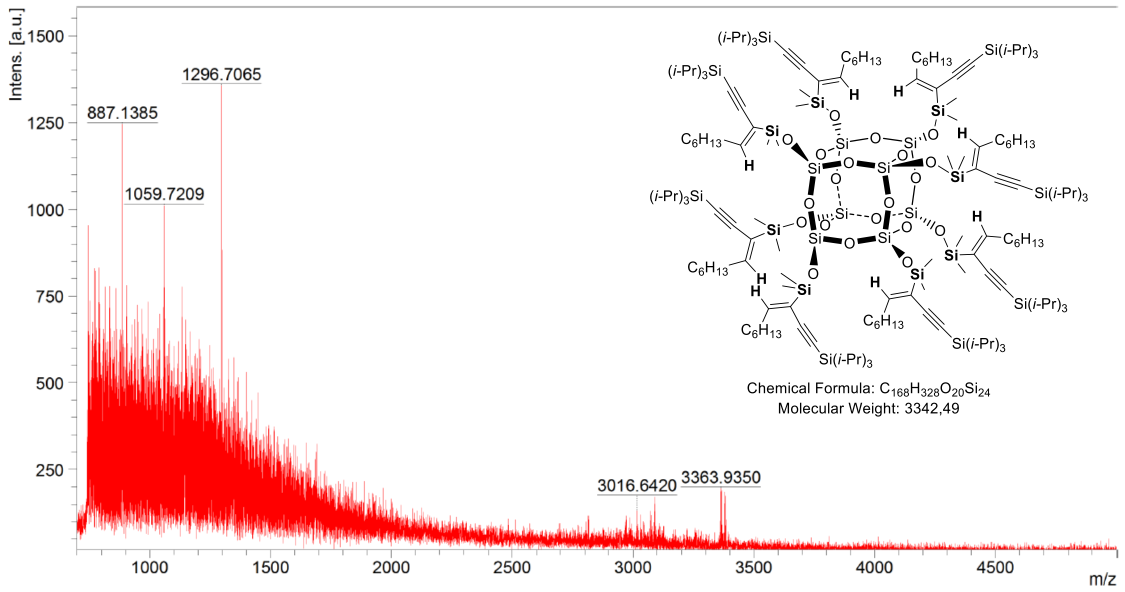

Figure S91. MALDI TOF MS spectra of compound **3k**.

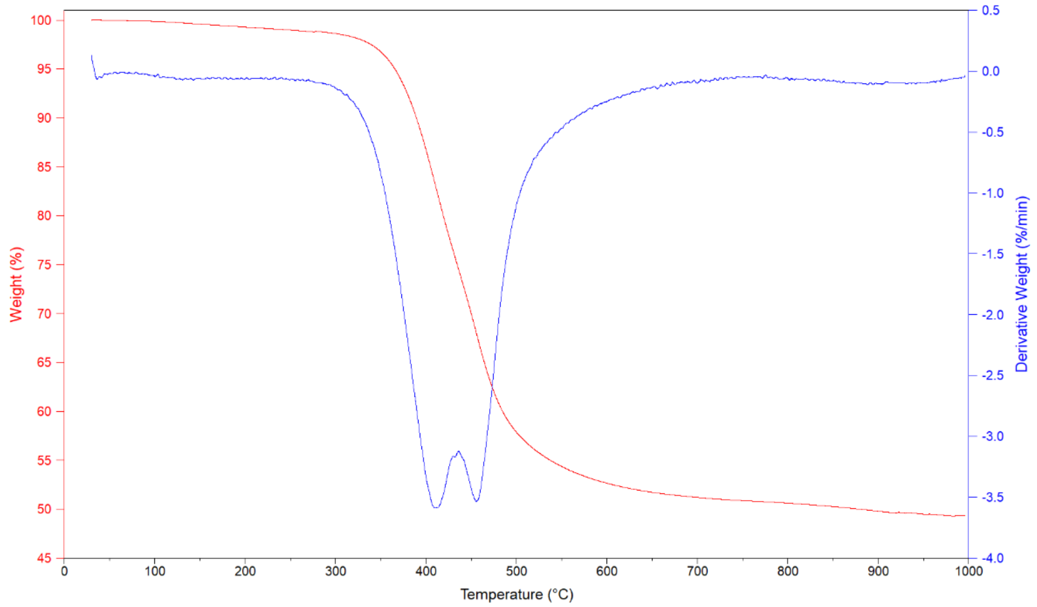

Figure S92. TGA/DTG curves of compound **3k**.

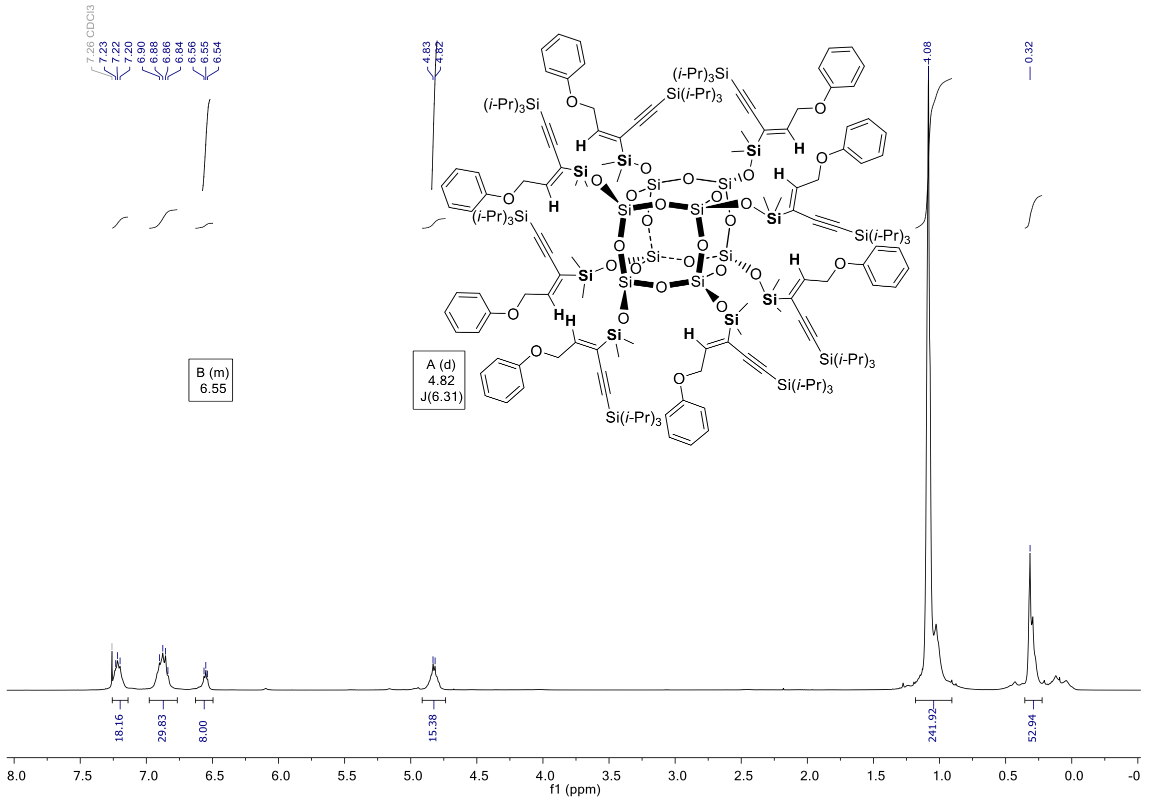

Figure S93. ^{1}H NMR of compound **3l**.

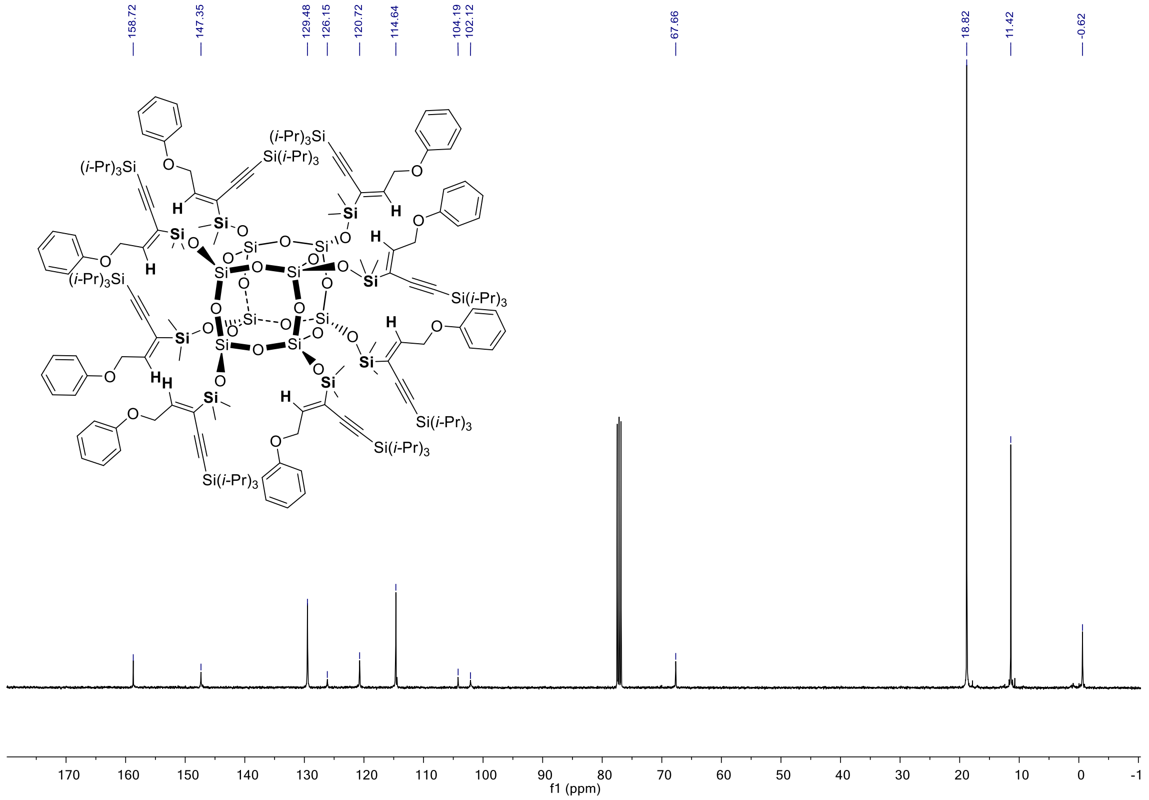

Figure S94. ^{13}C NMR of compound **3l**.

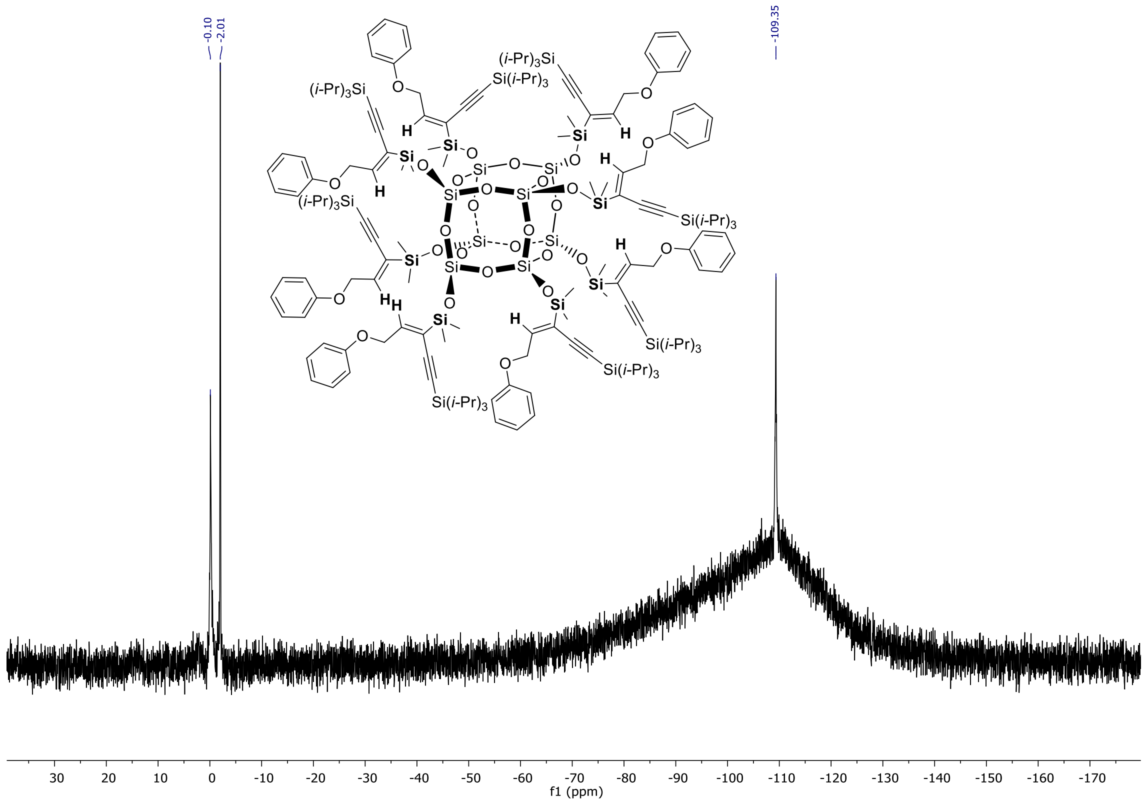

Figure S95. ^{29}Si NMR of compound **3l**.

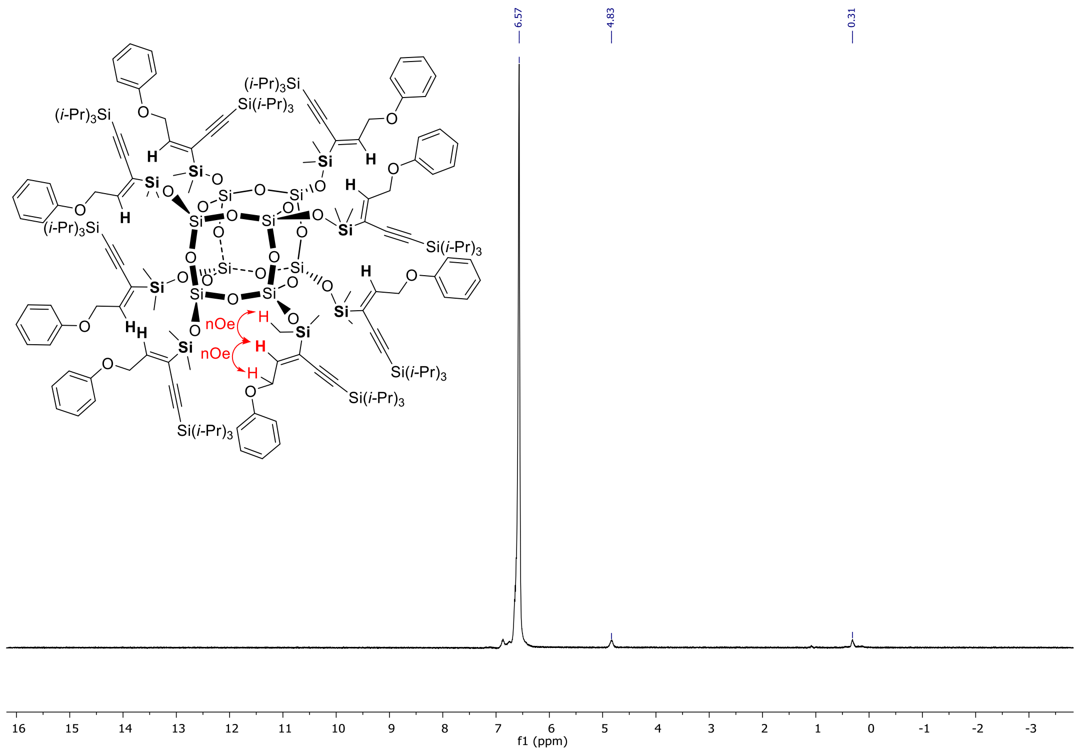

Figure S96. 1D NOE NMR of compound **3l**.

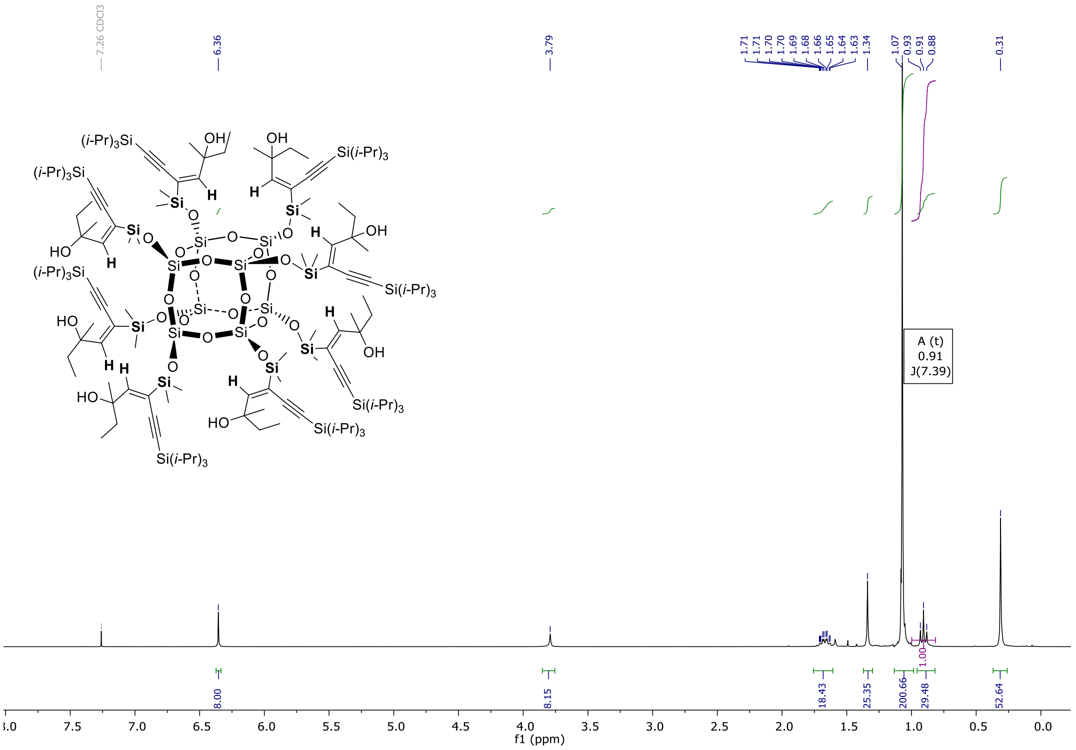

Figure S97. ^{1}H NMR of compound **3m**.

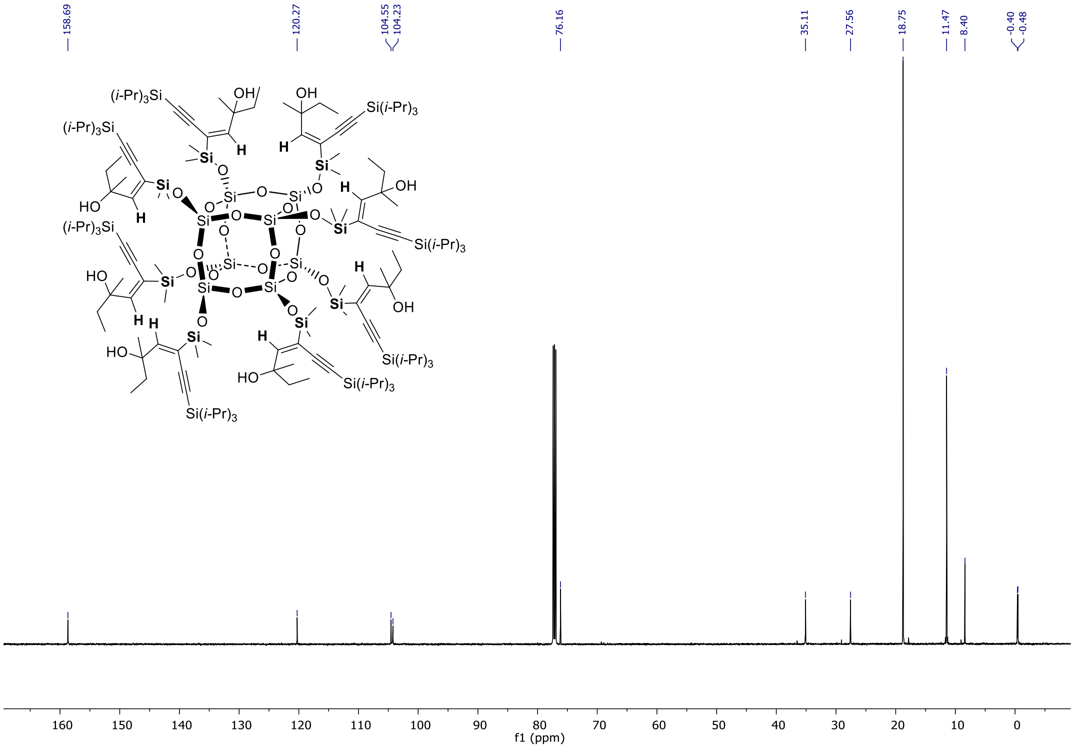

Figure S98. ^{13}C NMR of compound **3m**.

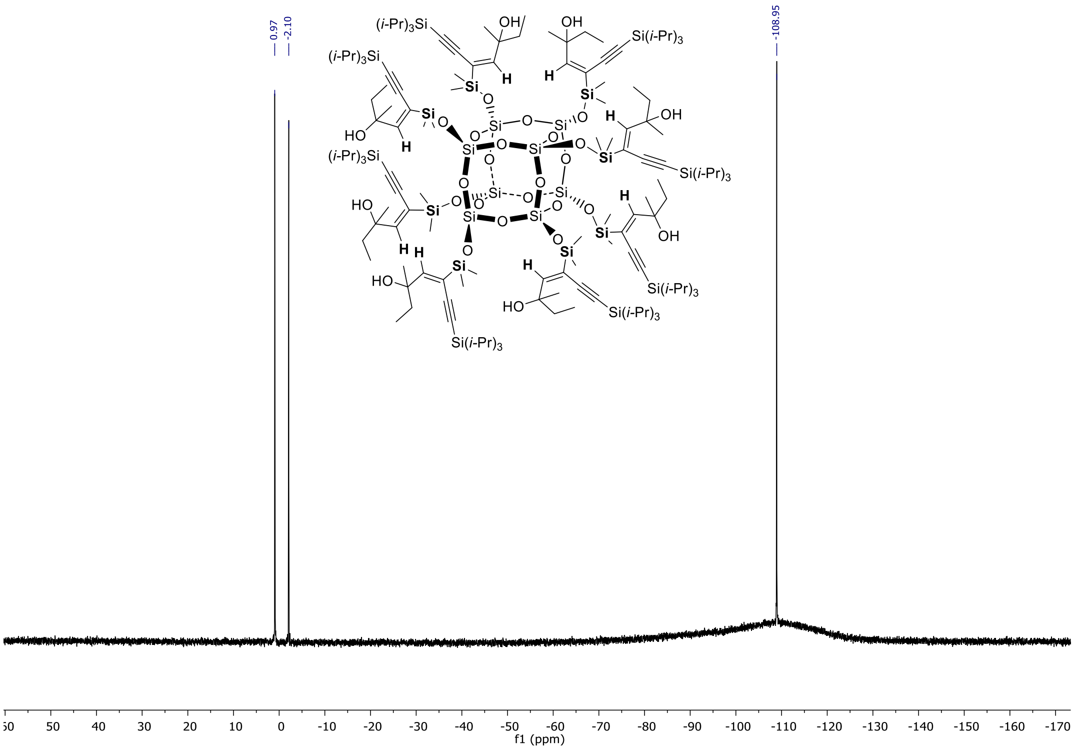

Figure S99. ^{29}Si NMR of compound **3m**.

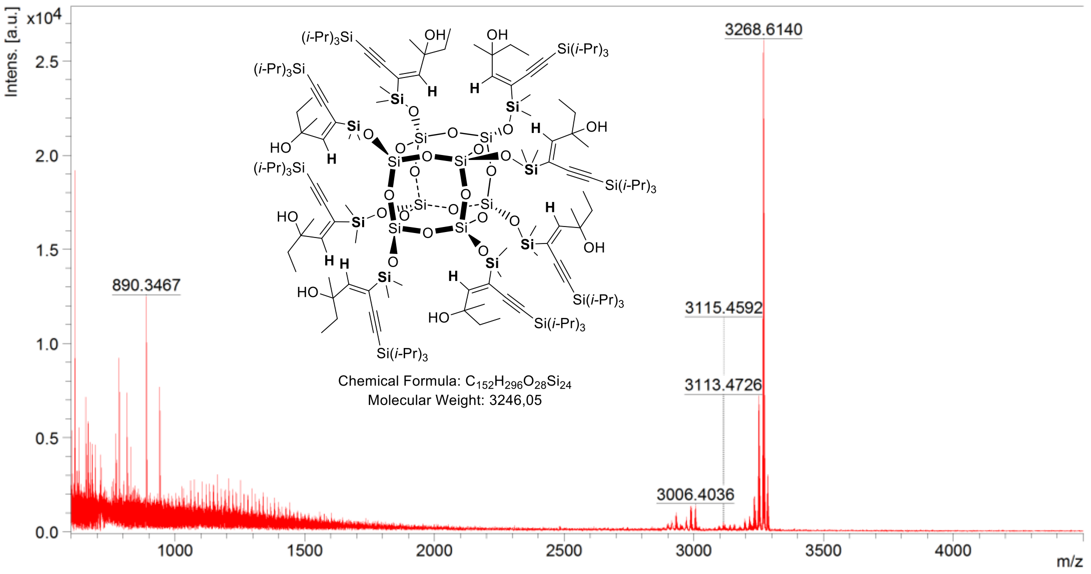

Figure S100. MALDI TOF MS spectra of compound **3m**.

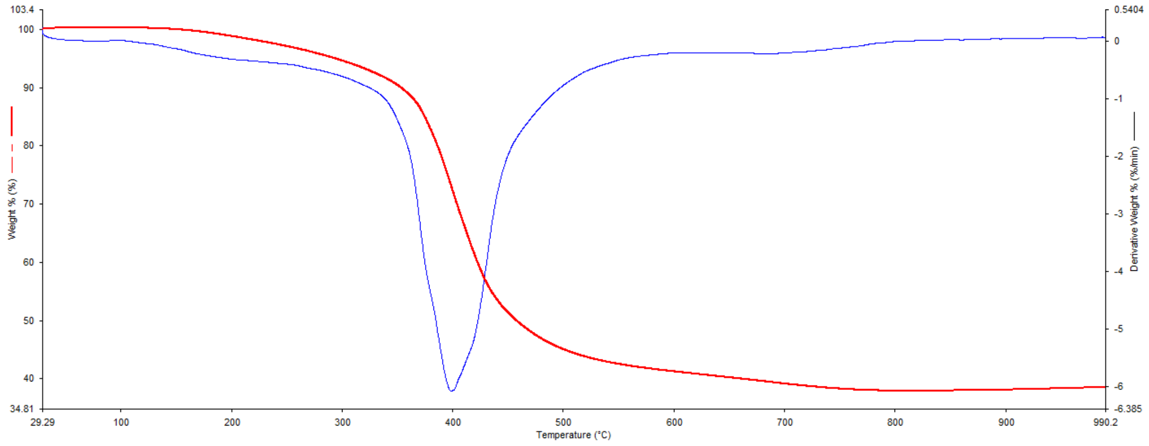

Figure S101. TGA/DTG curves of compound **3m**.

7. Literature

[1.] https://www.mt.com/us/en/home/phased_out_products/L1_AutochemProducts/ReactIR/ReactIR-15.tabs.documents.html

[2.] M. Yamagishi, K. Nishigai, T. Hata and H. Urabe, *Organic Letters*, **2011**, *13*, 4873-4875.

[3.] J. P. Marino and H. N. Nguyen, *The Journal of Organic Chemistry*, **2002**, *67*, 6841-6844.

[4.] M. Arisawa, Y. Tagami and M. Yamaguchi, *Tetrahedron Letters*, **2008**, *49*, 1593-1597.

[5.] X. Chen, H. Zhang, J. Chen and H. Gong, *Chemistry Letters*, **2014**, *44*, 129-131.

[6.] M. P. Smela and T. R. Hoye, *Organic Letters*, **2018**, *20*, 5502-5505.

[7.] J. Schörgenhumer and M. Waser, *Organic & Biomolecular Chemistry*, **2018**, *16*, 7561-7563.

[8.] K. Stefanowska, J. Szyling, J. Walkowiak and A. Franczyk, *Inorganic Chemistry*, **2021**, *60*, 11006-11013.

[9.] K. Kafuta, C. J. Rugen, T. Heilmann, T. Liu, C. Golz and M. Alcarazo, *European Journal of Organic Chemistry*, **2021**, *2021*, 4038-4048.

[10.] K. Takahashi, S. J. Geib, K. Maeda, D. P. Curran and T. Taniguchi, *Organic Letters*, **2021**, *23*, 1071-1075.

[11.] J. Szyling, A. Szymańska, A. Franczyk and J. Walkowiak, *The Journal of Organic Chemistry*, **2022**, *87*, 10651-10663.
